# Molecular basis of type VI secretion system effector loading

**DOI:** 10.1038/s41564-026-02363-x

**Published:** 2026-05-27

**Authors:** Patricia Paracuellos, Ambre Bexter, Jonasz B. Patkowski, Steven D. Kelly, Oleksii Omelchenko, Kévin Macé, Aravindan Ilangovan, Sujatha Subramoni, John C. Whitney, Alain Filloux, Tiago R. D. Costa

**Affiliations:** 1 Centre for Bacterial Resistance Biology, Imperial College, London, UK; 2 Department of Life Sciences, Imperial College, London, UK; 3 Department of Biochemistry and Biomedical Sciences, Michael DeGroote Institute for Infectious Disease Research, McMaster University, Hamilton, Ontario Canada; 4 Institut de Génétique et Développement de Rennes, Université de Rennes and CNRS, Rennes, France; 5 School of Biological and Chemical Sciences, Queen Mary University of London, London, UK; 6 Singapore Centre for Environmental Life Sciences Engineering, Nanyang Technological University, Singapore, Singapore; 7 School of Biological Sciences, Nanyang Technological University, Singapore, Singapore; 8 Lee Kon Chian School of Medicine, Nanyang Technological University, Singapore, Singapore

**Keywords:** Cryoelectron microscopy, Bacterial toxins

## Abstract

Type VI secretion systems (T6SSs) are widespread bacterial nanomachines that deliver effectors into prokaryotic and eukaryotic cells. How an effector cargo is recruited and loaded into the Hcp ring assemblies that form the tube injected by the T6SS remains poorly understood. *Pseudomonas aeruginosa* has four T6SSs, each associated with a different Hcp protein. Here we use cryo-electron microscopy to resolve the structure of the Tce1 cargo loaded into a Hcp3 ring from the *P. aeruginosa* H3-T6SS. We show that a single Tce1 monomer interacts asymmetrically with, and is enclosed by, two hexameric Hcp3 rings, engaging key residues lining the inner surface of the Hcp3 disc. Our data indicate a stepwise loading mechanism, where an initial heterodimeric Hcp–cargo complex forms before ring encapsulation around the effector. Structural modelling suggests similar effector–Hcp3 interactions for a second T6SS effector, Tce2, which has antifungal activity. We propose that this mechanism enables coordinated delivery of a broad payload into target cells.

## Abstract (web summary)

Structural analysis reveals that the type VI secretion system effector cargo is enclosed within hexameric Hcp3 rings that form sequentially to enable effector loading and delivery by the *Pseudomonas aeruginosa* H3-type VI secretion system.

## Main

Microbes have been competing for billions of years, and this has driven the evolution of numerous strategies to capture scarce resources and eliminate competitors^1^. The type VI secretion system (T6SS) is a dynamic bacterial supramolecular machine that injects toxic effectors into target cells^2–4^. T6SSs are versatile and target many cell types^5^ such as eukaryotic cells, including macrophages^6^, epithelial^7^ and goblet^8^ cells or fungi^9^, or prokaryotes such as Gram-negative bacteria^10^. This nanomachine is like a crossbow loaded with toxic arrows and there is extensive knowledge on how its assembly is initiated and the structure is extended and then contracted to fire effectors into susceptible cells^11,12^. The T6SS tip is a puncturing device, a spike complex comprising PAAR and VgrG proteins^13^. The polymerization and extension of a cytosolic contractile T6SS sheath is a coordinated event^14^ during which hexameric rings of the Hcp^15,16^ protein first interact with the flat base of the VgrG–PAAR spike complex and then stack on top of one another to form a tube structure that is encapsulated within a sheath^17^. The rapid contraction of the sheath^18^ propels the PAAR–VgrG–Hcp puncturing device through the bacterial envelope and into target cells, delivering effectors along with it.

Despite many advances in visualizing the structure and dynamics of the T6SS apparatus, gaps remain for understanding mechanisms by which toxins/effectors are recruited and loaded into the T6SS. For example, few effectors can be targeted to the T6SS through conserved motifs called MIX, RIX, PIX, WHIX or FIX^19,20^ but how each of them functions at a molecular level has not yet been examined. Some effectors require the assistance of chaperones or adaptors such as Eag or Tap proteins^21,22^, respectively, which may help prevent premature folding in the producing cell cytoplasm. T6SS effector loading has been proposed to occur by attachment of effectors to one component of the PAAR–VgrG–Hcp complex^13^. Effectors can either exist as C-terminal extension of any of these T6SS elements, in which case they are termed specialized effectors, or recruited to one of them and denoted cargo effectors^23^. In general, the association of effectors with VgrG, for example, *Escherichia coli* Tle1 (ref. ^24^), and PAAR, for example, *Pseudomonas aeruginosa* Tse6 (ref. ^25^), is well described. VgrG and PAAR^26,27^, a trimer and a monomer, respectively, can load a maximum of four effectors onto a single T6SS spike complex. By contrast, Hcp rings exists in more than 100 copies within the elongated T6SS sheath^28–30^, increasing the ability of the system to deliver a cocktail of functionally distinct effectors. T6SS loading through Hcp may result in a highly effective system against any type of prey cells, but little is known about how toxins or effectors are recruited to Hcp. Interestingly, Hcp has been proposed to exhibit chaperone activity by stabilizing small effectors such as *P. aeruginosa* Tse2. Based on electron microscopy (EM) low-resolution images, it was shown that Tse2 sits within the lumen of Hcp hexameric rings^31^.

*P. aeruginosa* is equipped with four T6SSs known as the H1-, H2-, H3- and H4-T6SSs^32,33^, and each system is associated with cognate Hcp proteins, Hcp1, Hcp2, Hcp3 and Hcp4, respectively. In previous work, using in vivo pulldown experiments, we identified potential cargo effectors associated with several of these Hcp proteins^34,35^. Building on this work, we herein describe two T6SS-delivered putative cysteine protease effector (Tce) proteins bound to Hcp3: Tce1 (PA0256) and Tce2 (PA2372). We present a high-resolution structure of an Hcp–cargo effector complex using cryo-EM. The 3.8-Å resolution structure of Hcp3–Tce1 reveals the molecular details behind Hcp3 effector recognition, the molecular events that lead to the formation of the complex and their stoichiometric arrangements. Our data indicate that a 1:1 Hcp3–Tce1 heterocomplex forms first, followed by the sequential oligomerization of Hcp subunits around the effector until the final Hcp3 hexameric ring filled with the Tce1 effector is formed. We show that the loading of a T6SS effector requires two rings for it to be fully embedded with the Hcp tube. These findings introduce a concept for T6SS effector loading that is driven by the cellular concentration of Hcp. Overall, our work defines a distinct effector loading mechanism and establishes the molecular principles governing how T6SS cargo complexes are recruited by and loaded into Hcp. These insights may enable the rational engineering of T6SS-wielding bacteria, equipped with a cocktail of Hcp–cargo complexes, for microbiome biocontrol in human and natural ecosystems.

## Results

### Hcp3 interacts with two H3-T6SS effector candidates

**Fig. 1:**
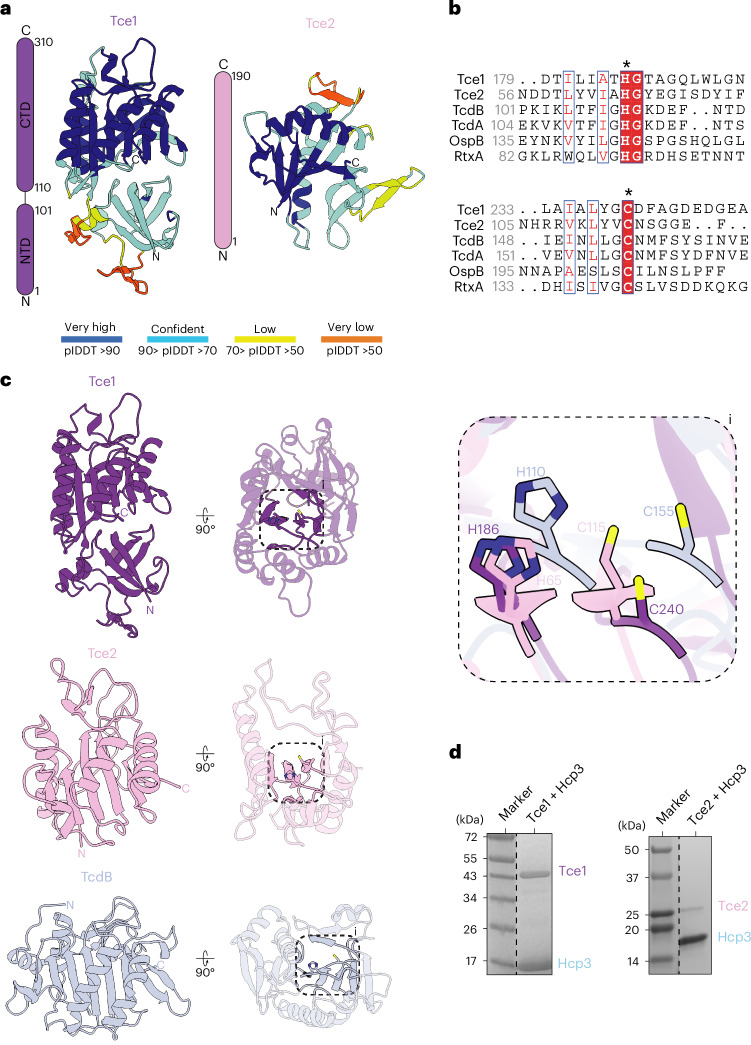
Functional evaluation of the two putative Hcp3 effectors, Tce1 (PA0256) and Tce2 (PA2372). **a**, AlphaFold3 models of the Tce1 and Tce2 structures coloured by pIDDT (sequences accessed from PAO1 genome database). **b**, Sequence alignment of Tce1 with Tce2 and other cysteine proteases TcdB (*C. difficile* cysteine protease PDB:3PEE), a top hit from each of the distance-matrix alignment search performed against Tce1 and Tce2, TcdA (*C. difficile* cysteine protease PDB: 3HO6), OspB (*Shigella* sp. T3SS cysteine protease effector) and RtxA (*V. cholerae* cysteine protease virulence factor). The conserved histidine and cysteine residues across all three proteins are indicated with a star. **c**, Structural comparison of Tce1, Tce2 (AF3 models) and TcdB (PDB: 3PEE) with 90° rotations displaying the shared localization of the conserved catalytic dyad. In (i), an overlap of all three secondary structures to highlight positioning of the catalytic dyad is shown. **d**, SDS–PAGE gels of HA–Tce1 with Hcp3–His and HA–Tce2 with Hcp3–His following nickel affinity chromatography, where the stronger intensity band for Tce1 than Tce2 suggests that more Tce1 is being pulled down with Hcp3 and thus has a stronger interaction. Tce1, Tce2 and Hcp3 originate from *P. aeruginosa* PAO1 and were recombinantly expressed in *E. coli* BL21 (DE3). Each pulldown experiment was performed independently in triplicate. Source data

**Extended Data Fig. 1:**
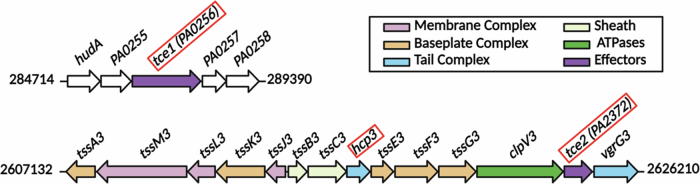
The native arrangement of the H3-T6SS associated genes within the Pseudomonas aeruginosa genome. The arrows represent the genes encoding for the T6SS membrane complex (pink), baseplate complex (orange), tail complex (blue), contractile sheath (yellow), ATPases (green) and effectors (purple). The genes for our two newly identified Type VI Secretion System associated Cysteine protease Effector (Tce) proteins are labelled tce1 and tce2 alongside their gene locus IDs, *PA0256* and *PA2372* respectively.

In a previous study, we identified two potential *P. aeruginosa* PAO1 H3-T6SS effectors—PA0256 and PA2372 (refs. ^34,35^), hereafter referred to as Tce1 and Tce2—through an in vivo pulldown approach using Hcp3, which is encoded within the H3-T6SS gene cluster^35^ (Extended Data Fig. 1). A distance-matrix alignment^36^ was performed using the AlphaFold3^37^ predictions of both Tce1 and Tce2 to identify structural homology to proteins with known enzymatic activity (Fig. 1a). A top hit for each protein was the cysteine protease domain for the *Clostridium difficile* toxin TcdB^38^ (PDB: 3PEE). A multiple sequence alignment of Tce1, Tce2, TcdB and other known cysteine proteases revealed conserved cysteine and histidine residues across these proteins (Fig. 1b). Structural alignment shows that His186 and Cys240 in Tce1, as well as His65 and Cys115 in Tce2, are spatially positioned to align with the catalytic dyad of TcdB (His110 and Cys155), supporting the hypothesis that both Tce1 and Tce2 are putative cysteine protease enzymes (Fig. 1c). It is noteworthy that Tce1 is a larger protein (310 amino acids) than Tce2 (190 amino acids) with a C terminus of unknown function (110–310) encompassing the putative cysteine protease catalytic site and an extended N terminus (1–101) (Fig. 1a). Both effectors co-purify with Hcp3 via affinity chromatography (Fig. 1d), with Tce1 showing a stronger interaction consistent with the in vivo pulldown where Tce1 was tightly bound with Hcp3 (8.4-fold enrichment) compared with Tce2 (5.8-fold enrichment)^35^. Based on these data, we prioritized Tce1 to obtain a high-resolution cryo-EM structure of a Hcp3–effector complex.

### Cryo-EM analyses show two Hcp3 rings fully enclose Tce1

**Fig. 2:**
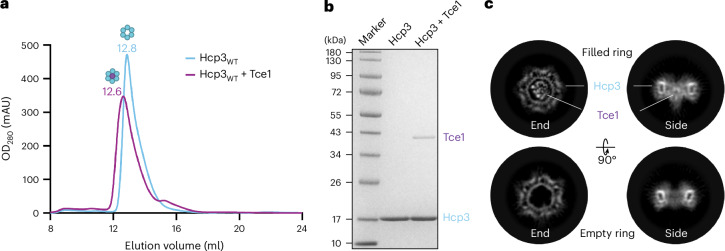
Purification of the Hcp3−Tce1 complex. **a**, SEC chromatograph of purified Hcp3_WT_ alone (blue) and co-purified Hcp3_WT_ and Tce1 (purple). Schematics of the Tce1 filled and empty Hcp3 rings display the assembly of the complex present in each peak. **b**, SDS–PAGE gel of the peak fractions from the chromatograph in **a** stained with Coomassie blue. **c**, End and side view 2D classes of Tce1 filled and empty Hcp3 hexameric rings. Purification of Hcp3_WT_ alone and co-purification of Hcp3_WT_ with Tce1 were performed independently in triplicate. Source data

The *hcp3* and *tce1* genes from *P. aeruginosa* PAO1 were cloned, recombinantly co-expressed in *E. coli* BL21 (DE3), and co-purified via affinity and size-exclusion chromatography (SEC). Hcp3 carries a C-terminal His tag (Hcp3–His) and Tce1 carries an N-terminal HA tag (HA–Tce1). Hcp3 and Tce1 remained associated throughout the purification process (Fig. 2a,b), eluting as a complex at 12.6 ml. Given that the Hcp3 hexamer alone elutes at 12.8 ml (ref. ^35^) (Fig. 2a), this modest shift suggests that Hcp3 binds Tce1 in its hexameric form, with the presence of the effector accounting for the slightly earlier elution, indicative of a heavier particle.

**Extended Data Fig. 2:**
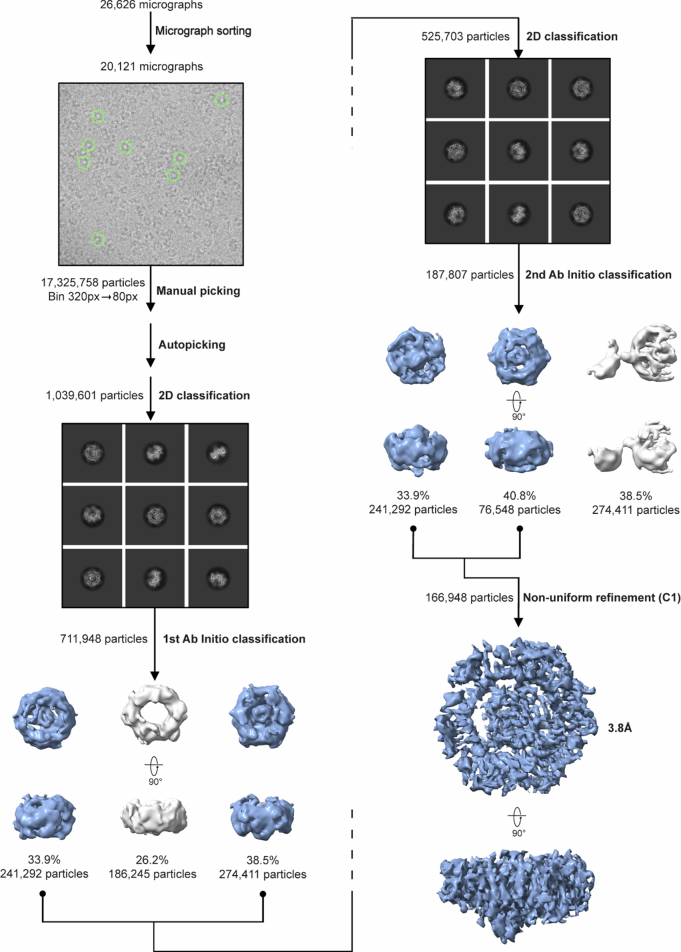
Cryo-EM processing pipeline of the Hcp3-Tce1 complex. The data processing produced an electron density map at an overall resolution of 3.8 Å.

**Extended Data Fig. 3:**
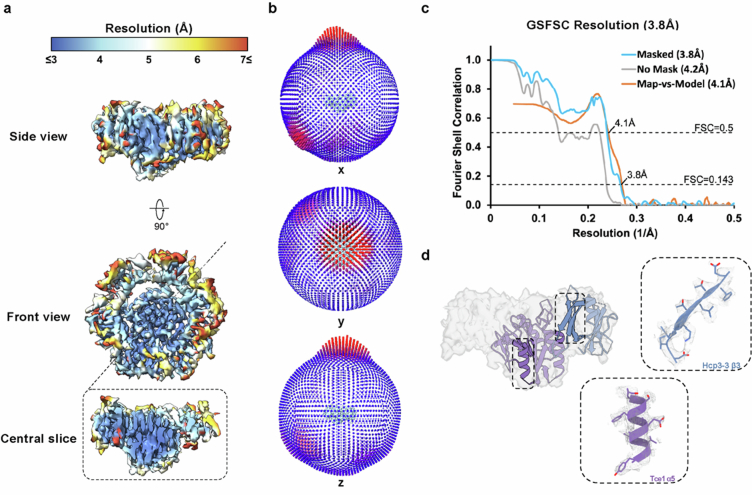
Resolution analysis of the Hcp3-Tce1 complex. **a** Local resolution estimation of the Hcp3-Tce1 map obtained using ChimeraX. The colour code indicates that the local resolutions across the map range predominantly at values below 4 Å. **b** Euler angle distributions for the overall cryo-EM map dataset. **c** Gold Standard Fourier Shell Correlation (GSFSC) for the final map, and map-vs-model validation using the full map and the experimental model. **d** The Hcp3-Tce1 model within the electron density map, Tce1 is displayed alongside one of the Hcp3 monomers. A more detailed fitting of the secondary structures along with residue side chains from Tce1 α1 (residues 130–140) and Hcp3-C β3 (residues 57–62).

Upon purification, the assembly and homogeneity of the complex were assessed by negative stain EM. After confirming the presence of intact, monodisperse complexes, the sample was vitrified on thin carbon layer grids and imaged by cryo-EM (Supplementary Table 1). After subsequent beam-induced motion and contrast transfer function (CTF) correction, particles were extracted from the collected micrographs and rounds of two-dimensional (2D) classification were performed. From this, a dataset of ~1 million particles was acquired, yielding high-resolution 2D class averages that display a clear ring structure with additional density observable within the central lumen only when Tce1 is present (Fig. 2c and Extended Data Fig. 2). From the side views, we could also infer that a portion of the effector density protrudes out of the ring. The high-resolution 2D classes were subjected to iterative rounds of ab initio classification followed by non-uniform refinement with C1 symmetry applied. The final map achieved an overall resolution of 3.8 Å, with local resolution extending to sub-3.4 Å across the complex (Extended Data Figs. 2 and 3).

**Fig. 3:**
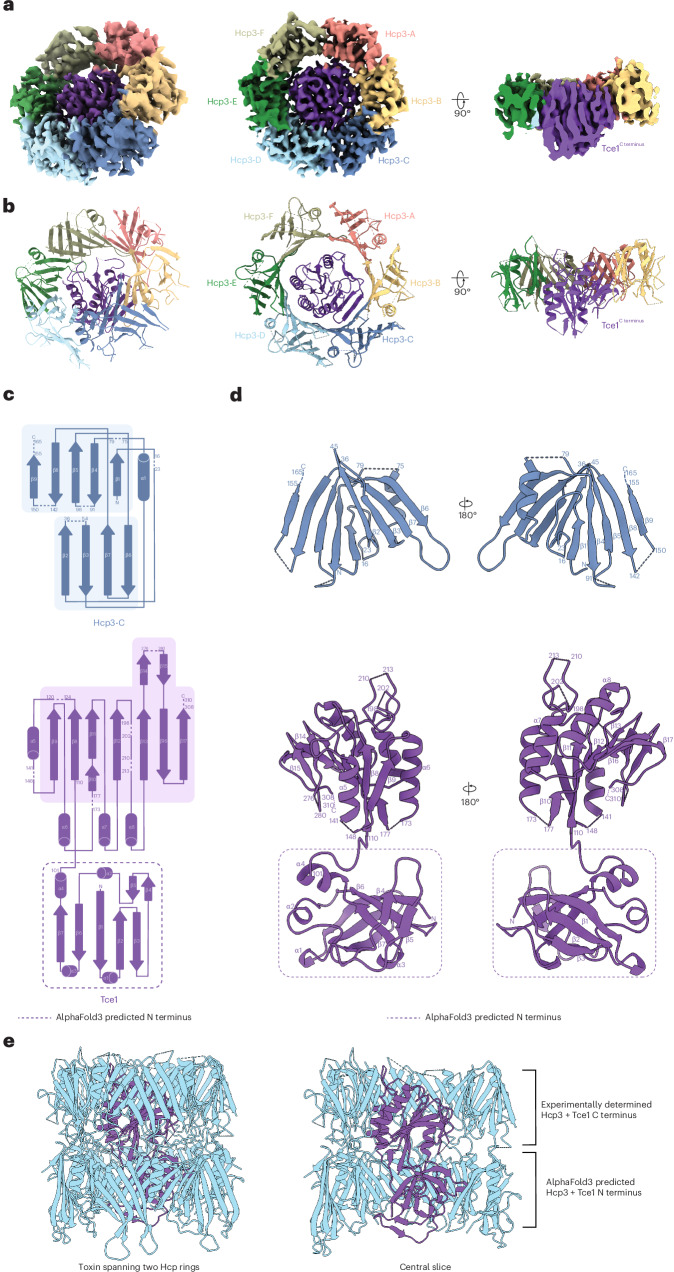
Tce1 binding to the Hcp3 hexameric ring. **a**, An overview of the tilted, end and side view of the electron density map for the Hcp3–Tce1^C terminus^ complex. Each chain is labelled and coloured to represent the individual proteins. **b**, A cartoon representation of the tilted, end and side view of the model of the Hcp3–Tse1^C terminus^ complex. **c**, Topology secondary structure diagrams of Hcp3 and the full-length Tce1 with individual β sheets highlighted. The AlphaFold3 predicted N terminus of Tce1 is outlined to differentiate it from the experimentally derived model. **d**, A cartoon representation of a Hcp3 monomer and Tce1 with a 180° rotated view. The AlphaFold3 predicted N terminus is outlined corresponding to **c** and the linker has been added to visualize the relative positions of the two domains. **e**, AlphaFold3 prediction demonstrating how two Hcp3 rings can accommodate the full-length Tce1. The fitting of the C terminus within the end ring is from the experimentally derived model.

The densities corresponding to the Hcp3 ring and Tce1 C terminus were identified in the map and used to build an atomic model of the entire Hcp3 ring in complex with the C-terminal domain (CTD) of Tce1 (Fig. 3a,b). The model of the Hcp3 ring displays six repeating units in a homohexameric conformation with each Hcp3 monomer made up of nine β-strands (β1–β9) forming two interfacing β sheets, alongside a single α helix (Fig. 3c,d). Hcp3 monomer–monomer interactions are stabilized through hydrogen bonds between α1 and β6 of one monomer with β2 and β9 of its neighbour, with specific interacting residues listed in Supplementary Table 2. These residues are repeated across all Hcp3 protomers in the hexameric ring. Although full-length Tce1 was used for co-purification, only the CTD of Tce1 carrying the putative cysteine protease catalytic site (amino acid 110–310) is structurally resolved in the cryo-EM reconstruction and comprises ten β strands (β9–β18) forming one central β sheet, surrounded by four α helices (α5–α8). The Tce1 CTD is found within the Hcp3 hexamer and the N-terminal domain (NTD) is expected to protrude outside of the hexamer lumen, where the flexibility of the exposed region results in low signal-to-noise during image averaging and prevents density reconstruction for this domain. As the cryo-EM density for Tce1 begins at Lys110, we modelled the full-length protein using AlphaFold3^37^ to predict the structure of the NTD comprising residues 1–109 (Fig. 3d). From this, we observe that the NTD possesses a β-sandwich domain arrangement (β1–β8) alongside four short α helices (α1–α4) and a short linker sequence (residues 106–109) that connects the two domains. We measured the Hcp3 ring to have an outer diameter of 85 Å and an inner diameter of 45 Å. Based on these dimensions, the lumen can accommodate only the CTD of Tce1, with a portion of it protruding from the ring. This indicates that the NTD of Tce1 would extend further outward from the ring and into an adjacent Hcp3 hexamer (Fig. 3e). Overall, our data strongly support a model in which two stacked Hcp3 rings entirely encapsulate full-length Tce1, with the CTDs and NTDs each positioned within the lumen of a ring (Fig. 3e).

### Tce1 interacts with inner-surface residues of the Hcp3 ring

**Fig. 4:**
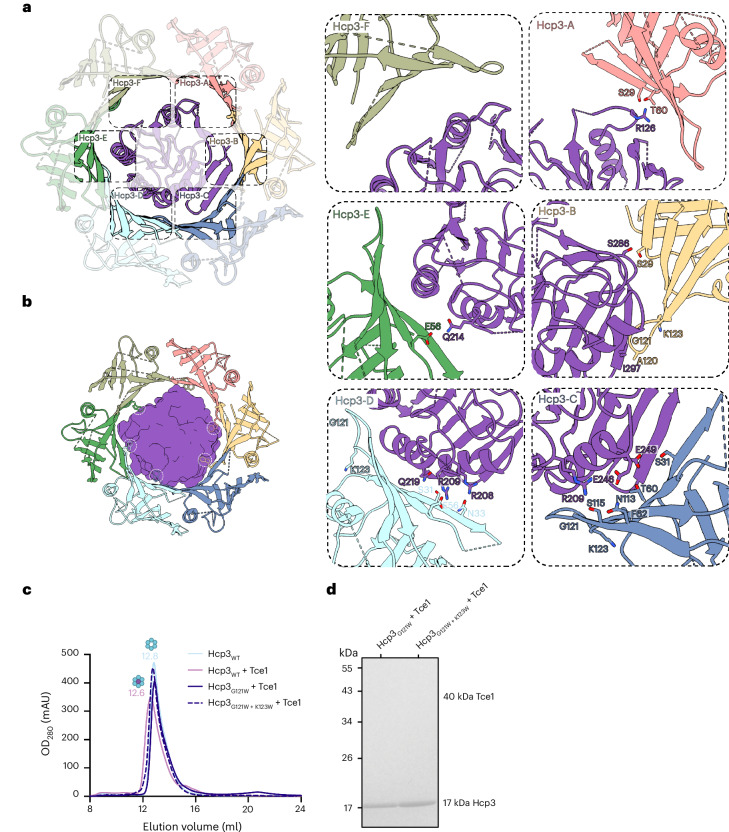
Structural features involved in the interaction between the Hcp3 hexamer and Tce1. **a**, Zoom in of the interfaces between Tce1 with each Hcp3 monomer. All interacting residues forming hydrogen/disulfide bonds, salt bridges or covalent links have their side chains displayed and are labelled. **b**, A cartoon representation of the Hcp3 ring with a surface representation of Tce1. The side chain of the G121W point mutation in each Hcp3 monomer is circled to highlight the steric clash introduced between these Hcp3 residues and Tce1. **c**, A chromatograph of the Hcp3_G121W_ + Tce1 and Hcp3_G121W+K123W_ + Tce1 co-purifications presented alongside the Hcp3_WT_, Hcp3_WT_ + Tce1 from Fig. 2a to visualize the shift in elution volumes between the WT and mutant complexes. **d**, SDS–PAGE gel of the peak fractions from the chromatograph in **c** stained with Coomassie blue. Co-purification Hcp3_G121W_ + Tce1 and Hcp3_G121W+K123W_ + Tce1 were performed independently in triplicate. Source data

**Extended Data Fig. 4:**
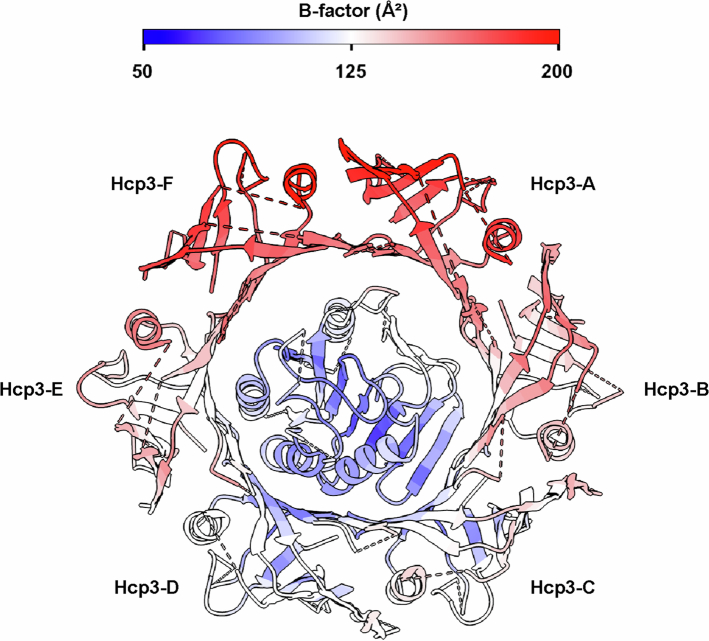
B-factor distribution across the Hcp3-Tce1 complex. Individual Hcp3 monomers are labelled and B-factor colour coding shown with a corresponding key.

With Tce1 existing as a monomer within the Hcp3 hexameric ring, its interface with each protomer is expected to differ, consistent with our structure, in which each Hcp3 molecule displays a different degree of interaction with Tce1 (Fig. 4a, Extended Data Fig. 4 and Supplementary Table 3). Key interacting residues form hydrogen bonds or salt bridges (Supplementary Tables 2 and 3), resulting in Tce1 situating asymmetrically within the Hcp3 lumen. Despite this overall asymmetry, we note that a number of Hcp3 residues are repeatedly involved in forming contacts with Tce1 across multiple protomers, namely S29, S31, E56 and T60, all forming hydrogen bonds with Tce1 across more than one protomer. We arbitrarily numbered Hcp3 protomers A through F and found that B, C and D all interface with Tce1 via residues G121 and K123 (Fig. 4a,b). These residues exist in a loop region between β6 and β7 that extends into the lumen of the assembled Hcp3 ring. This observation suggests that G121 and K123 are optimally positioned to validate the assembly revealed by our cryo-EM structure, where Tce1 resides within the Hcp3 lumen. Accordingly, we generated mutations in these interfacing residues to sterically modify the Hcp3 inner surface. Both G121W and K123W substitutions were generated to introduce, via the bulky nature of the tryptophan, steric hindrance within the Hcp ring lumen and restrict access to Tce1 (Fig. 4c). We compared the gel filtration chromatography profile of Tce1 co-purified with Hcp3_G121W_ and Hcp3_G121W+K123W_ with our previous co-purification using the native Hcp3_WT_ (Fig. 4d). In support of our structural data, we observe that in the presence of Tce1, both Hcp3_G121W_ and Hcp3_G121W+K123W_ elute at the same retention volume of Hcp3_WT_ alone, indicating that they do not interact with Tce1 (Fig. 4d). This observation is supported by SDS–PAGE analysis of the elution fractions, where only a band corresponding to Hcp3 is detected (Fig. 4e). Together, these results validate our three-dimensional (3D) structure of the Hcp3–Tce1 complex and demonstrate that, contrary to our initial expectation of broadly variable interfaces, Tce1 is asymmetrically positioned within the Hcp3 ring but repeatedly engages a defined set of Hcp interaction ‘hotspot’ residues. This finding indicates that Hcp–effector recognition arises from a combination of conserved contact sites and effector-specific interactions, rather than from stochastic asymmetry among Hcp protomers.

### Hcp3 sequentially wraps around Tce1

**Fig. 5:**
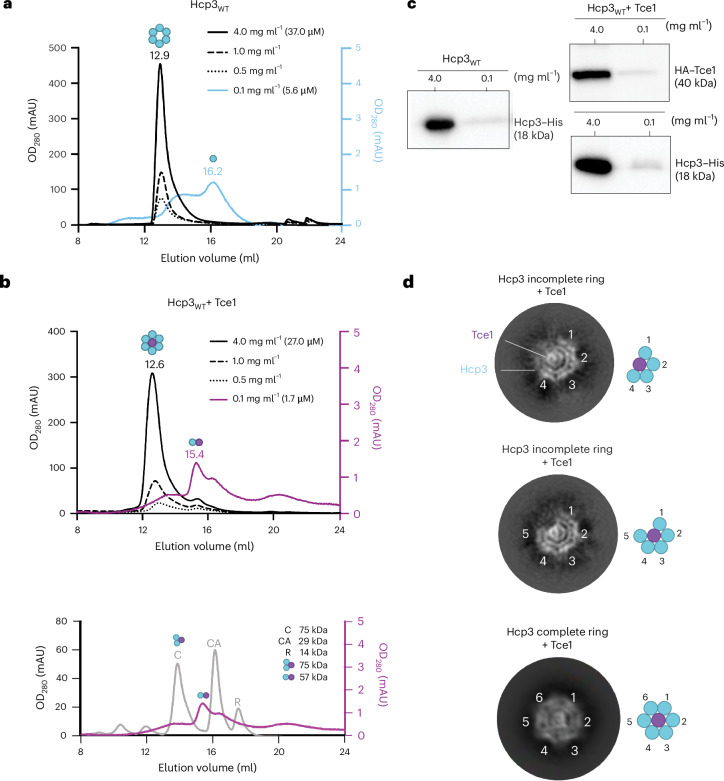
Assembly mechanism of the Hcp3–Tce1 complex. **a**, A chromatograph of Hcp3_WT_ purifications at 4.0, 1.0, 0.5 and 0.1 mg ml^−1^ with schematics representing the oligomeric state of Hcp3 at both retention volumes. The molarity of the Hcp3_WT_ hexamer at 4.0 mg ml^−1^ and the Hcp3 monomer at 0.1 mg ml^−1^ were calculated to be 37.0 μM and 5.6 μM, respectively. **b**, Top: a chromatograph of Hcp3_WT_ + Tce1 co-purifications at 4.0, 1.0, 0.5 and 0.1 mg ml^−1^ with schematics representing the oligomeric states of Hcp interacting with Tce1 at both retention volumes. The molarity of the Hcp3_WT_ hexamer in complex with Tce1 at 4.0 mg ml^−1^ and the Hcp3_WT_ monomer in complex with Tce1 at 0.1 mg ml^−1^ were calculated to be 27.0 μM and 1.7 μM, respectively. Bottom: Hcp3_WT_ + Tce1 co-purification at 0.1 mg ml^−1^ plotted overlaying a chromatograph of molecular weight standards for the Superdex S200 column to validate that the 15.4 ml peak corresponds to the molecular weight of a 1:1 Hcp3–Tce1 interaction. C, conalbumin; CA, carbonic anhydrase; R, ribonuclease A. **c**, The anti-his western blot from the peak fractions of the 4.0 mg ml^−1^ (6:1 Hcp3–Tce1 ratio) and 0.1 mg ml^−1^ (1:1 Hcp3–Tce1 ratio) purifications for Hcp3_WT_ and the anti-his and anti-HA western blots from the peak fractions of the 4.0 mg ml^−1^ and 0.1 mg ml^−1^ purifications for Hcp3_WT_ + Tce1. **d**, The 2D classes capturing the sequential assembly states of the Hcp3 ring in complex with Tce1 alongside a colour-coded representation of the proteins present in the 2D classes. Western blots were performed on samples from three independent purifications, yielding similar results. Source data

The asymmetric interaction between Tce1 and individual Hcp3 protomers suggests an assembly mechanism in which Tce1 first interacts with a single Hcp3 monomer followed by the rest of the ring assembling around it. To test this, we assessed the solution state behaviour of the Hcp3 ring at different protein concentrations. We purified Hcp3_WT_ at concentrations of 4.0 mg ml^−1^ (37 µM), 1.0 mg ml^−1^, 0.5 mg ml^−1^ and 0.1 mg ml^−1^ (5.6 µM) using affinity chromatography followed by gel filtration. At the higher concentrations, Hcp3_WT_ elutes at 12.9 ml (Fig. 5a), consistent with the previously observed retention volume expected for a hexamer (Fig. 2a). However, at 0.1 mg ml^−1^, the retention volume increases to 16.2 ml, indicating a substantial reduction in molecular weight consistent with monomeric Hcp3 (Fig. 5a). These results demonstrate that Hcp3 exists as a monomer at low concentrations and as a hexamer at higher concentrations, suggesting that ring formation is concentration dependent. Since Hcp3 elutes as a hexamer at concentrations of 0.5 mg ml^−1^ and above, the threshold concentration for hexamer assembly appears to lie between 0.1 mg ml^−1^ and 0.5 mg ml^−1^.

Next, we examined how Tce1 interacts with Hcp3 under conditions that favour incomplete ring formation. We co-purified Tce1 with Hcp3_WT_ at concentrations of 4.0 mg ml^−1^ (27 µM), 1.0 mg ml^−1^, 0.5 mg ml^−1^ and 0.1 mg ml^−1^ (1.7 µM), following the same protocol as for Hcp3_WT_ alone. The chromatographs for the 4.0, 1.0 and 0.5 mg ml^−1^ purifications depict the characteristic peak at 12.6 ml, corresponding to the Tce1-filled hexameric ring (Fig. 5b). At 0.1 mg ml^−1^, consistent with the behaviour of Hcp3_WT_ alone, the retention volume increases, but in this case to 15.4 ml, which is less than the 16.2 ml observed for monomeric Hcp3 alone. This shift probably reflects the addition of Tce1 (~39 kDa) to a monomeric or partially assembled Hcp3 species (Fig. 5b). Western blot analysis confirmed that both Tce1 and Hcp3 are present in the 15.4-ml elution fraction, demonstrating that Tce1 can interact with non-hexameric Hcp3 (Fig. 5c).

To strengthen these findings, we re-analysed the 2D classifications from the original cryo-EM dataset used to solve the Hcp3–Tce1 structure. We identified distinct 2D classes corresponding to intermediate stages of ring assembly in which Tce1 is only partially enclosed (Fig. 5d). Altogether, these results support a model in which Hcp3 ring formation is a sequential process rather than a discrete transition from monomer to hexamer. Furthermore, they reveal that Tce1 initially binds to a small number of Hcp3 monomers, with the rest of the ring assembling around the effector, even though not all subunits directly interact with it.

### Tce1 and Tce2 are putative cysteine proteases

**Fig. 6:**
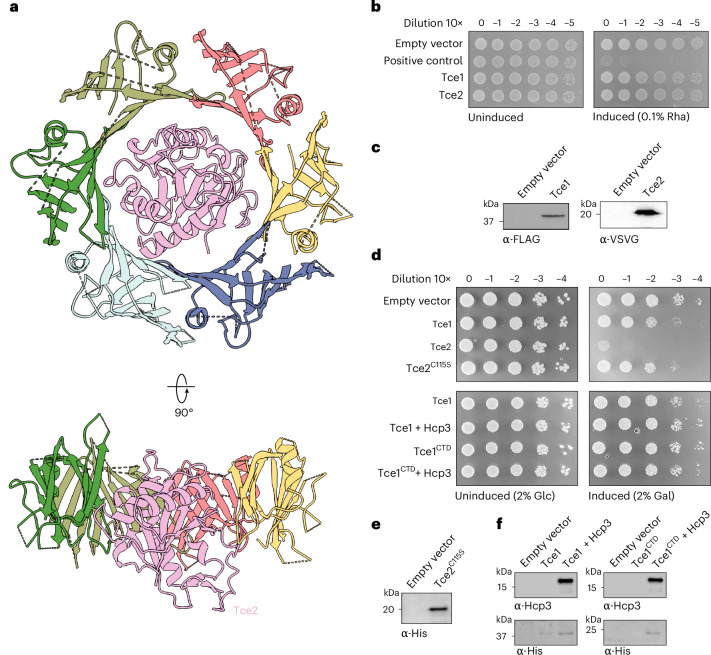
Tce2 is a cysteine protease with antifungal activity, which is also loaded into the Hcp3 lumen for toxin delivery. **a**, The predicted localization of the Tce2 AlphaFold3 structure within the experimentally derived Hcp3 ring, aligned to the experimentally derived Tce1 within the ring. **b**, The viability of *E. coli* harbouring plasmids encoding Tce1 or Tce2. The positive control is a known antibacterial toxin Tse6. Growth was performed in the absence of inducer, or in the presence of inducer (0.1% L-rhamnose (Rha)). **c**, Western blot analysis of liquid cultures of *E. coli* expressing Tce1 or Tce2. **d**, Viability of *S. cerevisiae* BY4742 harbouring plasmids encoding Tce1, Tce2 or a Tce2 catalytic variant Tce2^C115S^. Viability of *S. cerevisiae* co-expressing Tce1 and Hcp3 or the Tce1^CTD^ with Hcp3 was also evaluated. Plates either contained 2% glucose (Glc) for repression of expression or 2% galactose (Gal) for induction. **e**,**f**, Western blot analysis of Tce2 and catalytic mutant Tce2^C115S^ (**e**) and Tce1 or Tce1^CTD^ with and without Hcp3 co-expression in *S. cerevisiae* BY4742 (**f**). All experiments were performed with two independent biological replicates and two technical replicates, yielding similar results. Source data

**Extended Data Fig. 5:**
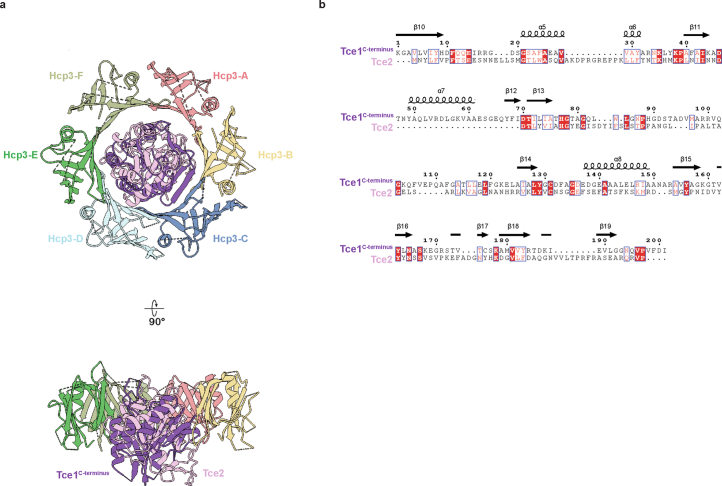
Structural and sequence comparison of Tce1 and Tce2. **a** Structural alignment of Tce2 with our experimentally derived Hcp3-Tce1^C-terminal^ structure, where the AF3 structure of Hcp3-Tce2 was used to aligned Tce2 within the Hcp3 lumen. **b** Sequence alignment of Tce1^C-terminus^ and Tce2 performed using ClustalW. The secondary structures of the Tce1^C-terminus^ are depicted above the alignment.

**Extended Data Fig. 6:**
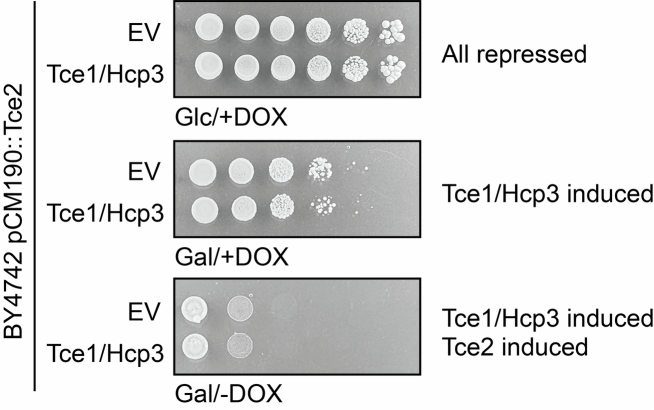
Viability of S. cerevisiae co-expressing Tce1, Tce2 and Hcp3 in *S. cerevisiae* BY4742. Plates either contained glucose and doxycycline for repression of both the Tce1/Hcp and Tce2, galactose and doxycycline for expression of Tce1/Hcp3 and repression of Tce2, or galactose with no doxycycline to express Tce1, Tce2 and Hcp3.

**Extended Data Fig. 7:**
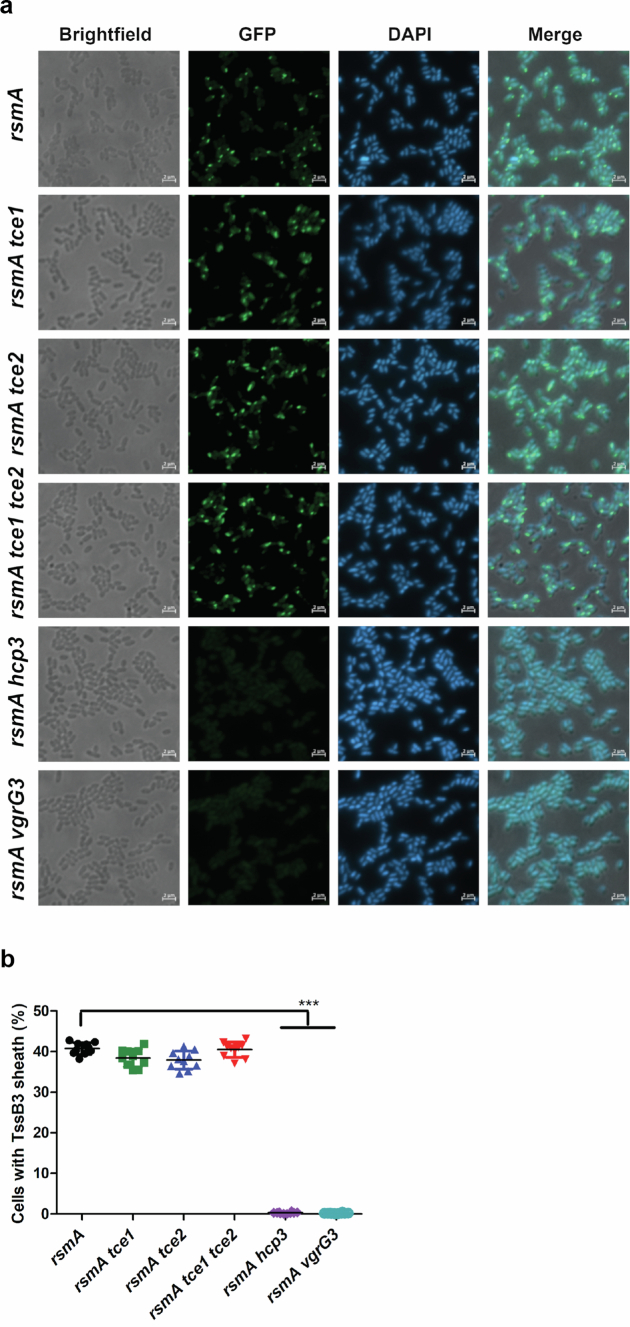
Fluorescence imaging and quantification of TssB3-sfGFP sheaths in *P. aeruginosa* T6SS mutants. **a** Representative fluorescence microscopy images of *P. aeruginosa* rsmA expressing TssB3-sfGFP with mutations in *tce1*, *tce2*, *tce1tce2*, *hcp3* and *vgrG3* respectively. Scale bars represent 2 µm. Channels shown are brightfield, GFP, DAPI and merge. **b** Quantification of the sheath shown in ‘**a**’. The results are the means of percentage of TssB3-sfGFP sheath containing cells ± the standard deviations from three biological replicate cultures and are representative of at least three independent trials. Mean values were obtained from analyses of 15000 cells per replicate for each strain. Asterisks indicate that the values are significantly different between strains compared (***, *P* < 0.001; one-way ANOVA with Tukey’s Multiple Comparison post test).

Based on our experimentally derived Hcp3–Tce1 complex and using AlphaFold3^37^, we predict that similar to Tce1, Tce2 is positioned within the lumen of the Hcp3 ring and engages the surrounding Hcp3 protomers asymmetrically, probably spanning two stacked Hcp3 rings (Fig. 6a). Tce1 and Tce2 both possess central β sheets encircled by α helices (Fig. 1a,c). Both β sheets align, localizing Tce2 to the same region of the Hcp3 lumen as seen with Tce1 (Extended Data Fig. 5a,b). Tce2 sits in the lower half of the Hcp3 ring, partially protruding out from the underside of the ring (Fig. 6a). Interacting residues between Hcp3 and Tce2 were identified using PDBePISA analysis of the AlphaFold3 model of the Hcp3–Tce2 complex (Supplementary Table 4). Several Hcp3 residues that form contacts with Tce1—S29, S31, E56, T60, N113 and S115—are also predicted to interact with Tce2, indicating that these conserved Hcp3 residues are reused across distinct effector interfaces (Supplementary Table 4). In addition to this shared core, Hcp3 forms further hydrogen bonds or salt bridges with each toxin through effector-specific residues. We then revisited our previous suggestion that like most T6SS effectors, Tce1 and Tce2 possess antibacterial activity^10^. However, our data showed that this is probably not the case because heterologous expression of these enzymes in *E. coli* did not markedly affect growth (Fig. 6b,c). This is also supported by the observation that no obvious immunity protein-encoding^39–42^ genes are found in the vicinity of the effector-encoding genes (Extended Data Fig. 1). We investigated potential anti-eukaryotic toxicity^9,43^ by expressing Tce1 and Tce2 in *Saccharomyces cerevisiae* and observed that Tce2 impairs yeast growth. This toxicity is alleviated by mutating the predicted active site of the putative cysteine protease, confirming its functional relevance (Fig. 6d). By contrast, Tce1 did not measurably affect yeast growth. Western blot analysis revealed detectable expression of Tce2, but only small amounts of Tce1, suggesting Tce1 may be unstable in this context (Fig. 6e,f). To mitigate this instability, we co-expressed Tce1 with Hcp3, and although Tce1 could now be detected to a greater extent, no impact was observed on yeast growth. A possible hypothesis for the lack of toxicity is that the extended N terminus of Tce1, as compared with Tce2, may interfere with putative cysteine protease activity. We produced a N-terminally truncated version of Tce1 and expressed it in yeast in the presence/absence of Hcp3, but in all cases no toxicity could be observed (Fig. 6d,f). Finally, to determine whether Tce1 toxicity depends on the presence of Tce2, we co-expressed Tce1, Tce2 and Hcp3. However, this did not enhance or alter the toxicity observed with Tce2 and Hcp3 alone (Extended Data Fig. 6), further supporting that Tce1 is non-toxic in yeast under these conditions. Overall, our data show that the Hcp3-associated effector Tce2 possesses antifungal properties. This previously undescribed antifungal activity for an effector associated with the H3-T6SS suggests this system might possess anti-eukaryotic activity. As reported previously, H3-T6SS-dependent secretion could not be monitored in vitro owing to the molecular trigger responsible for H3-T6SS firing remaining unknown^35^. Nevertheless, we assessed whether Tce1 and Tce2 are genuine effectors rather than H3-T6SS assembly factors by monitoring H3-T6SS sheath assembly. We used a tagged TssB3-sfGFP^18^ and monitored sheath assembly in different *P. aeruginosa* backgrounds, including *tce1*, *tce2*, *tce1/tce2*, *vgrG3* and *hcp3* mutants, using fluorescence microscopy. We observed that when the H3-T6SS is non-functional, that is, in the *vgrG3* and *hcp3* mutant strains, no sheaths could be observed, whereas in the *tce1*, *tce2* or *tce1/tce2* deletion backgrounds the numbers of sheaths formed is like wild-type (WT) (Extended Data Fig. 7).

## Discussion

**Extended Data Fig. 8:**
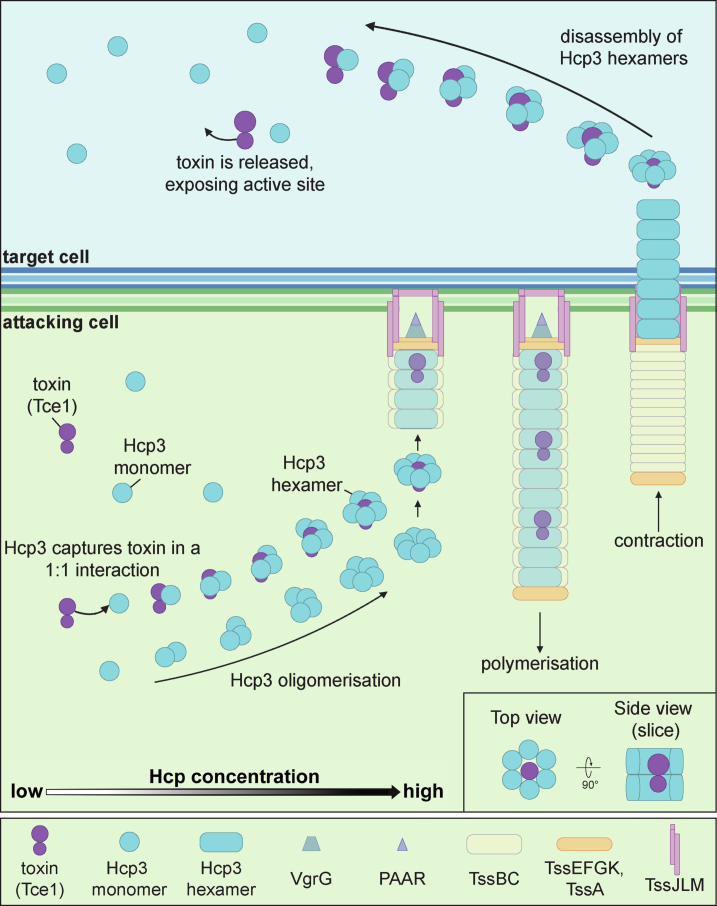
Proposed mechanism of concentration driven Hcp3 ring assembly and disassembly in vivo. The mechanism is instigated in the attacking cell where Hcp3 captures the Tce1 toxin in a 1:1 interaction before sequential concentration driven assembly into a hexameric ring. In this mechanism, Tce1 is captured to different degrees by each Hcp3 protomer as oligomerisation occurs, resulting in asymmetric loading within the ring. Hcp3 also oligomerises into hexameric rings in the absence of a bound toxin. The rings then polymerise to form the tube of the T6SS tail complex which is injected into the target cell upon T6SS contraction. In the target cell, the low Hcp3 concentration promotes ring disassembly, releasing Tce1 and freeing its active site. The pre-firing events described here: Hcp3-Tce1 binding and concentration driven oligomerisation into hexameric rings are supported by our experimental data. The post-firing concentration driven ring disassembly and Tce1 release in the target cell are hypothetical and inferred from our proposed model of Hcp3 disassembly in low concentrations.

T6SS effectors can be delivered either as specialized effectors, in which toxic domains are part of VgrG, PAAR or Hcp proteins, or as cargo effectors that are associated with VgrG or PAAR proteins, or are loaded into the lumen of the Hcp tube. While specialized effectors rely on their fusion to core T6SS components for delivery, cargo effectors require distinct loading mechanisms. Multiple mechanisms have been proposed for how effectors are selected, loaded and delivered by the T6SS. It was proposed that large effectors associate with the T6SS tip, that is, VgrG or PAAR, whereas smaller effectors insert into the Hcp lumen such as the Tse2 cargo effector^31^. Despite many Hcp structures having been reported^15,16,44–49^, no studies presented the high-resolution structure of an Hcp–effector complex. Here, we reveal the cryo-EM structure of the *P. aeruginosa* Hcp3–Tce1 complex at 3.8-Å resolution and show how Tce1 is wrapped within the lumen of a homohexameric Hcp3 ring. One key finding is the sequential nature of the process during which Tce1 initially interacts with monomeric Hcp3 through specific contacts, notably the formation of specific hydrogen bonds and salt bridges (Supplementary Table 2). Our data indicate that this initial 1:1 Hcp3–Tce1 interaction can nucleate hexamer formation by stabilizing an early Hcp3–Hcp3 assembly intermediate. Additional Hcp3 protomers are then recruited and oligomerize around the effector, establishing asymmetric interactions that culminate in a fully assembled and effector-loaded Hcp3 hexamer (Supplementary Movie 1). Probably, effector binding promotes and stabilizes hexamer formation, rather than being strictly required for Hcp3 hexamer assembly (Extended Data Fig. 8). In this process, S29, S31, E56 and T60 serve as key hydrogen bond-forming residues, interacting with Tce1 across multiple Hcp3 protomers. Additional hydrogen bonds form between each of the five interacting Hcp3 protomers and Tce1, facilitating capture of the effector. The varying number of interacting residues between Hcp3 protomers and Tce1 can be visualized through the B-factor variation across the Hcp3–Tce1 structure, namely between each protomer and the interacting region of the effector. Here, changes in the B factor coincide with a greater number of hydrogen bonds between Tce1 and certain Hcp3 protomers (Extended Data Fig. 4). Although our conclusions are derived from Tce1, the underlying principles—namely asymmetric engagement and the combination of conserved and effector-specific interfaces—may be broadly applicable across T6SS cargo effectors. Consistent with this, none of the Tce1 residues that contact Hcp3 are conserved in Tce2 based on sequence alignment and interface mapping via PDBePISA. By contrast, several Hcp3 residues are reused across both effectors, supporting a model in which conserved Hcp3 interaction hotspots accommodate structurally distinct effector surfaces. Together, these observations suggest that while Hcp3 engages different effectors through a shared core of conserved residues, effector-specific surface chemistry determines the pattern of asymmetric binding. A recent observation showing that Hcp proteins could form heterohexamer would also suggest that there is further flexibility in cargo loading within the T6SS^50^.

While it was shown that effectors are loaded into the lumen of Hcp rings^31^, information on Hcp–effector stoichiometry or the loading process remained inconclusive. Our data reveal that the loading occurs in a stepwise recruitment of Hcp protomers around the effector rather than insertion into a preformed ring (Extended Data Fig. 8). This assembly mechanism can, to some extent, be compared with the Tat system^51^, which translocates folded proteins across the cytoplasmic membrane. In this case, TatA monomers wrap around the substrate through oligomerization^52^. This mechanism helps accommodate proteins of varying sizes by recruiting more TatA protomers for large substrates. In the case of Hcp, modulation in the number of protomers in the ring is not possible owing to the size and structure of the Hcp rings being fixed and rigid. However, our work revealed that effectors can be enclosed by more than one Hcp ring, creating additional space to accommodate larger effectors than would be possible within the lumen of a single ring. In this case, the interaction between the Tce1 N terminus and the second Hcp3 ring is nonspecific and driven by spatial proximity rather than a defined binding interface. This secondary encapsulation reflects an inherent capacity of stacked Hcp rings to accommodate large and extended cargo.

Another implication of our findings is that for specialized Hcp effectors^53^, that is, Hcp proteins carrying a toxin domain, the ring cannot be formed by more than one Hcp-extended effector (Hcp-ET) due to the steric incompatibility of packing more than one effector in the Hcp lumen. Instead, the extended Hcp may bind to five other ‘regular’ Hcps that complete the ring around one Hcp-ET. It is also striking that each of the two effectors, Tce1 and Tce2, can be independently packed within Hcp3 rings. The difference in binding of Tce1 to Hcp3, compared with Tce2, may be explained by their genomic context. While Tce2 is encoded within the H3-T6SS cluster and co-transcribed with *hcp3* (Extended Data Fig. 1), *tce1* is located distantly. It is therefore plausible that Tce1 has evolved a higher affinity for Hcp3 to compensate for its genomic separation^35^. Alternatively, it may suggest a mechanism that drives loading preference, where effectors with higher affinity are loaded first. This strategy is used for fimbrial assembly in which fimbriae subunits that need to be recruited first display higher affinity for the outer membrane usher protein compared with subunits that should be assembled subsequently^54^. In the T6SS, such mechanism would predict that there are more Tce1 molecules loaded into the H3-T6SS apparatus than there are of Tce2, thus introducing a regulatory control in terms of T6SS impact on target cells. Furthermore, our observation that ring assembly is concentration dependent suggests that intracellular levels of Hcp3 and its cargo might regulate the formation of secretion-competent complexes, adding a layer of control to T6SS function. It was also shown in *Vibrio cholerae* that Hcp accumulation can drive T6SS gene repression through interaction with the VasH regulator^55^. We could thus predict that accumulation of free Hcp in the absence of effectors may result in T6SS downregulation. This would align with the onboard checking mechanism described for VgrG–PAAR in *V. cholerae* and T6SS assembly failure in the absence of effectors^56^. Although absolute intracellular concentrations of Hcp3 and its cognate effectors in vivo may not be directly represented by the concentrations used in vitro, given the influence of additional factors such as local protein concentrations and spatial organization, these experiments have identified the fundamental intrinsic property of Hcp3 to undergo concentration-dependent assembly into hexameric rings.

Tce2 is a probable cysteine protease that negatively impacts yeast growth. When compared with Tce2, Tce1 exhibits the same conserved cysteine and histidine residues that align with the catalytic dyad of the well-characterized *C. difficile* toxin TcdB^57^ (Fig. 1b). However, unlike Tce2, Tce1 does not cause pronouced growth inhibition in yeast. This may imply that Tce1 protease activity is tightly regulated and potentially activated only upon delivery into a physiologically relevant target cell. Hcp3 is part of the *P. aeruginosa* H3-T6SS, which was shown to be involved mainly in anti-eukaryotic activity^58^. Intriguingly, and in contrast with the H1-T6SS and H2-T6SS, for which numerous effectors have been described, effectors associated with H3-T6SS are scarce^33,59^. Two have been associated with VgrG3, TepB^60^ and TseF^61^, both having an elusive role in common goods acquisition^62^ and/or injected in eukaryotic cells as is the case for TepB^60^. Our findings that Tce2, and possibly Tce1, are anti-eukaryotic effectors strengthen this concept. The molecular target(s) for Tce2 and Tce1 are unknown, but the evolutionary diversity of cysteine protease effectors makes potential protease substrates challenging to predict. For example, Cpe1 is a papain-like cysteine protease that targets type II DNA topoisomerases GyrB and ParE^63^, whereas the *Shigella flexneri* OspB cleaves the metabolic regulator TORC1 in yeast and mammalian cells^64^. Although sharing a conserved catalytic core, differences in accessory domains and activation motifs probably reflect adaptation to specific host targets and regulatory environments. Although these results suggest that Tce2, and possibly Tce1, possess interkingdom activity, identifying the cellular targets of Tce2 or potential auxiliary factors required for Tce1 activity will require further investigation.

The observation that the Hcp3 ring can be loaded with either Tce1 or Tce2 supports the concept of the injection of a cocktail of effectors by a single T6SS. How many Hcp3 rings contain Tce1 compared with Tce2 remains to be understood and some of the rings might need to remain empty to fulfil steric requirements within the T6SS sheath. Together, a fully loaded apparatus constitutes a very powerful effector/toxin injection device, and one may consider that empty Hcp rings could also have potential toxic activity as proposed for Hcp in *Acidovorax citrulli*^65^. Furthermore, our observation that Hcp proteins selectively recognize their cognate effectors can be extended to T6SSs across distantly related bacteria, as exemplified by *Bacteroides* T6SSs, which encode multiple Hcp homologues within a single system and form heterohexameric assemblies while still maintaining effector specificity^63^.

In summary, by defining the molecular basis of T6SS effector loading and establishing an unprecedent link between effector recognition, sequential ring assembly and concentration-dependent oligomerization, this work provides a framework for understanding how bacteria deploy T6SS effectors to modulate intercellular interactions.

## Methods

### Bacterial cultures and Hcp3–Tce1 expression and purification

The bacterial strains and plasmids used in this study are listed in Supplementary Table 5. Bacteria were grown in lysogeny broth (LB) (Miller) or on LB agar (Miller plates) at 37 °C unless stated otherwise. The genes encoding Hcp3–His and HA–Tce1 variants were cloned into pACYCDuet-1 and pET22b vectors, respectively, and transformed into BL21 cells. Cells were grown in 1 l LB with appropriate antibiotics and protein expression was induced at an OD_600_ of 0.6 by the addition of 0.5 mM IPTG for 20 h at 20 °C. The cells were collected by centrifugation at 7,000*g* for 30 min at 4 °C. The pellet was resuspended in 20 mM HEPES (pH 7.5), 250 mM NaCl, 100 mg ml^−1^ lysozyme, 100 μl ml^−1^ Triton X-100 and 0.5 mM PMSF. Following lysis by sonication on ice, cellular debris were removed by centrifugation at 18,000*g* for 45 min. The supernatant was incubated with 1 ml of TALON metal affinity resin pre-equilibrated in buffer (20 mM HEPES and 250 mM NaCl) for 1 h. The resin was loaded onto an Econo-Pac gravity flow chromatography column and washed with 100 ml of buffer (20 mM HEPES, 250 mM NaCl and 7 mM imidazole) before elution with 10 ml buffer (20 mM HEPES, 250 mM NaCl and 500 mM imidazole). Elution buffer was added 2 ml at a time to be collected into 5× 2 ml fractions. Fractions containing protein were pooled into 3.5 kDa molecular weight cut off SnakeSkin dialysis tubing and dialysed in 20 mM HEPES pH 7.5 and 150 mM NaCl overnight at 4 °C. Following dialysis, the sample was concentrated using a centrifugal concentrator with a 3 kDa molecular weight cut off. SEC was performed on this sample using a Superdex 200 GL 10/300 column (GE Healthcare) pre-equilibrated in SEC buffer (20 mM HEPES pH 7.5 and 150 mM NaCl). The presence of both Hcp3–His and HA–Tce1 following SEC was analysed using Coomassie stain and western blot analysis.

### EM sample preparation and data collection

For the structural determination, the SEC-purified Hcp3–Tce1 complex was incubated with 0.1% glutaraldehyde crosslinking followed by another SEC purification. A 4 µl aliquot from the peak fraction, with the same retention volume as the uncrosslinked Hcp3–Tce1 SEC purification, at a concentration of 0.25 mg ml^−1^ was applied onto a glow-discharged Quantifoil R1.2/1.3 Cu 300 mesh holey grid (Agar Scientific) to ensure good particle density for reliable imaging and data processing. After 60 s, the excess sample was blotted for 4 s and blot force −2 under 95% relative humidity at 4 °C and plunge-frozen in liquid ethane using Vitrobot Mark IV (Thermo Fisher). The dataset was collected on FEI Titan Krios microscope operating at 300 kV at the UK National Electron Bio-Imaging Centre (eBIC). Imaging was automated using EPU software (FEI) on a Falcon 4i detector with a pixel size of 0.921 Å (130k magnification). A total of 26,626 movies were recorded, with a nominal defocus range of approximately −1.0 to −2.7 µm with a total dose of 40 e^−^ Å^−2^ over 4.89 s, corresponding to a dose rate of 7.44 e^−^ per pixel per second.

### Cryo-EM image processing and reconstruction

All image processing was performed with CryoSPARC v4.4.1^66^ unless stated otherwise. The movies were aligned using patch motion correction and CTF corrected through patch CTF estimation, then curated to select for optimum ice thickness, CTF estimation and resolution. From 20,121 selected micrographs, particles were picked and 2D classified to generate a template for automated particle picking. After the particle picking parameters were refined using a small number of micrographs, a total of 1,039,601 particles were auto picked from the entire dataset and extracted using a box size of 320 × 320 pixels with subsequent downsampling by Fourier cropping to 160 pixels. Several rounds of 2D classification were implemented to remove low quality particles, yielding a stack of 711,248 particles from which ab initio maps were generated. The maps from the best classes were selected and a secondary round of 2D classification followed by ab initio modelling was performed. The suitable classes underwent non-uniform refinement, producing a map with an estimated overall resolution of 3.8 Å (Extended Data Figs. 2 and 3). The local resolution of the map was estimated using CryoSPARC^66^ and visualized with ChimeraX^67^.

### Model building and refinement

An AlphaFold model of a single Hcp3 chain was used as the starting model. This model was rigid-body fitted into the density corresponding to one of the Hcp3 chains using Chimera. This process was repeated five more times, each time placing a new AlphaFold model with a unique chain label into unoccupied density, thereby constructing the hexameric ring of the Hcp3 protein. Similarly, an AlphaFold model of the Tce1 toxin was placed into the density using Chimera^67^. As density was available for only one of the two domains, the models of the NTD and CTD were separated and independently rigid-body fitted into the map. These fittings were visually using Chimera^67^ and assessed to determine which domain best corresponded to the observed density. The CTD model provided a better fit and was retained, while the NTD model was discarded (Extended Data Fig. 3d). An initial model of the Hcp3–toxin complex was thus constructed, comprising six Hcp3 chains (chain labels A–F) and one toxin chain (label G). Owing to anisotropy in the map, density at the periphery appeared fragmented or disconnected. To improve the fit of the model to the map, each chain was manually inspected in Coot and residues not supported by density were removed. The revised Hcp3–Tce1 model underwent several iterative rounds of real-space refinement using Phenix^68^. Model quality and refinement progress were monitored using Ramachandran plots and MolProbity^69^.

### Structure analysis and presentation

The visualization and analysis of the cryo-EM maps and atomic models were carried out using ChimeraX, PyMol (Molecular Graphics System, Schrödiner) and PDBePISA.

### Sequence analysis and presentation

The sequence alignments performed in this study were carried out using Clustal Omega, and all sequences were acquired from Pseudomonas.com. Analysis of the sequence alignments was performed with ESPript3.2.

### Strains, plasmids and growth conditions

Bacterial strains and plasmids used are listed in Supplementary Table 5. Bacterial cultures were grown in LB with antibiotics where applicable (trimethoprim 200 µg ml^−1^). Growth of yeast strains was accomplished with synthetic complete media lacking uracil or uracil and leucine (SC-Ura and SC-Ura Leu; BioShop). Yeast plasmids were first cloned in *E. coli* and subsequently introduced in yeast using the lithium acetate method^70^. Plasmids were induced by the addition of galactose or repressed with the addition of doxycycline where applicable.

### Plasmid construction and genomic mutagenesis

Plasmids were constructed using restriction cloning with Phusion DNA polymerase, restriction enzymes and T4 DNA ligase from New England Biolabs or with Gibson assembly (New England Biolabs). All primers were synthesized by Integrated DNA Technologies (Supplementary Table 6). Plasmids were confirmed through Plasmidsaurus sequencing.

### Purification of Hcp3 for antibody generation

Overnight cultures of BL21 (DE3) pLysS expressing Hcp3 were subcultured 1:100 into 1 l of LB and grown to mid-log phase. Cultures were induced by the addition of 0.5 mM isopropylthiogalactoside (IPTG) and grown for 18 h at 18 °C. Cultures were collected and resuspended in buffer A (50 mM Tris pH 7.5 and 500 mM NaCl) and lysed by sonication. Lysates were cleared by centrifugation at 36,000*g* for 45 min. The cleared lysate was loaded onto 1 ml Ni-NTA resin and washed with additions of buffer A with 10 mM imidazole followed by buffer A with 30 mM imidazole. Bound protein was eluted by the addition of buffer A containing 250 mM imidazole. The elution was concentrated and buffer exchanged using a PD-10 column (Cytiva).

### Spot plate assays

Spot plates were prepared by normalizing overnight cultures to an OD_600_ of 1 and making serial dilutions. Then 10 μl spots were plated and allowed to dry and bacterial plates were incubated for 24 h at 37 °C while yeast plates were incubated for 48 h and 30 °C, after which time plates were imaged.

### Western blotting

For overexpression of proteins in *E. coli*, overnight cultures were diluted 1:100 in 25 ml of LB. Cultures were grown to mid-exponential phase and induced by the addition of 0.1% L-rhamnose. After 2 h, cells were collected and resuspended to a final volume of 0.5 ml. For overexpression of proteins in yeast, overnight cultures grown in 2% raffinose were diluted 1:100 and grown in 2% raffinose until log phase. Cultures were induced by the addition of 2% galactose and grown for an additional 18 h, after which time cells were collected. Pellets were resuspended in 10 ml of lysis buffer (50 mM Tris pH 7.4 and 500 mM NaCl) and cells were lysed using an emulsiflex. Lysates were cleared by centrifugation and mixed 1:1 with loading dye and boiled for 10 min. Then 5 µl of sample was separated on a 12% Tris–glycine SDS–PAGE gel and subsequently transferred to nitrocellulose at 100 V for 30 min. Blots were probed with either anti-FLAG (1:5,000 Sigma), anti-VSV-G (1:5,000 Sigma), anti-His_6_ (1:5,000 GenScript) or anti-Hcp3 (1:1,000 GenScript). Secondary antibodies were 1:5,000 goat anti-mouse horseradish peroxidase (New England Biolabs) for anti-His_6_ blots or 1:10,000 goat anti-rabbit (New England Biolabs) for anti-VSV-G, anti-Hcp3 or anti-FLAG blots.

### Fluorescence microscopy

We generated mutants in *tce1*, *tce2*, *hcp3* and *vgrG3* in a *P. aeruginosa rsmA* strain expressing TssB3-sfGFP from the native *tssB3* locus^18^. Bacterial cultures for fluorescence microscopy were grown overnight in TSB at 37 °C with shaking at 200 rpm. Bacterial suspensions were then subcultured at an OD_600_ of 0.1 in 25 ml TSB and grown at 25 °C with shaking at 200 rpm for 24 h. Cells were visualized using a µ-Dish (35-mm, high glass bottom) made of a #1.5H glass coverslip (Ibidi). Then 1 µl of bacterial suspension in PBS containing 10 µg ml^−1^ DAPI was placed onto agarose slabs (0.5-cm thickness; 2% w/v in PBS), which were placed face down onto the glass-bottom window of the µ-Dish so that cells were sandwiched between the glass and the agarose slab. Samples were imaged using Axio Observer 7 (Zeiss) inverted widefield microscope equipped with a Plan-Apochromat 63×/1.40 NA oil DIC M27 objective (Zeiss) and an Orca Flash 4.0 camera (Hamamatsu). Images were acquired using 1 × 1 binning corresponding to a pixel size of 0.103 µm × 0.103 µm.

### Image analysis and presentation

Detection of fluorescent TssB3-sfGFP foci was performed on images using the local maxima detection algorithm implemented in FIJI^71^ with a threshold of 1,000 for DAPI and 2,300 for sfGFP. The number of cells with a TssB3-sfGFP foci was calculated and expressed as a percentage of the total number of cells in a field. A total of approximately 15,000 cells were counted for each strain per experiment. For presentation, generation of images was performed using the ZEN Blue software. For each strain, an image subset was created with scale bar and exported separately for each channel as well as merged as TIFF files. The images were compiled using GIMP 3.0.

### Reporting summary

Further information on research design is available in the Nature Portfolio Reporting Summary linked to this article.

## Supplementary information

Reporting Summary

Peer Review File

Supplementary Information Supplementary Tables 1–6.

Supplementary Video

## Source data

Source Data Fig. 1 Unprocessed western blots and/or gels.

Source Data Fig. 2 Unprocessed western blots and/or gels.

Source Data Fig. 4 Unprocessed western blots and/or gels.

Source Data Fig. 5 Unprocessed western blots and/or gels.

Source Data Fig. 6 Unprocessed western blots and/or gels.

## Data Availability

The atomic coordinates have been deposited at the Protein Data Bank with accession code 9QPE. The density map has been deposited at the Electron Microscopy Data Bank with accession code EMD-53274. Source data are provided with this paper.
