# Supplementary material for: Molecular basis of type VI secretion system effector loading
Source: Nat Microbiol. 2026 May 27;11(7):1982–94. doi: 10.1038/s41564-026-02363-x (PMC13323088; doi:10.1038/s41564-026-02363-x)
Supplement: Supplementary file 1 — Reporting Summary [file 41564_2026_2363_MOESM1_ESM.pdf]

nature portfolio

Corresponding author(s): Alain Filloux and Tiago R D Costa

Last updated by author(s): Apr 13, 2026

Reporting Summary

Nature Portfolio wishes to improve the reproducibility of the work that we publish. This form provides structure for consistency and transparency in reporting. For further information on Nature Portfolio policies, see our Editorial Policies and the Editorial Policy Checklist.

Statistics

For all statistical analyses, confirm that the following items are present in the figure legend, table legend, main text, or Methods section.

n/a	Confirmed	
☐	☒	The exact sample size (n) for each experimental group/condition, given as a discrete number and unit of measurement
☐	☒	A statement on whether measurements were taken from distinct samples or whether the same sample was measured repeatedly
☐	☒	The statistical test(s) used AND whether they are one- or two-sided<br>*Only common tests should be described solely by name; describe more complex techniques in the Methods section.*
☐	☒	A description of all covariates tested
☐	☒	A description of any assumptions or corrections, such as tests of normality and adjustment for multiple comparisons
☐	☒	A full description of the statistical parameters including central tendency (e.g. means) or other basic estimates (e.g. regression coefficient) AND variation (e.g. standard deviation) or associated estimates of uncertainty (e.g. confidence intervals)
☐	☒	For null hypothesis testing, the test statistic (e.g. F, t, r) with confidence intervals, effect sizes, degrees of freedom and P value noted<br>*Give P values as exact values whenever suitable.*
☒	☐	For Bayesian analysis, information on the choice of priors and Markov chain Monte Carlo settings
☒	☐	For hierarchical and complex designs, identification of the appropriate level for tests and full reporting of outcomes
☒	☐	Estimates of effect sizes (e.g. Cohen's d, Pearson's r), indicating how they were calculated

Our web collection on statistics for biologists contains articles on many of the points above.

Software and code

Policy information about availability of computer code

Data collection	All data collection software is described in the Methods section. Single particle data was acquired using EPU (Thermo Fisher). Protein prediction: AlphaFold3. Gel imaging: BioRad, ImageLab v3.0.1.14.
Data analysis	All data analysis software is described in the Methods section. Single particle data processing was carried out using cryoSPARC v. 4.4.1.

For manuscripts utilizing custom algorithms or software that are central to the research but not yet described in published literature, software must be made available to editors and reviewers. We strongly encourage code deposition in a community repository (e.g. GitHub). See the Nature Portfolio guidelines for submitting code & software for further information.

Data

Policy information about availability of data

All manuscripts must include a data availability statement. This statement should provide the following information, where applicable:

- Accession codes, unique identifiers, or web links for publicly available datasets
- A description of any restrictions on data availability
- For clinical datasets or third party data, please ensure that the statement adheres to our policy

Cryo-EM map of the Hcp3-Tce1 complex has been deposited at the Electron Microscopy Data Bank (EMDB) with the EMD-53274 accession code. The corresponding molecular model has been deposited at the Protein Data Bank (PDB) with accession code 9QPE.

Research involving human participants, their data, or biological material

Policy information about studies with human participants or human data. See also policy information about sex, gender (identity/presentation), and sexual orientation and race, ethnicity and racism.

Reporting on sex and gender	N/A
Reporting on race, ethnicity, or other socially relevant groupings	N/A
Population characteristics	N/A
Recruitment	N/A
Ethics oversight	N/A

Note that full information on the approval of the study protocol must also be provided in the manuscript.

Field-specific reporting

Please select the one below that is the best fit for your research. If you are not sure, read the appropriate sections before making your selection.

☒ Life sciences ☐ Behavioural & social sciences ☐ Ecological, evolutionary & environmental sciences

For a reference copy of the document with all sections, see nature.com/documents/nr-reporting-summary-flat.pdf

Life sciences study design

All studies must disclose on these points even when the disclosure is negative.

Sample size	We acquired approximately 1,039,601 particles for single particle analysis. Experiments were performed independently in triplicate (figure 1d; 2b; 4e; 5c); and duplicate (Fig 6c, e-f).
Data exclusions	Particles were excluded during 2D and 3D classification as per field conventions outlined in Extended Data Figure 2.
Replication	Statistics are calculated by splitting the dataset into two halves as per field convention of 'Gold Standard Fourier Shell Coefficient' and by calculating the map-vs-model Fourier Shell Coefficient as reported in Methods and Supplementary Figure 3.
Randomization	N/A
Blinding	N/A

Reporting for specific materials, systems and methods

We require information from authors about some types of materials, experimental systems and methods used in many studies. Here, indicate whether each material, system or method listed is relevant to your study. If you are not sure if a list item applies to your research, read the appropriate section before selecting a response.

Materials & experimental systems

n/a	Involved in the study	
☐	☒	Antibodies
☒	☐	Eukaryotic cell lines
☒	☐	Palaeontology and archaeology
☒	☐	Animals and other organisms
☒	☐	Clinical data
☒	☐	Dual use research of concern
☒	☐	Plants

Methods

n/a	Involved in the study	
☒	☐	ChIP-seq
☒	☐	Flow cytometry
☒	☐	MRI-based neuroimaging

Antibodies

Antibodies used	Rabbit anti-VSV-G (MilliporeSigma V4888, 1:5000), rabbit anti-FLAG (MilliporeSigma F7425, 1:5000), mouse anti-His (GenScript A00186-100, 1:5000), rabbit anti-Hcp3 (Genscript, 1:1000), anti-rabbit horseradish peroxidase (New England Biolabs 7074V,

	1:10000), anti-mouse horseradish peroxidase (New England Biolabs 7076S, 1:5000), mouse anti-HA (BioLegend 901503, 1:1000), mouse anti-His (Sigma Aldrich SAB2702219-100, 1:5000), anti-mouse horseradish peroxidase (Sigma Aldrich A9044, 1:80000).
Validation	Specificity of our custom Hcp3 antibody was determined by Western blotting of purified Hcp3 and comparison with a different protein purified from the same E. coli protein expression cell line. Anti-HA and Anti-His were used to confirm specificity and presence of HA-Tce1 and Hcp3-His respectively through Western Blots. This was validated through a single band in each Western Blot, corresponding to the molecular weight of each protein alongside a single band at the expected molecular weight in positive control samples and absence of signal in negative controls. Anti-Mouse was the secondary antibody used for the both Anti-HA and Anti-His Western Blots as both are Mouse monoclonal antibodies.

Plants

Seed stocks	N/A
Novel plant genotypes	N/A
Authentication	N/A
