# Supplementary figures and images for: Molecular basis of type VI secretion system effector loading
Source: Nat Microbiol. 2026 May 27;11(7):1982–94. doi: 10.1038/s41564-026-02363-x (PMC13323088; doi:10.1038/s41564-026-02363-x)

d

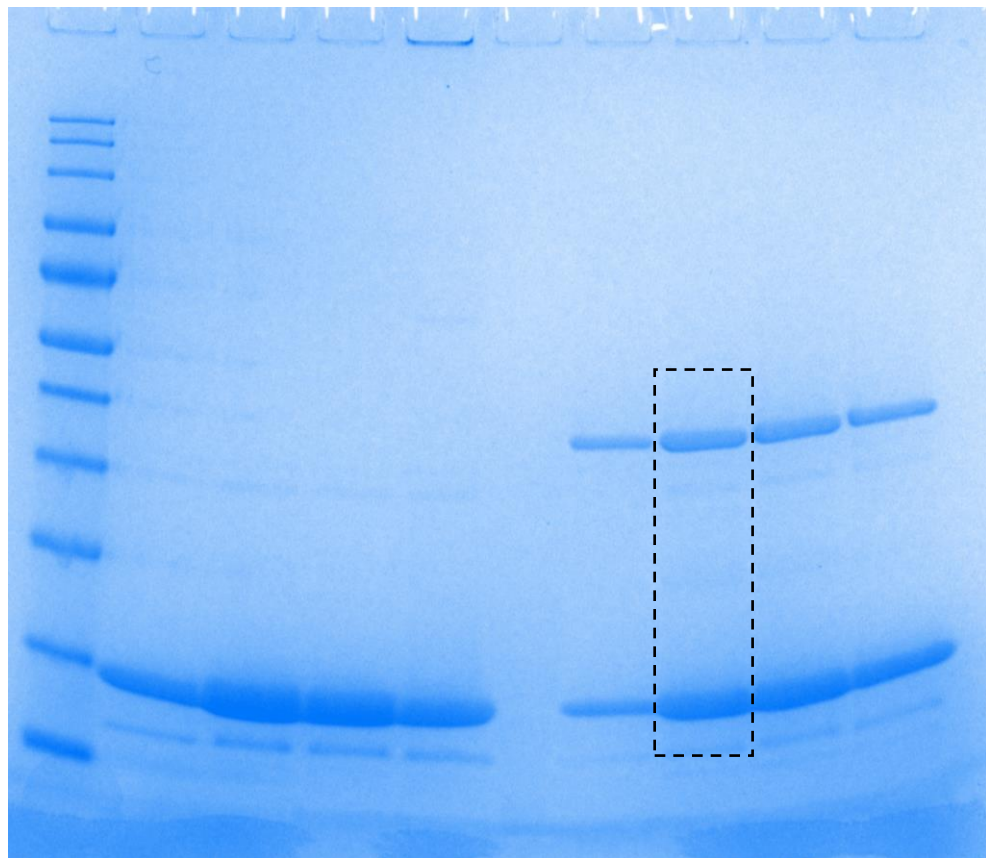

d

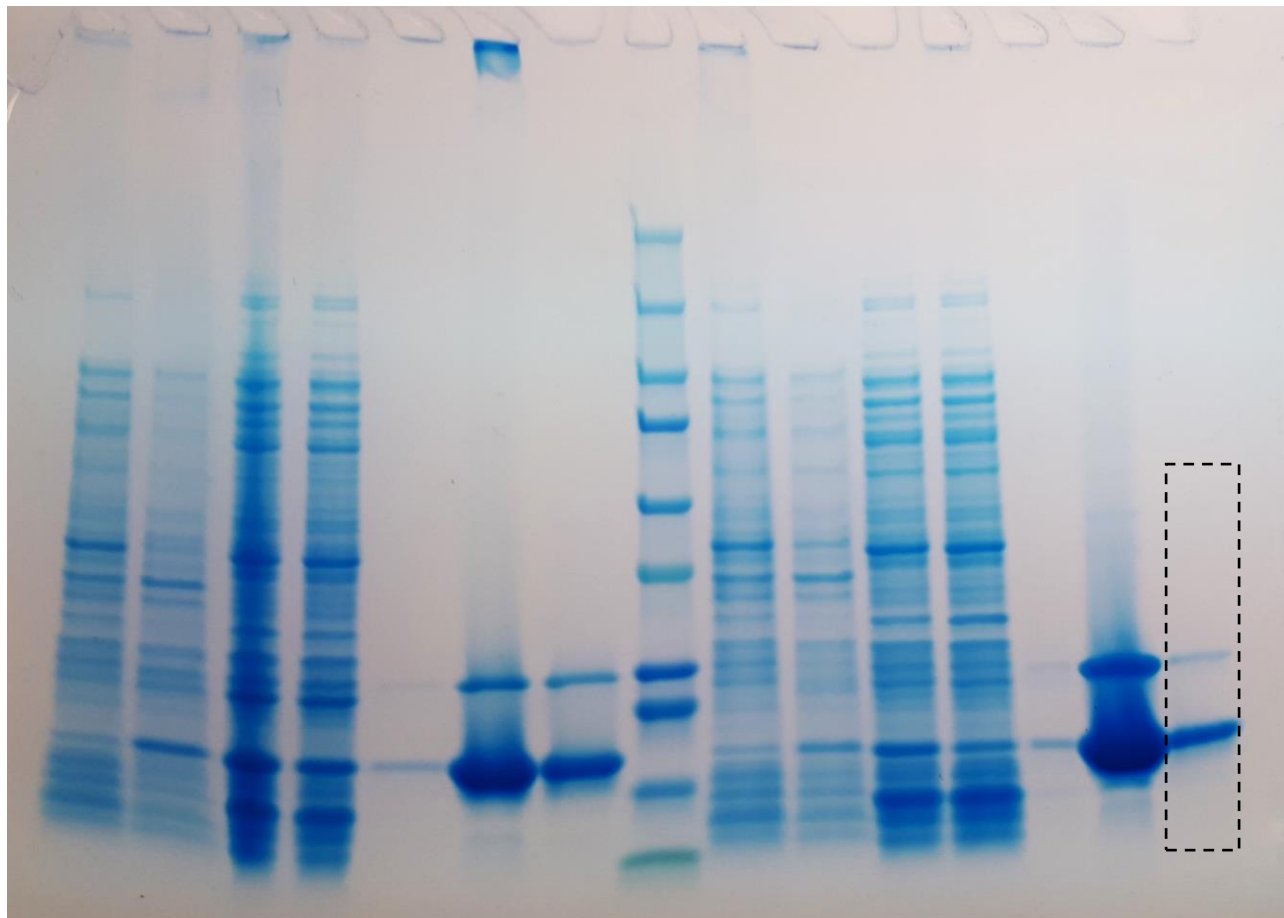

Supplement: Supplementary file 5 — Unprocessed western blots and/or gels. [file 41564_2026_2363_MOESM5_ESM.pdf]

**b**

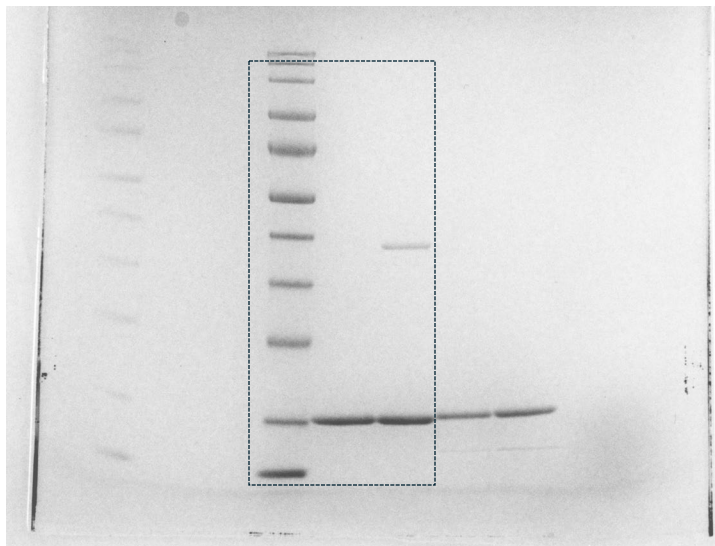

Supplement: Supplementary file 6 — Unprocessed western blots and/or gels. [file 41564_2026_2363_MOESM6_ESM.pdf]

**b**

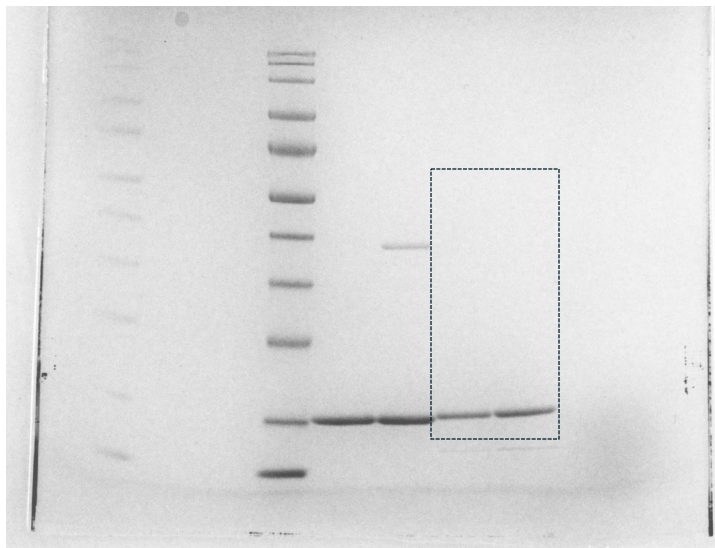

Supplement: Supplementary file 7 — Unprocessed western blots and/or gels. [file 41564_2026_2363_MOESM7_ESM.pdf]

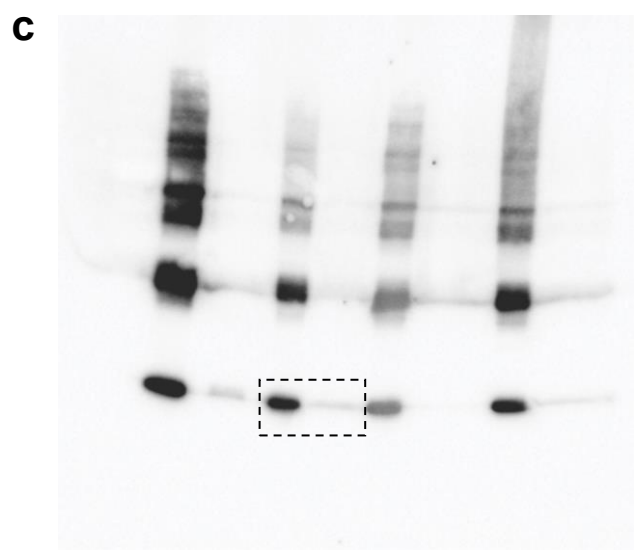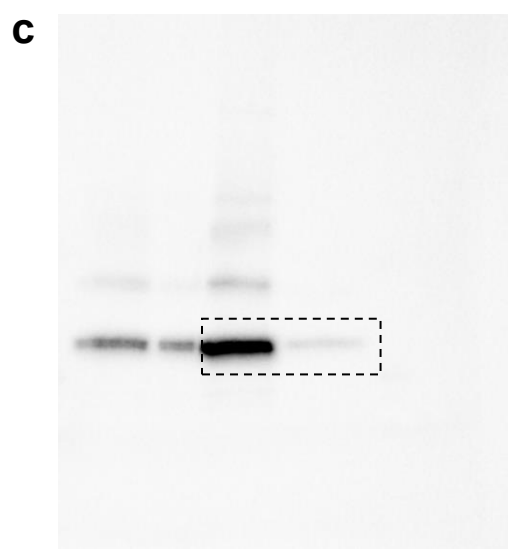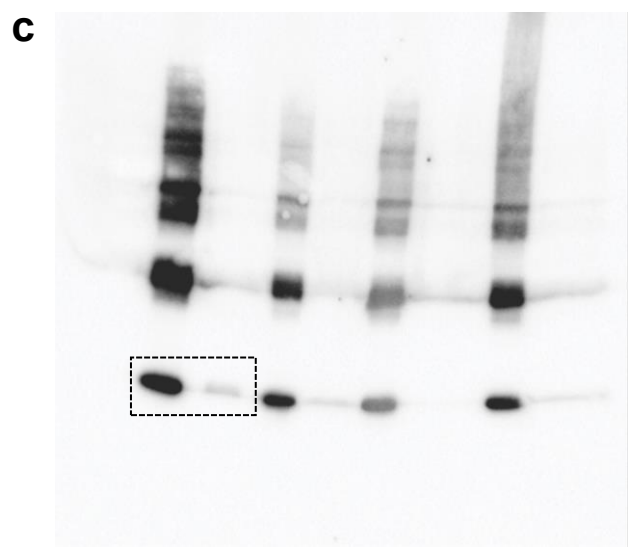

Supplement: Supplementary file 8 — Unprocessed western blots and/or gels. [file 41564_2026_2363_MOESM8_ESM.pdf]

**c**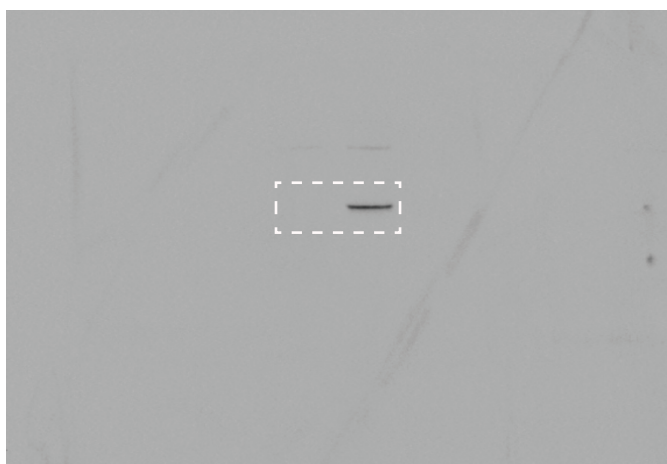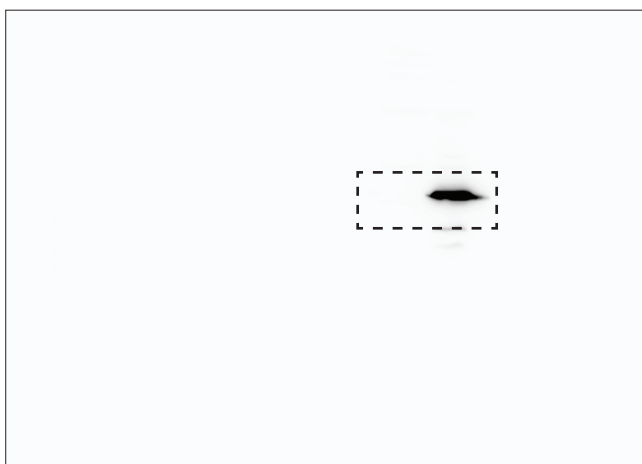**e**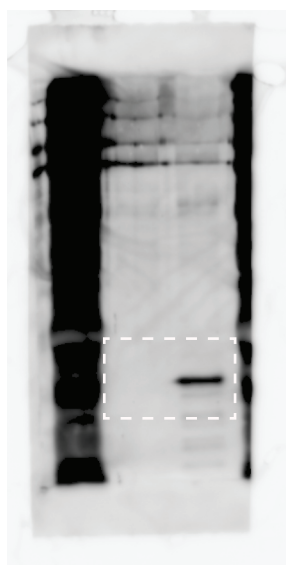**f**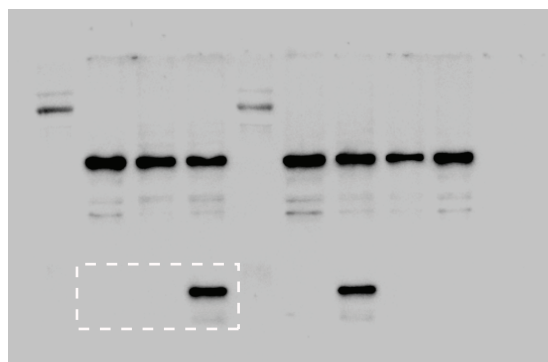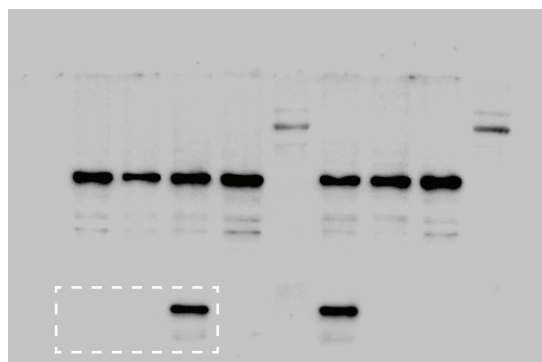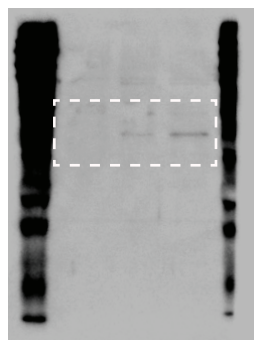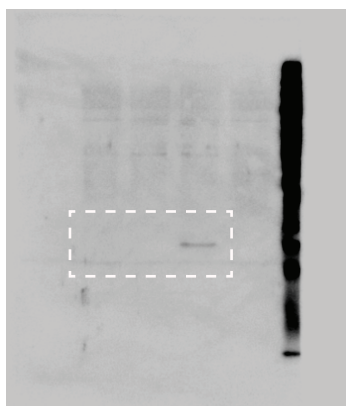

Supplement: Supplementary file 9 — Unprocessed western blots and/or gels. [file 41564_2026_2363_MOESM9_ESM.pdf]
