# Supplementary material for: Molecular basis of type VI secretion system effector loading
Source: Nat Microbiol. 2026 May 27;11(7):1982–94. doi: 10.1038/s41564-026-02363-x (PMC13323088; doi:10.1038/s41564-026-02363-x)
Supplement: Supplementary file 2 — Peer Review File [file 41564_2026_2363_MOESM2_ESM.pdf]

nature portfolio

Peer Review File

Molecular basis of type VI secretion system effector loading

Corresponding Author: Professor Alain Filloux

Version 0:

Decision Letter:

30th June 2025

Dear Alain

Thank you very much for your enquiry about submitting a manuscript to Nature Microbiology.

I've now had a chance to take a look at your summary, and think that this work, looking at how toxin cargo interactions with Hcp promote oligomerisation (and presumably inner tube formation?) sounds interesting.

Therefore, we would like to invite you to submit the full manuscript to Nature Microbiology so that we can examine the data before deciding whether to send the paper out to review. We are curious to see the structural analysis, and how additional experimental validation advances our understanding of T6SS synthesis and function over previous Hcp structural work.

If this is acceptable to you, you can submit the complete manuscript using the link below:

Link Redacted

If you have any questions, please feel free to contact me.

Yours sincerely

Version 1:

Decision Letter:

17th July 2025

Dear Alain,

I'm very sorry for the delay in getting a decision to you. Thank you for submitting the full version for your article entitled "A Novel Molecular Mechanism for Effector Protein Wrapping and Delivery by the Bacterial Type VI Secretion System". I regret to inform you that after careful discussion within the editorial team, we have decided that we will not be considering it further towards publication in Nature Microbiology.

As you may know, we decline a substantial proportion of manuscripts without sending them to referees, so that they may be sent elsewhere without delay. In such cases, even if referees were to certify the manuscript as technically correct, we consider that the work does not represent the type of advance that Nature Microbiology seeks to publish. This editorial assessment is based on considerations such as the degree of conceptual advance provided, the breadth of potential interest to researchers and timeliness.

In this case, we appreciated the careful structural and biochemical work to characterise the binding of Tce1 to Hcp3, how the effector monomer interacts with the tube protein hexamer, as well as the identification of critical residues for this interaction. We also appreciated the demonstration, through heterologous expression of Tce2 in S. cerevisiase, that Tce2 had antifungal activity. Unfortunately, after careful consideration and discussion, we felt that previous work analysing Hcp and effector interactions, and other mechanisms of effector loading onto the T6SS, meant that the overall conceptual advance presented wasn't quite at the level required for us to consider the manuscript further. Also, for the analysis of Tce2 activity - this is interesting, but unfortunately we would need a more in-depth analysis of the activity of this effector under physiologically and ecologically relevant conditions. We therefore feel that the paper would find a more suitable outlet in another journal.

Although we cannot offer to publish your manuscript, my colleagues at Communications Biology will send your manuscript out for external review. To transfer your manuscript please use our manuscript transfer portal. You will not have to re-supply manuscript metadata and files, unless you wish to make modifications. For more information, please see our manuscript transfer FAQ page.

Please be assured that this editorial decision does not represent a criticism of the quality of your work, nor are we questioning its value to others working in this area. We hope that you will rapidly receive a more favourable response elsewhere.

I am sorry that we cannot respond more positively on this occasion.

Yours sincerely

Version 2:

Reviewer comments:

Reviewer #1

(Remarks to the Author)
This is an exciting study reporting a novel mechanism by which folded proteinaceous effectors can be loaded into the tube lumen of the T6SS apparatus. The T6SS is a widespread macromolecular machine that mediates bacterial cell-cell interactions. Paracuellos & Bexter et al. show that the two proteins from the T6SS from Pseudomonas aeruginosa, Tce1 and Tce2, interact with the inner tube protein, Hcp3. The authors use Tce1 as a model and find that it is interacting with two rings of hexameric Hcp3. They also utilize cryoEM to find intermediates where the Hcp3 is assembling around a single Tce1 protein. Leading to a model wherein the Tce1/2 first interacts with a single Hcp3 protein, with others coming to surround the Tce1 and form the tube ring. They then show that while Tce1 does not have either antifungal or antibacterial activity, Tce2 was toxic to yeast when expressed from a vector. The manuscript is well written and structured. The findings represent pertinent insight into the inner tube effector loading mechanism of a T6SS, which had previously not been understood. Based on the data presented, some aspects of the model in Figure 7 remain somewhat speculative.

The authors propose an intriguing model for i) effector loading, ii) T6SS assembly, and iii) effector release after injection. The model results in an interesting discussion regarding the stoichiometries during assembly (and in the assembled state) and in particular regarding the roles of Tce1/2 as effectors, as factors in assembly and as modulators of T6SS activity. While the aspect of loading was convincingly demonstrated, the aspects of assembly and release are less well supported. In this reviewer's perspective, the disassembly of Hcp rings and release of effectors is outside the scope of the study. The aspect of the effectors' respective roles in assembly are however central to the story and could be supported by feasible in vivo approaches.

First, the authors should test individually whether Tce1 and Tce2, respectively, are secreted in a H3-T6SS-dependent manner. This has not been shown and could be done via western or mass spec.

In order to further address the roles of Tce1/2, individual and double knockout mutants could be investigated by testing H3-T6SS assembly via a GFP-sheath fusion and imaging or via the preparation of T6SS sheath or via the monitoring of secretion of Hcp in the respective mutants compared to the wildtype. It seems possible that the T6SS does not assemble in the absence of either Tce1, Tce2 or both, which would be very exciting for the discussion of the model. The fact that Tce1 did not show toxic activity may even support the hypothesis that Tce1 is in fact an assembly factor of the T6SS. It may also be insightful to generate a secretome by mass spectrometry of the wildtype and an H3-T6SS knockout mutant to see whether other cargo may be secreted besides Tce1/2. If it was only Tce1 and/or Tce2 that are secreted via the tube lumen, then it would be very straightforward to dissect their individual roles as cargo, toxin, assembly factor, activity regulator etc.

In lines 261-263 and also in their Figure 7 the authors propose a 1:1 interaction between effector and Hcp, which then nucleates assembly. Did the authors show data that supports such a 1:1 complex? The smallest complex shown by SPA cryoEM was 1:4.

In Figure 7, the authors show a disassociation of the toxin protein from the Hcp3 tube proteins, and state that it exposes the active site. However, in the assay where the Tce2 toxin is expressed in yeast, there is no condition where Tce2 and Hcp3 are expressed together, though it is shown for Tce1+Hcp3. Having a condition where Tce2+Hcp3 are expressed and testing toxicity would support the model proposed in Figure 7 where the toxin is released from the ring and exposes the active site.

Regarding the toxicity assays, the authors were unable to demonstrate a clear function or toxicity for Tce1. Additional viability assays could strengthen the study. First, including a control expressing only Hcp3 could serve as an informative baseline. Second, in vivo the T6SS is expected to deliver Hcp3 together with Tce1 and Tce2 into the target cell, a scenario that has not been tested here. An assay combining all three proteins might help elucidate the role of Tce1. It is possible that the three proteins act cooperatively, or that Tce1 contributes to regulation or enhances the toxicity of Tce2.

In Figure 5, the authors examined Tce1/Hcp3 assembly at 0.1 – 4.0 mg/mL, observing mainly non-hexameric intermediates at the lower concentrations and fully assembled hexamers at higher concentrations. The original dataset, whose 2D classifications were re-analyzed, was, however, prepared at 0.25 mg/mL – a concentration not directly tested in these experiments and only mentioned in the Methods. This makes it difficult to accurately relate the observed intermediates to the original dataset. It would also be helpful to quantify the relative abundance of these intermediates to determine whether some assembly stages are more

prevalent than others.

Could the authors comment on whether or not the residues in Hcp that are interacting with Tce2 and Tce1 are the same/conserved?

The authors should reconsider the use of very light colors, particularly in Figure 1. When printed on white paper, the label “Tce2” in Figure 1a and the AF3 models in Figure 1c are not easily visible.

Reviewer #2

(Remarks to the Author)
Paracuellos and Bexter and colleagues investigate how an effector protein is being incorporated into the inner tube of the type VI secretion system (T6SS) for delivery into other cells. Using cryo-EM, the authors present unprecedented structural details on an effector protein inside a ring of six identical Hcp proteins, which form the T6SS tube, and discover asymmetrical contact between each Hcp monomer and the effector. A combination of affinity and gel filtration chromatography reveals an interaction between an Hcp monomer and the effector prior to the formation of the hexameric ring. 2d classes suggest a step-wise process by which the hexamer of Hcp gradually forms in a concentration-dependent manner around the effector in the center of the future T6SS tube. This mechanistic work is complemented with new insight on the effector proteins themselves, which are revealed as proteases with anti-fungal activity.
In my opinion, these findings mark a major advance in our molecular understanding of protein secretion by the T6SS with broad relevance for microbe-microbe and microbe-host interactions of diverse bacterial species. Although structures of the Hcp hexamer with and without effectors have been available from early work, this study fills the gap in our mechanistic understanding of effector loading into the inner tube of the T6SS.
The experimental work, the writing, and the figures are of very high quality. I see wide implications for the fields of the T6SS, structural biology, and microbe-fungi interactions.

I list major and minor comments below that will further strengthen the manuscript in my view.

Major comments

1. Among others, the authors present two findings that I think would benefit from being linked to each other: i) the asymmetrical interaction between the Hcp monomers and the effector, ii) a stepwise process of Hcp ring formation around the effector. How does the one finding impact the other? In other words, is there a generalizable principle of the process by which the Hcp monomers form around the effector based on the interaction of the individual monomer with the effector? Unless I missed this, I did not see a link between these two observations being made, which would, in my view, provide an even more comprehensive understanding of the mechanism that the authors aim to reveal. A more detailed comment below.

L. 188f Would it be possible to determine from existing data, which Hcp monomer it is that the effector interacts with first? Or to get an estimate of the order in which the Hcp monomers are added to the effector in respect to their interaction with the effector at the interface (e.g. is Hcp3F with no interacting residues typically the last monomer added to the ring?)? An alternative approach might be to mutate the Tce1 key residues one by one and test if and how many Hcp monomers would bind to or wrap around such an effector mutant. In my opinion, this detail would enable the authors to propose a basic principle with even wider implications than the current data. Even if the data is limited in this regard and it would turn out unfeasible for technical reasons, I propose the authors to speculate on whether there is an order from the effector side by which the effector is being wrapped with Hcp monomers. Also, the implications of the authors’ data on differences in the interaction between individual Hcp monomers and the effector seems missing from the overall working model in Fig. 7.

2. The authors claim insight on a “foundational mechanism by which distinct cargos are wrapped…” (l. 39). In the presented work, it seems to me that conclusions on the mechanism are mostly based on work on Tce1 alone (and conclusions on effector function are mostly based on Tce2) and it remains unclear what exactly applies across Hcp cargo effectors. I understand that Fig. 6a tries to link the findings on Tce1 and Tce2. Given the claim on a foundational mechanism, I am wondering how much of the findings on Tce1 or which exact parts of the identified mechanism are broadly applicable. Would it be more sensible to either conclude on the mechanism of one effector here and speculate specifically on what might be broadly applicable? Or, would there be the possibility to investigate the AlphaFold model in Fig. 6a in more detail, e.g. to test if experimental work on the asymmetrical interaction of Tce1 with Hcp monomers is predicted across effectors and the interacting residues are the same for Tce1 and Tce2? (see more detailed comment below)

l. 218, “with asymmetrical contact” Despite this being a prediction, could the authors try to look in detail what the predicted interactions are of the effector to each Hcp monomer at the interfaces? Together with the data in Fig. 4b, this could help understand whether there is a generalizable principle of this asymmetrical interaction.

3. I personally struggle a bit with the phrasing of a “novel mechanism” (title, abstract, last paragraph of introduction). I clearly see novelty in the manuscript and this is not what I am questioning. In my view, the data shows unprecedented mechanistic detail while the overall existing knowledge of cargo effectors being associated with an Hcp tube for secretion by the T6SS remains untouched. I see the tendency in the text to overstate findings. For example, the abstract refers to a “conceptually novel mode of effector recognition” (l. 35f). I agree with the mechanistic detail that this study provides, I question if this phrasing captures the findings adequately because the overall idea of an effector interacting with Hcp is in my view not conceptually novel. Another

example is the claim of "two novel effector proteins… bound to Hcp3" (l. 84). I agree with the new data on these effectors being putative proteases and the data on antifungal activity of one of them. However, as accurately described by the authors in other parts of the manuscript, these putative effectors have been identified before and the phrasing that is used here might be misleading in regard to them being entirely unknown and newly identified in this study. I suggest to refine the phrasing at these and other similar instances.

Minor comments

l. 31 "loaded into ca 120 Hcp ring assemblies", I was a bit surprised by this statement. Doesn't the length of the inner tube vary quite a bit depending on along which axis the T6SSs is built (longitudinal vs vertical axis)? I could also imagine variation in the length of the inner tube across species, e.g. based on differences in cell size and shape.

Introduction: Maybe I missed this, do the authors actually write out somewhere that there are multiple studies that present protein structures of Hcp (without an effector) across organisms (e.g. ref 10 of this study, PMID: 26079269, PMID: 34906677 and others). Referring to this existing work might actually help highlighting the novelty of this study in going beyond Hcp and focusing on the interaction between Hcp and the effector.

l. 81 "equipped with 3 T6SSs" Considering work published earlier this year that is referenced at a later point in the manuscript (ref 50), isn't it now up to four T6SSs in P. aeruginosa?

l. 92 "entirely novel" L. 94 "novel mechanisms", see major comment 3.

l. 107 "multiple sequence alignment" I might have missed this, which tool was used to do the alignment? Might be good to mention this somewhere. Also on what is shown in Fig. 1a, is this part of the alignment of whole length amino acid sequences? If so, the authors might want to consider adding a supplementary figure with the entire alignment that this part is taken from.

L. 114f, "consistent with the in vivo pull-down" This refers to previous work of the authors, correct? If so, the authors might want to consider adding the respective reference here or indicate this more specifically by some other means.

l. 150ff "Because our model of Tce1 starts from Lys110" Could the authors expand on why the model of the N-terminus could not be resolved? As I understand, full-length Tce1 was used in the co-purification experiments.
Along the same lines, a comment on Fig. 3a, b. Am I correct in that this model only includes the C-terminus of Tce1? I suggest to indicate this in the figure legend and potentially in these panels of the figure already, where it currently says "Tce1". It is already indicated more specifically in panel e.

l. 162ff Just a comment on the interpretation. I agree with the described expectation that the interface of each Hcp monomer with the effector would differ. Isn't it remarkable then, that there are two residues of three Hcp monomers that are consistently involved in the interaction with very different sites of the effector? On a higher level, isn't there actually an indication for a pattern of key residues on the Hcp and effector side that seem to be consistently involved in interactions at multiple interfaces across the multi-protein complex? In my view, this would be a result that I would not have expected based on the expectation above. The impression given by "This is indeed what our structure reveals" (l. 164) did not indicate such an unexpected observation to me. I wonder if the conclusion of this section (l. 180 f "Together,…" could actually be strengthened with a more nuanced description of how the results do and do not match the expectation.

l. 195 "suggesting that ring formation is concentration dependent" Considering the experimental set-up of this being observed with purified proteins, how likely do the authors think this observation translates to what happens inside a live bacterium? For example, are the concentrations of proteins used here to observe such a concentration-dependent effect expected in a live bacterium? Could the authors maybe comment in the discussion on potential discrepancies between their experimental set up and the scenario in a live bacterium?

l. 221f, "Tce2 sits in the lower half of the Hcp3 ring, where it partially protrudes out from the underside of the ring". Wouldn't this mean that this effector is also wrapped into two Hcp rings, very much like the bigger Tce1? In the depiction in Fig. 7, it seems that there is a distinction made between an effector in one Hcp ring and an effector within two Hcp rings. Could it be a generalizable principle that Hcp-associated effectors typically require two Hcp rings? Meaning for maximal loading of an Hcp tube, it could be estimated to be maximally one effector per 12 Hcp proteins?

l. 232f "Western blot analysis revealed detectable expression of Tce2, but not Tce1". I am afraid I cannot entirely follow the authors in the text based on what I see in the figure. In Fig. 6f, isn't there a very faint band of Tse1 in the left panel that is not present in the empty vector control? I suggest to adjust the sentence above, which gave me the impression that Tce1 expression cannot be detected at all. There seems to be a bit more of Tce1 after co-expression with Hcp but just a bit more. To me, this could be described better in the text to reflect the observation more accurately.

l. 236/Fig. 6F Considering the presence of a very faint band of Tce1 full-length, it seems as if there is no band at all for Tce1CTD, potentially suggesting a role of the N-terminus in protein stability. No need to comment on this, just a thought.

L. 237 "and expressed in yeast in presence/absence of Hcp3" Might be worth checking this sentence again for the grammar.

l. 238f "Overall, we identified for the first time a T6SS dependent antifungal property that may be associated with the H3-T6SS

function through Tce2" I am a bit hesitant with this sentence. Technically speaking, I think it is correct. I am wondering if it could be misunderstood. What exactly does "for the first time" refer to here? T6SS-dependent antifungal properties in general have previously been shown. I understand that this is phrased with care because of the missing H3-T6SS activity in P. aeruginosa under laboratory conditions, it can therefore not be easily tested if the H3-T6SS of P. aeruginosa mediated antifungal activity. I would find a sentence more appropriate along the lines of: "Overall, we found antifungal properties of the Hcp3-associated effector Tce2, which is the first report of anti-fungal activity of an effector that is putatively secreted by the H3-T6SS and which suggests so far unknown anti-eukaryotic activity of the H3-T6SS."

L. 250ff "However, three specialized Hcp…" Wasn't the genomic screening that is referred to in the second half of the sentence also done by reference 42? In this case, wouldn't it be better to refer to the reference at the end of this sentence. Also, one could argue about a study from 2017 being "recent", I leave this up to the authors.

l. 256ff "Here our study reveals the detailed molecular mechanism on how cargo effectors are loaded into Hcp." Is it really sufficient data to state that the study "shows" a mechanism that applies across multiple diverse "cargo effectors"? Or does the sentence refer to "Tce1 cargo effectors"? I suggest to specify.

l. 264ff "In this process, S29, E56, and T60 serve as key hydrogen bond-forming residues, interacting with Tce1 across multiple Hcp3 protomers." To me, this came a bit out of the blue. Could this already be referred to in the corresponding results section and potentially be indicated in Fig. 4b, which residue engages in what kind of an interaction. Further, what was the rationale for listing these particular residues by name? Are these examples or is this an exhaustive list? If I understand the data correctly, isn't S31 also forming hydrogen bonds in multiple Hcps? Shouldn't this residue be listed by name here, too?
I see the value of referring to individual resides. On a higher level, the complexity could maybe be reduced. Isn't it basically about three sites of Hcp, one site around residue AA29-31, a second site around resides AA56-62, and a third site around roughly AA113-123 that matters for the interaction with Tce1 across multiple Hcp monomers?

l. 267f "compared to the previously proposed mechanism" Could the authors please provide a reference?

l. 279 "Instead, the extended Hcp may bind to 5 other 'regular' Hcps that complete the hexameric ring around one Hcp-ET." Maybe I missed it, wouldn't this sentence be an opportunity to refer to the preprint https://doi.org/10.1101/2025.01.09.632221 from earlier this year, that presents complementary data in support of this hypothesis?

l. 301 "activity" is probably not supposed to be there.

Supplementary Table 2, Does the nomenclature of Hcp3-1 to -6 here refer to Hcp3-A to -F of the main figures? Would be easier for me to have one consistent nomenclature throughout.

Fig. 7
Is it possible that this figure is not being referred to in the text?
I overall agree with a graphical depiction that summarizes the findings on the mechanism of Hcp cargo effector loading. I am struggling a bit with how this is done here. First, doesn't the data of this study apply more to the assembly scenario and less to the disassembly scenario? Isn't the disassembly scenario much more speculative and as indicated here, does this take all the forces into account that might act on the system upon delivery into the target cell when breaching membranes? My suggestion would be to either exclusively focus on the steps before firing ore indicate more clearly in figure and text whether the authors think that their data applies to both parts, pre and post firing, with equal strength. Second, to me, the depiction seems mainly based on the finding of the interaction between an Hcp protein and effector protein at a 1:1 ratio and on concentration-dependent oligomerization. Could the finding on asymmetric interactions within the tube also be included graphically and could the individual findings be linked to each other (see major comment 1)? Considering the data on Bacteroides Hcp effectors of the preprint that suggests differences between species and systems in how many Hcp proteins they might have and how this number of Hcp proteins affects effector loading, I am wondering whether it is worth to either generalize this depiction to a point that it applies to both scenarios, the one described here for the H3-T6SS of P. aeruginosa and the one for Bacteroides, or to specify more clearly that the working model presented here applies to a scenario of only one Hcp protein being associated with this system, potentially writing out H3-T6SS and P. aeruginosa in the depiction or figure legend. I would argue that such a specification does not take away novelty and impact from this work.

Reviewer #3

(Remarks to the Author)
The manuscript by Paracuellos et al., "A Novel Molecular Mechanism for Effector Protein Wrapping and Delivery by the Bacterial Type VI Secretion System", describes a mechanism for export of cargo by the HCP component of this system. Hcp3 forms hexameric rings that stack to form a tube through which some T6S effectors are transported. Building on their previous work they show that two proteins, Tce1 and Tce2, interact with Hcp3 hexamers but also Hcp3 monomers and hexamer intermediates. They solve a high quality cryoEM reconstruction of an Hcp3:Tce1 complex, revealing that Tce1 occupies the lumen of the Hcp3 ring and interacts specifically via salt bridges and hydrogen bonds. The N-terminal domain of Tce1 protrudes from the ring, suggesting it may fill the hole of the next ring in the Hcp tube. Homology modeling suggests a similar interaction with Tce2. The authors propose that Hcp3 monomers bind to the Tce cargo and oligomerize to form hexamers that assemble into the Hcp tube, and that these Hcp rings dissociate once inside the host cell, releasing the cargo. This is an elegant, novel and highly plausible mechanism. Tce1 and 2 are homologs of cysteine proteases, and Tce2 is toxic when expressed in high levels in yeast, suggesting an antifungal activity for this effector. The results are compelling and represent a significant advance in understanding T6S biology. The manuscript is well-illustrated and well-written for the most part but would benefit from some clarifications and edits. These, along with some questions, are listed below as the issues appear in the paper rather than based

on priority.

Abstract: The authors mention immune cells in the first sentence so should clarify in the introduction whether bacterial T6S systems target such eukaryotic cells. Change “ca 120 Hcp ring assemblies” to “approximately 120 Hcp ring assemblies”.

Line 67: “effectors to either one part” - change to “one component of”?

Line 87: add “structure”

Line 95-97: Sentence is awkward. Reword for clarity.

Line 100-118: Explain here and in Fig. 1 legend what the source of the proteins is (i.e. from P. aeruginosa? Recombinant protein expressed in E. coli?) and which protein is His-tagged. Same for Line 122.

Line 128: What does “following successful inspection” mean?

Line 141-143: Compare the cryoEM structure of the Hcp3 ring to the previously solved crystal structure of P. aeruginosa Hcp3 (PDB ID). Does the presence of Tce1 impact the Hcp3 ring structure? Are the Hcp3 protomers identical whether or not they contact Tce1?

Line 146-147: Delete “Our model also showed that”. Specify residue numbers for the C-terminal domain that was resolved.

Line 163-182: Consistently refer to Hcp3 subunits within the ring as protomers rather than monomers. G121 and K123 are listed as Hcp3 residues that interact with Tce1, yet these are not shown in Fig. 4. In the Discussion (Line 263-266) residues S29, E56 and T60 as listed as being key. Describe the key interactions (eg. what does G121 contribute?). For Fig. 4, protomer labels are too small and the pale text is hard to read - add an outline/shadow. Could simplify Fig. 4a by labeling the panels the same as the protomers (eg. A, B, C etc).

Line 216 (and Fig. 6 legend and elsewhere): Since proteolytic activity was not demonstrated, Tce1 and Tce2 should be called “putative” cysteine proteases. Did the authors attempt to identify Tce1/Tce2 substrates or demonstrate proteolytic activity?

Line 246: Clarify the ambiguous statement that “a cargo domain that is an extension of a T6SS component”. Why does the first paragraph of the Discussion focus on specialized effectors when Tce1 and Tce1 are cargo effector?

Line 247-249 - break into two sentences

Line 261-263: What evidence is there to support the statement that Hcp1 interaction with Tce1 nucleates hexamer formation? It also forms a hexamer in the absence of Hcp1.

Line 276: “… than initially thought” - by whom?

Line 281: “It is also striking that two effectors, Tce1 and Tce2, are packed within Hcp3 rings.” Sounds like they are present at the same time - reword. Do the authors think that the Tce1 N-terminus interaction with the second Hcp3 ring is non-specific? Could an even longer effector be accommodated?

Line 282 and elsewhere in the Discussion: The references to affinity should be qualified as affinity was not quantified.

Line 216-222: Are the Tce1 residues that interact with Hcp3 conserved in Tce2?

Line 324: “and will need” - what will need? Change to “It will be necessary to …”.

Line 339 - “sequences … were cloned” - change to “genes” or “gene fragments” were cloned

Fig. 5b: clarify that the concentrations listed are for Hcp3. What is the concentration of Tce1 in both 5a and 5b? Report in moles/molarity to show stoichiometry (or report molar ratios). Why does the hexamer peak disappear with lower Hcp3 concentrations but the monomer peak only appears at 0.1 mg/ml?

Fig. 7 is not cited in the discussion. Fig. 7 should have two paths for Hcp hexamer formation, one with and one without cargo.

Throughout the manuscript, a space is needed between numbers and units (eg. 3.4 Å, 4 ml)

Reviewer #4

(Remarks to the Author)
I enjoyed reading this manuscript and commend the authors on important, challenging work.

My review focuses on the antifungal effector aspect of the manuscript. The structural and mechanistic sections that precede this content represent the manuscript’s core strength and are supported by rigorous and well-executed experiments. The discovery that Tce2 is an antifungal effector is both interesting and novel, and the structural analysis of Tce1 loading via Hcp3 is particularly compelling. The cryo-EM reconstruction revealing a “cargo wrapping” model, wherein Hcp monomers assemble

around the effector, is a conceptually innovative contribution to the field. This mechanism is supported by complementary assays and sets the work apart from prior T6SS studies, which typically describe effectors as slotting into preformed tubes or attaching to the spike complex.
However, while the evidence supporting Tce2's antifungal activity is strong, its delivery by the H3-T6SS remains unproven in a physiological context. The study relies on heterologous expression in Saccharomyces cerevisiae to assess toxicity, which bypasses the T6SS machinery. Although the authors acknowledge this limitation and use appropriately cautious language in their conclusions, the broader claim that H3-T6SS mediates antifungal targeting is currently inferential. Demonstrating T6SS-dependent delivery in a native context such as showing that a P. aeruginosa H3-T6SS mutant fails to kill yeast or fungi that wild-type strains can, would substantively strengthen this conclusion.
Two specific suggestions to address this gap include:
1.Performing direct co-culture assays using P. aeruginosa and a eukaryotic target (e.g S. cerevisiae or Candida albicans, or Candida glabrata to keep the simplicity of a yeast experiment to test for Tce2- and H3-T6SS-dependent killing would be essential. Failure of a P. aeruginosa Tce2 or H3 T6SS mutant to outcompete fungi in co-culture would support the conclusion.
2.Investigating Tce2's mechanism of action more deeply, for instance via proteomic or genetic approaches in yeast to identify its cellular target, given its predicted cysteine protease activity would be a nice addition or future experiment.
Finally, the hypothesis that Tce1 requires auxiliary factors for activity is plausible and aligns with known dependencies of other T6SS effectors. Further work to identify potential cofactors or regulatory domains would help clarify its role. Overall, the antifungal component of the study is promising, though still preliminary in my opinion. The mechanistic work is a significant step forward, and with the additional experiment proposed above, the manuscript could establish a robust case for the inter-kingdom function of the H3-T6SS.

Minor points:
The crossbow analogy used to describe the T6SS mechanism is engaging, but the narrative oscillates between lay terminology and technical jargon. A more consistent register would improve clarity and accessibility for readers.
Protein descriptors at their first mention would improve readability and framing.

Decision Letter:

27th November 2025

*Please ensure you delete the link to your author homepage in this e-mail if you wish to forward it to your co-authors.

Dear Alain,

Thank you for your extreme patience while your manuscript "A Novel Molecular Mechanism for Effector Protein Wrapping and Delivery by the Bacterial Type VI Secretion System" was under peer-review at Nature Microbiology. I sincerely apologise for the length of time it has taken to secure these reports and get them back in from the referees, and want to reassure you that this delay in no way reflects a lack of interest in your study. The manuscript has now been seen by the referees, whose expertise and comments you will find at the end of this email. From the reports, it is clear that they also find your work of interest. However, they have raised a number of concerns that will need to be addressed before we can consider publication of the work in Nature Microbiology.

In particular, and as discussed over previous emails, several of the referees note that the aspects of the manuscript pertaining to disassembly are more speculative, and that the study should focus on the support for the intriguing assembly mechanism for which evidence is already much stronger. There are a few experiments suggested to further build this evidence, including that related to showing how assembly is disrupted in the absence of Tce1/Tce2 which should be addressed. There are also some questions relating to the broader applicability of the mechanism proposed, which referees suggest could be supported with further evidence and have also suggested approaches that could address this. Referee #1 and Referee #4 (fungi-bacteria interactions expertise) have also suggested some analyses to develop the aspects of the manuscript pertaining to the antifungal nature of the effectors. These comments are very important and should be addressed in a revised manuscript, along with the rest of the points from the referees which we believe are clear and more straightforward to address.

Should further experimental data allow you to address these criticisms, we would be happy to look at a revised manuscript.

We are committed to providing a fair and constructive peer-review process. Please do not hesitate to contact us if there are specific requests from the reviewers that you believe are technically impossible or unlikely to yield a meaningful outcome.

We strongly support public availability of data. Please place the data used in your paper into a public data repository, if one exists, or alternatively, present the data as Source Data or Supplementary Information. If data can only be shared on request, please explain why in your Data Availability Statement, and also in the correspondence with your editor. For some data types, deposition in a public repository is mandatory - more information on our data deposition policies and available repositories can be found at https://www.nature.com/nature-research/editorial-policies/reporting-standards#availability-of-data.

Please include a data availability statement as a separate section after Methods but before references, under the heading "Data Availability". This section should inform readers about the availability of the data used to support the conclusions of your study. This information includes accession codes to public repositories (data banks for protein, DNA or RNA sequences, microarray, proteomics data etc…), references to source data published alongside the paper, unique identifiers such as URLs to data repository entries, or data set DOIs, and any other statement about data availability. At a minimum, you should include the following statement: “The data that support the findings of this study are available from the corresponding author upon request”, mentioning any restrictions on availability. If DOIs are provided, we also strongly encourage including these in the Reference list (authors, title, publisher (repository name), identifier, year). For more guidance on how to write this section please see:

http://www.nature.com/authors/policies/data/data-availability-statements-data-citations.pdf

If revising your manuscript:

* Include a “Response to referees” document detailing, point-by-point, how you addressed each referee comment. If no action was taken to address a point, you must provide a compelling argument. This response will be sent back to the referees along with the revised manuscript.

* If you have not done so already we suggest that you begin to revise your manuscript so that it conforms to our Article format instructions at http://www.nature.com/nmicrobiol/info/final-submission. Refer also to any guidelines provided in this letter.

* Include a revised version of any required reporting checklist. It will be available to referees (and, potentially, statisticians) to aid in their evaluation if the manuscript goes back for peer review. A revised checklist is essential for re-review of the paper.

When submitting the revised version of your manuscript, please pay close attention to our href="https://www.nature.com/nature-portfolio/editorial-policies/image-integrity">Digital Image Integrity Guidelines. and to the following points below:

-- that unprocessed scans are clearly labelled and match the gels and western blots presented in figures.
-- that control panels for gels and western blots are appropriately described as loading on sample processing controls
-- all images in the paper are checked for duplication of panels and for splicing of gel lanes.

EXTENDED DATA FIGURES

When re-submitting your manuscript, please ensure that any supplementary figures and tables that are crucial to the manuscript’s conclusions are converted into Extended Data figures and tables to increase visibility of these data. Extended Data figures and tables are online-only (present in the online PDF and full-text HTML versions of the paper), peer-reviewed display items that provide essential background to the article but are not included in the main article due to space constraints. A maximum of ten Extended Data display items (figures and tables) is permitted.

Finally, please ensure that you retain unprocessed data and metadata files after publication, ideally archiving data in perpetuity, as these may be requested during the peer review and production process or after publication if any issues arise.

Please use the link below to submit a revised paper:

Link Redacted

Note: This url links to your confidential homepage and associated information about manuscripts you may have submitted or be reviewing for us. If you wish to forward this e-mail to co-authors, please delete this link to your homepage first.

Nature Microbiology is committed to improving transparency in authorship. As part of our efforts in this direction, we are now requesting that all authors identified as ‘corresponding author’ on published papers create and link their Open Researcher and Contributor Identifier (ORCID) with their account on the Manuscript Tracking System (MTS), prior to acceptance. This applies to primary research papers only. ORCID helps the scientific community achieve unambiguous attribution of all scholarly contributions. You can create and link your ORCID from the home page of the MTS by clicking on ‘Modify my Springer Nature account’. For more information please visit please visit www.springernature.com/orcid.

If you wish to submit a suitably revised manuscript we would hope to receive it within 6 months. If you cannot send it within this time, please let us know. We will be happy to consider your revision, even if a similar study has been accepted for publication at Nature Microbiology or published elsewhere (up to a maximum of 6 months).

In the meantime we hope that you find our referees' comments helpful.

Yours sincerely,

****
Reviewer Expertise:

Referee #1: T6SS, cryoEM
Referee #2: T6SS, Pseudomonas
Referee #3: Structural biology, secretion system/effector biology
Referee #4: fungi-bacteria interactions

Reviewer Comments:

Reviewer #1 (Remarks to the Author):

This is an exciting study reporting a novel mechanism by which folded proteinaceous effectors can be loaded into the tube lumen of the T6SS apparatus. The T6SS is a widespread macromolecular machine that mediates bacterial cell-cell interactions. Paracuellos & Bexter et al. show that the two proteins from the T6SS from Pseudomonas aeruginosa, Tce1 and Tce2, interact with the inner tube protein, Hcp3. The authors use Tce1 as a model and find that it is interacting with two rings of hexameric Hcp3. They also utilize cryoEM to find intermediates where the Hcp3 is assembling around a single Tce1 protein. Leading to a model wherein the Tce1/2 first interacts with a single Hcp3 protein, with others coming to surround the Tce1 and form the tube ring. They then show that while Tce1 does not have either antifungal or antibacterial activity, Tce2 was toxic to yeast when expressed from a vector. The manuscript is well written and structured. The findings represent pertinent insight into the inner tube effector loading mechanism of a T6SS, which had previously not been understood. Based on the data presented, some aspects of the model in Figure 7 remain somewhat speculative.

The authors propose an intriguing model for i) effector loading, ii) T6SS assembly, and iii) effector release after injection. The model results in an interesting discussion regarding the stoichiometries during assembly (and in the assembled state) and in particular regarding the roles of Tce1/2 as effectors, as factors in assembly and as modulators of T6SS activity. While the aspect of loading was convincingly demonstrated, the aspects of assembly and release are less well supported. In this reviewer's perspective, the disassembly of Hcp rings and release of effectors is outside the scope of the study. The aspect of the effectors' respective roles in assembly are however central to the story and could be supported by feasible in vivo approaches.

First, the authors should test individually whether Tce1 and Tce2, respectively, are secreted in a H3-T6SS-dependent manner. This has not been shown and could be done via western or mass spec.

In order to further address the roles of Tce1/2, individual and double knockout mutants could be investigated by testing H3-T6SS assembly via a GFP-sheath fusion and imaging or via the preparation of T6SS sheath or via the monitoring of secretion of Hcp in the respective mutants compared to the wildtype. It seems possible that the T6SS does not assemble in the absence of either Tce1, Tce2 or both, which would be very exciting for the discussion of the model. The fact that Tce1 did not show toxic activity may even support the hypothesis that Tce1 is in fact an assembly factor of the T6SS. It may also be insightful to generate a secretome by mass spectrometry of the wildtype and an H3-T6SS knockout mutant to see whether other cargo may be secreted besides Tce1/2. If it was only Tce1 and/or Tce2 that are secreted via the tube lumen, then it would be very straightforward to dissect their individual roles as cargo, toxin, assembly factor, activity regulator etc.

In lines 261-263 and also in their Figure 7 the authors propose a 1:1 interaction between effector and Hcp, which then nucleates assembly. Did the authors show data that supports such a 1:1 complex? The smallest complex shown by SPA cryoEM was 1:4.

In Figure 7, the authors show a disassociation of the toxin protein from the Hcp3 tube proteins, and state that it exposes the active site. However, in the assay where the Tce2 toxin is expressed in yeast, there is no condition where Tce2 and Hcp3 are expressed together, though it is shown for Tce1+Hcp3. Having a condition where Tce2+Hcp3 are expressed and testing toxicity would support the model proposed in Figure 7 where the toxin is released from the ring and exposes the active site.

Regarding the toxicity assays, the authors were unable to demonstrate a clear function or toxicity for Tce1. Additional viability assays could strengthen the study. First, including a control expressing only Hcp3 could serve as an informative baseline. Second, in vivo the T6SS is expected to deliver Hcp3 together with Tce1 and Tce2 into the target cell, a scenario that has not been tested here. An assay combining all three proteins might help elucidate the role of Tce1. It is possible that the three proteins act cooperatively, or that Tce1 contributes to regulation or enhances the toxicity of Tce2.

In Figure 5, the authors examined Tce1/Hcp3 assembly at 0.1 – 4.0 mg/mL, observing mainly non-hexameric intermediates at the lower concentrations and fully assembled hexamers at higher concentrations. The original dataset, whose 2D classifications were re-analyzed, was, however, prepared at 0.25 mg/mL – a concentration not directly tested in these experiments and only mentioned in the Methods. This makes it difficult to accurately relate the observed intermediates to the original dataset. It would also be helpful to quantify the relative abundance of these intermediates to determine whether some assembly stages are more prevalent than others.

Could the authors comment on whether or not the residues in Hcp that are interacting with Tce2 and Tce1 are the same/conserved?

The authors should reconsider the use of very light colors, particularly in Figure 1. When printed on white paper, the label "Tce2" in Figure 1a and the AF3 models in Figure 1c are not easily visible.

Reviewer #2 (Remarks to the Author):

Paracuellos and Bexter and colleagues investigate how an effector protein is being incorporated into the inner tube of the type VI secretion system (T6SS) for delivery into other cells. Using cryo-EM, the authors present unprecedented structural details on an effector protein inside a ring of six identical Hcp proteins, which form the T6SS tube, and discover asymmetrical contact between each Hcp monomer and the effector. A combination of affinity and gel filtration chromatography reveals an interaction between an Hcp monomer and the effector prior to the formation of the hexameric ring. 2d classes suggest a step-wise process by which the hexamer of Hcp gradually forms in a concentration-dependent manner around the effector in the center of the future T6SS tube. This mechanistic work is complemented with new insight on the effector proteins themselves, which are revealed as proteases with anti-fungal activity.
In my opinion, these findings mark a major advance in our molecular understanding of protein secretion by the T6SS with broad relevance for microbe-microbe and microbe-host interactions of diverse bacterial species. Although structures of the Hcp

hexamer with and without effectors have been available from early work, this study fills the gap in our mechanistic understanding of effector loading into the inner tube of the T6SS.
The experimental work, the writing, and the figures are of very high quality. I see wide implications for the fields of the T6SS, structural biology, and microbe-fungi interactions.

I list major and minor comments below that will further strengthen the manuscript in my view.

Major comments

1. Among others, the authors present two findings that I think would benefit from being linked to each other: i) the asymmetrical interaction between the Hcp monomers and the effector, ii) a stepwise process of Hcp ring formation around the effector. How does the one finding impact the other? In other words, is there a generalizable principle of the process by which the Hcp monomers form around the effector based on the interaction of the individual monomer with the effector? Unless I missed this, I did not see a link between these two observations being made, which would, in my view, provide an even more comprehensive understanding of the mechanism that the authors aim to reveal. A more detailed comment below.

L. 188f Would it be possible to determine from existing data, which Hcp monomer it is that the effector interacts with first? Or to get an estimate of the order in which the Hcp monomers are added to the effector in respect to their interaction with the effector at the interface (e.g. is Hcp3F with no interacting residues typically the last monomer added to the ring?)? An alternative approach might be to mutate the Tce1 key residues one by one and test if and how many Hcp monomers would bind to or wrap around such an effector mutant. In my opinion, this detail would enable the authors to propose a basic principle with even wider implications than the current data. Even if the data is limited in this regard and it would turn out unfeasible for technical reasons, I propose the authors to speculate on whether there is an order from the effector side by which the effector is being wrapped with Hcp monomers. Also, the implications of the authors' data on differences in the interaction between individual Hcp monomers and the effector seems missing from the overall working model in Fig. 7.

2. The authors claim insight on a "foundational mechanism by which distinct cargos are wrapped…" (l. 39). In the presented work, it seems to me that conclusions on the mechanism are mostly based on work on Tce1 alone (and conclusions on effector function are mostly based on Tce2) and it remains unclear what exactly applies across Hcp cargo effectors. I understand that Fig. 6a tries to link the findings on Tce1 and Tce2. Given the claim on a foundational mechanism, I am wondering how much of the findings on Tce1 or which exact parts of the identified mechanism are broadly applicable. Would it be more sensible to either conclude on the mechanism of one effector here and speculate specifically on what might be broadly applicable? Or, would there be the possibility to investigate the AlphaFold model in Fig. 6a in more detail, e.g. to test if experimental work on the asymmetrical interaction of Tce1 with Hcp monomers is predicted across effectors and the interacting residues are the same for Tce1 and Tce2? (see more detailed comment below)

l. 218, "with asymmetrical contact" Despite this being a prediction, could the authors try to look in detail what the predicted interactions are of the effector to each Hcp monomer at the interfaces? Together with the data in Fig. 4b, this could help understand whether there is a generalizable principle of this asymmetrical interaction.

3. I personally struggle a bit with the phrasing of a "novel mechanism" (title, abstract, last paragraph of introduction). I clearly see novelty in the manuscript and this is not what I am questioning. In my view, the data shows unprecedented mechanistic detail while the overall existing knowledge of cargo effectors being associated with an Hcp tube for secretion by the T6SS remains untouched. I see the tendency in the text to overstate findings. For example, the abstract refers to a "conceptually novel mode of effector recognition" (l. 35f). I agree with the mechanistic detail that this study provides, I question if this phrasing captures the findings adequately because the overall idea of an effector interacting with Hcp is in my view not conceptually novel. Another example is the claim of "two novel effector proteins… bound to Hcp3" (l. 84). I agree with the new data on these effectors being putative proteases and the data on antifungal activity of one of them. However, as accurately described by the authors in other parts of the manuscript, these putative effectors have been identified before and the phrasing that is used here might be misleading in regard to them being entirely unknown and newly identified in this study. I suggest to refine the phrasing at these and other similar instances.

Minor comments

l. 31 "loaded into ca 120 Hcp ring assemblies", I was a bit surprised by this statement. Doesn't the length of the inner tube vary quite a bit depending on along which axis the T6SSs is built (longitudinal vs vertical axis)? I could also imagine variation in the length of the inner tube across species, e.g. based on differences in cell size and shape.

Introduction: Maybe I missed this, do the authors actually write out somewhere that there are multiple studies that present protein structures of Hcp (without an effector) across organisms (e.g. ref 10 of this study, PMID: 26079269, PMID: 34906677 and others). Referring to this existing work might actually help highlighting the novelty of this study in going beyond Hcp and focusing on the interaction between Hcp and the effector.

l. 81 "equipped with 3 T6SSs" Considering work published earlier this year that is referenced at a later point in the manuscript (ref 50), isn't it now up to four T6SSs in P. aeruginosa?

l. 92 “entirely novel” L. 94 “novel mechanisms”, see major comment 3.

l. 107 “multiple sequence alignment” I might have missed this, which tool was used to do the alignment? Might be good to mention this somewhere. Also on what is shown in Fig. 1a, is this part of the alignment of whole length amino acid sequences? If so, the authors might want to consider adding a supplementary figure with the entire alignment that this part is taken from.

L. 114f, “consistent with the in vivo pull-down” This refers to previous work of the authors, correct? If so, the authors might want to consider adding the respective reference here or indicate this more specifically by some other means.

l. 150ff “Because our model of Tce1 starts from Lys110” Could the authors expand on why the model of the N-terminus could not be resolved? As I understand, full-length Tce1 was used in the co-purification experiments.
Along the same lines, a comment on Fig. 3a, b. Am I correct in that this model only includes the C-terminus of Tce1? I suggest to indicate this in the figure legend and potentially in these panels of the figure already, where it currently says “Tce1”. It is already indicated more specifically in panel e.

l. 162ff Just a comment on the interpretation. I agree with the described expectation that the interface of each Hcp monomer with the effector would differ. Isn’t it remarkable then, that there are two residues of three Hcp monomers that are consistently involved in the interaction with very different sites of the effector? On a higher level, isn’t there actually an indication for a pattern of key residues on the Hcp and effector side that seem to be consistently involved in interactions at multiple interfaces across the multi-protein complex? In my view, this would be a result that I would not have expected based on the expectation above. The impression given by “This is indeed what our structure reveals” (l. 164) did not indicate such an unexpected observation to me. I wonder if the conclusion of this section (l. 180 f “Together,…” could actually be strengthened with a more nuanced description of how the results do and do not match the expectation.

l. 195 “suggesting that ring formation is concentration dependent” Considering the experimental set-up of this being observed with purified proteins, how likely do the authors think this observation translates to what happens inside a live bacterium? For example, are the concentrations of proteins used here to observe such a concentration-dependent effect expected in a live bacterium? Could the authors maybe comment in the discussion on potential discrepancies between their experimental set up and the scenario in a live bacterium?

l. 221f, “Tce2 sits in the lower half of the Hcp3 ring, where it partially protrudes out from the underside of the ring”. Wouldn’t this mean that this effector is also wrapped into two Hcp rings, very much like the bigger Tce1? In the depiction in Fig. 7, it seems that there is a distinction made between an effector in one Hcp ring and an effector within two Hcp rings. Could it be a generalizable principle that Hcp-associated effectors typically require two Hcp rings? Meaning for maximal loading of an Hcp tube, it could be estimated to be maximally one effector per 12 Hcp proteins?

l. 232f “Western blot analysis revealed detectable expression of Tce2, but not Tce1”. I am afraid I cannot entirely follow the authors in the text based on what I see in the figure. In Fig. 6f, isn’t there a very faint band of Tse1 in the left panel that is not present in the empty vector control? I suggest to adjust the sentence above, which gave me the impression that Tce1 expression cannot be detected at all. There seems to be a bit more of Tce1 after co-expression with Hcp but just a bit more. To me, this could be described better in the text to reflect the observation more accurately.

l. 236/Fig. 6F Considering the presence of a very faint band of Tce1 full-length, it seems as if there is no band at all for Tce1CTD, potentially suggesting a role of the N-terminus in protein stability. No need to comment on this, just a thought.

L. 237 “and expressed in yeast in presence/absence of Hcp3” Might be worth checking this sentence again for the grammar.

l. 238f “Overall, we identified for the first time a T6SS dependent antifungal property that may be associated with the H3-T6SS function through Tce2” I am a bit hesitant with this sentence. Technically speaking, I think it is correct. I am wondering if it could be misunderstood. What exactly does “for the first time” refer to here? T6SS-dependent antifungal properties in general have previously been shown. I understand that this is phrased with care because of the missing H3-T6SS activity in P. aeruginosa under laboratory conditions, it can therefore not be easily tested if the H3-T6SS of P. aeruginosa mediated antifungal activity. I would find a sentence more appropriate along the lines of: “Overall, we found antifungal properties of the Hcp3-associated effector Tce2, which is the first report of anti-fungal activity of an effector that is putatively secreted by the H3-T6SS and which suggests so far unknown anti-eukaryotic activity of the H3-T6SS.”

L. 250ff “However, three specialized Hcp…” Wasn’t the genomic screening that is referred to in the second half of the sentence also done by reference 42? In this case, wouldn’t it be better to refer to the reference at the end of this sentence. Also, one could argue about a study from 2017 being “recent”, I leave this up to the authors.

l. 256ff “Here our study reveals the detailed molecular mechanism on how cargo effectors are loaded into Hcp.” Is it really sufficient data to state that the study “shows” a mechanism that applies across multiple diverse “cargo effectors”? Or does the sentence refer to “Tce1 cargo effectors”? I suggest to specify.

l. 264ff “In this process, S29, E56, and T60 serve as key hydrogen bond-forming residues, interacting with Tce1 across multiple Hcp3 protomers.” To me, this came a bit out of the blue. Could this already be referred to in the corresponding results section and potentially be indicated in Fig. 4b, which residue engages in what kind of an interaction. Further, what was the rationale for listing these particular residues by name? Are these examples or is this an exhaustive list? If I understand the data correctly, isn’t S31 also forming hydrogen bonds in multiple Hcps? Shouldn’t this residue be listed by name here, too?
I see the value of referring to individual resides. On a higher level, the complexity could maybe be reduced. Isn’t it basically

about three sites of Hcp, one site around residue AA29-31, a second site around resides AA56-62, and a third site around roughly AA113-123 that matters for the interaction with Tce1 across multiple Hcp monomers?

l. 267f “compared to the previously proposed mechanism” Could the authors please provide a reference?

l. 279 “Instead, the extended Hcp may bind to 5 other ‘regular’ Hcps that complete the hexameric ring around one Hcp-ET.” Maybe I missed it, wouldn’t this sentence be an opportunity to refer to the preprint https://doi.org/10.1101/2025.01.09.632221 from earlier this year, that presents complementary data in support of this hypothesis?

l. 301 “activity” is probably not supposed to be there.

Supplementary Table 2, Does the nomenclature of Hcp3-1 to -6 here refer to Hcp3-A to -F of the main figures? Would be easier for me to have one consistent nomenclature throughout.

Fig. 7
Is it possible that this figure is not being referred to in the text?
I overall agree with a graphical depiction that summarizes the findings on the mechanism of Hcp cargo effector loading. I am struggling a bit with how this is done here. First, doesn’t the data of this study apply more to the assembly scenario and less to the disassembly scenario? Isn’t the disassembly scenario much more speculative and as indicated here, does this take all the forces into account that might act on the system upon delivery into the target cell when breaching membranes? My suggestion would be to either exclusively focus on the steps before firing ore indicate more clearly in figure and text whether the authors think that their data applies to both parts, pre and post firing, with equal strength. Second, to me, the depiction seems mainly based on the finding of the interaction between an Hcp protein and effector protein at a 1:1 ratio and on concentration-dependent oligomerization. Could the finding on asymmetric interactions within the tube also be included graphically and could the individual findings be linked to each other (see major comment 1)? Considering the data on Bacteroides Hcp effectors of the preprint that suggests differences between species and systems in how many Hcp proteins they might have and how this number of Hcp proteins affects effector loading, I am wondering whether it is worth to either generalize this depiction to a point that it applies to both scenarios, the one described here for the H3-T6SS of P. aeruginosa and the one for Bacteroides, or to specify more clearly that the working model presented here applies to a scenario of only one Hcp protein being associated with this system, potentially writing out H3-T6SS and P. aeruginosa in the depiction or figure legend. I would argue that such a specification does not take away novelty and impact from this work.

Reviewer #3 (Remarks to the Author):

The manuscript by Paracuellos et al., “A Novel Molecular Mechanism for Effector Protein Wrapping and Delivery by the Bacterial Type VI Secretion System”, describes a mechanism for export of cargo by the HCP component of this system. Hcp3 forms hexameric rings that stack to form a tube through which some T6S effectors are transported. Building on their previous work they show that two proteins, Tce1 and Tce2, interact with Hcp3 hexamers but also Hcp3 monomers and hexamer intermediates. They solve a high quality cryoEM reconstruction of an Hcp3:Tce1 complex, revealing that Tce1 occupies the lumen of the Hcp3 ring and interacts specifically via salt bridges and hydrogen bonds. The N-terminal domain of Tce1 protrudes from the ring, suggesting it may fill the hole of the next ring in the Hcp tube. Homology modeling suggests a similar interaction with Tce2. The authors propose that Hcp3 monomers bind to the Tce cargo and oligomerize to form hexamers that assemble into the Hcp tube, and that these Hcp rings dissociate once inside the host cell, releasing the cargo. This is an elegant, novel and highly plausible mechanism. Tce1 and 2 are homologs of cysteine proteases, and Tce2 is toxic when expressed in high levels in yeast, suggesting an antifungal activity for this effector. The results are compelling and represent a significant advance in understanding T6S biology. The manuscript is well-illustrated and well-written for the most part but would benefit from some clarifications and edits. These, along with some questions, are listed below as the issues appear in the paper rather than based on priority.

Abstract: The authors mention immune cells in the first sentence so should clarify in the introduction whether bacterial T6S systems target such eukaryotic cells. Change “ca 120 Hcp ring assemblies” to “approximately 120 Hcp ring assemblies”.

Line 67: “effectors to either one part” - change to “one component of”?

Line 87: add “structure”

Line 95-97: Sentence is awkward. Reword for clarity.

Line 100-118: Explain here and in Fig. 1 legend what the source of the proteins is (i.e. from P. aeruginosa? Recombinant protein expressed in E. coli?) and which protein is His-tagged. Same for Line 122.

Line 128: What does “following successful inspection” mean?

Line 141-143: Compare the cryoEM structure of the Hcp3 ring to the previously solved crystal structure of P. aeruginosa Hcp3 (PDB ID). Does the presence of Tce1 impact the Hcp3 ring structure? Are the Hcp3 protomers identical whether or not they contact Tce1?

Line 146-147: Delete “Our model also showed that”. Specify residue numbers for the C-terminal domain that was resolved.

Line 163-182: Consistently refer to Hcp3 subunits within the ring as protomers rather than monomers. G121 and K123 are listed as Hcp3 residues that interact with Tce1, yet these are not shown in Fig. 4. In the Discussion (Line 263-266) residues S29, E56 and T60 as listed as being key. Describe the key interactions (eg. what does G121 contribute?). For Fig. 4, protomer labels are too small and the pale text is hard to read - add an outline/shadow. Could simplify Fig. 4a by labeling the panels the same as the protomers (eg. A, B, C etc).

Line 216 (and Fig. 6 legend and elsewhere): Since proteolytic activity was not demonstrated, Tce1 and Tce2 should be called "putative" cysteine proteases. Did the authors attempt to identify Tce1/Tce2 substrates or demonstrate proteolytic activity?

Line 246: Clarify the ambiguous statement that "a cargo domain that is an extension of a T6SS component". Why does the first paragraph of the Discussion focus on specialized effectors when Tce1 and Tce1 are cargo effector?

Line 247-249 - break into two sentences

Line 261-263: What evidence is there to support the statement that Hcp1 interaction with Tce1 nucleates hexamer formation? It also forms a hexamer in the absence of Hcp1.

Line 276: "… than initially thought" - by whom?

Line 281: "It is also striking that two effectors, Tce1 and Tce2, are packed within Hcp3 rings." Sounds like they are present at the same time - reword. Do the authors think that the Tce1 N-terminus interaction with the second Hcp3 ring is non-specific? Could an even longer effector be accommodated?

Line 282 and elsewhere in the Discussion: The references to affinity should be qualified as affinity was not quantified.

Line 216-222: Are the Tce1 residues that interact with Hcp3 conserved in Tce2?

Line 324: "and will need" - what will need? Change to "It will be necessary to …".

Line 339 - "sequences … were cloned" - change to "genes" or "gene fragments" were cloned

Fig. 5b: clarify that the concentrations listed are for Hcp3. What is the concentration of Tce1 in both 5a and 5b? Report in moles/molarity to show stoichiometry (or report molar ratios). Why does the hexamer peak disappear with lower Hcp3 concentrations but the monomer peak only appears at 0.1 mg/ml?

Fig. 7 is not cited in the discussion. Fig. 7 should have two paths for Hcp hexamer formation, one with and one without cargo.

Throughout the manuscript, a space is needed between numbers and units (eg. 3.4 Å, 4 ml)

Reviewer #4 (Remarks to the Author):

I enjoyed reading this manuscript and commend the authors on important, challenging work.

My review focuses on the antifungal effector aspect of the manuscript. The structural and mechanistic sections that precede this content represent the manuscript's core strength and are supported by rigorous and well-executed experiments. The discovery that Tce2 is an antifungal effector is both interesting and novel, and the structural analysis of Tce1 loading via Hcp3 is particularly compelling. The cryo-EM reconstruction revealing a "cargo wrapping" model, wherein Hcp monomers assemble around the effector, is a conceptually innovative contribution to the field. This mechanism is supported by complementary assays and sets the work apart from prior T6SS studies, which typically describe effectors as slotting into preformed tubes or attaching to the spike complex.
However, while the evidence supporting Tce2's antifungal activity is strong, its delivery by the H3-T6SS remains unproven in a physiological context. The study relies on heterologous expression in Saccharomyces cerevisiae to assess toxicity, which bypasses the T6SS machinery. Although the authors acknowledge this limitation and use appropriately cautious language in their conclusions, the broader claim that H3-T6SS mediates antifungal targeting is currently inferential. Demonstrating T6SS-dependent delivery in a native context such as showing that a P. aeruginosa H3-T6SS mutant fails to kill yeast or fungi that wild-type strains can, would substantively strengthen this conclusion.
Two specific suggestions to address this gap include:
1.Performing direct co-culture assays using P. aeruginosa and a eukaryotic target (e.g S. cerevisiae or Candida albicans, or Candida glabrata to keep the simplicity of a yeast experiment to test for Tce2- and H3-T6SS-dependent killing would be essential. Failure of a P. aeruginosa Tce2 or H3 T6SS mutant to outcompete fungi in co-culture would support the conclusion.
2.Investigating Tce2's mechanism of action more deeply, for instance via proteomic or genetic approaches in yeast to identify its cellular target, given its predicted cysteine protease activity would be a nice addition or future experiment.
Finally, the hypothesis that Tce1 requires auxiliary factors for activity is plausible and aligns with known dependencies of other T6SS effectors. Further work to identify potential cofactors or regulatory domains would help clarify its role. Overall, the antifungal component of the study is promising, though still preliminary in my opinion. The mechanistic work is a significant step forward, and with the additional experiment proposed above, the manuscript could establish a robust case for the inter-kingdom function of the H3-T6SS.

Minor points:

The crossbow analogy used to describe the T6SS mechanism is engaging, but the narrative oscillates between lay terminology and technical jargon. A more consistent register would improve clarity and accessibility for readers.
Protein descriptors at their first mention would improve readability and framing.

Version 3:

Reviewer comments:

Reviewer #1

(Remarks to the Author)
The authors have adequately addressed our previous comments.
Congratulations on an exciting story.
Charles Ericson/David Schaumann/Martin Pilhofer

Reviewer #2

(Remarks to the Author)
I thank the authors for all of their additional work, my comments have been addressed to my satisfaction.
When making the final edits, I encourage the authors to include a reference to new work (PMID: 41723119) that was published in the meantime and does not overlap with the work presented here but is of relevance to the topic in the big picture.
I came across two more typos, l. 152 repeat of “in”; l. 400 “Hcp’s” is thought to be plural here, isn’t it? How about a phrasing as “Hcp proteins”?

Reviewer #3

(Remarks to the Author)
The authors have addressed my comments and appear to have addressed those of the other reviewers. I heartily support publication of this manuscript in Nature Microbiology.

Reviewer #4

(Remarks to the Author)
I appreciate that the authors have carefully considered my previous comments and have provided thoughtful and reasonable responses. I agree that the additional experiments suggested to directly address antifungal activity and molecular targets would constitute a substantial body of work potentially beyond the scope of the current study. The authors’ position is scientifically defensible, and in my view the manuscript is suitable for publication in its current form.

Decision Letter:

18th March 2026

*Please ensure you delete the link to your author homepage in this e-mail if you wish to forward it to your co-authors.

Dear Professor Filloux,

Thank you for your patience while your manuscript "Effector-guided nucleation of Hcp3 ring assembly reveals principles of cargo loading into the type VI secretion system" was under peer-review at Nature Microbiology. It has now been seen by the original 4 referees, whose expertise and comments you will find at the end of this email. You will see from their comments below that they find that the revised manuscript addresses all their previous concerns and that they support publication of the work in Nature Microbiology. As a result, we are very happy to say that we will be accepting-in-principle this manuscript, pending any formatting changes to comply with journal policies, and to address the minor point raised by Referee #2.

As part of the accept-in -principle process, we run all manuscripts through a series of checks. In order for us to move forwards with this step, please could we ask you to do a quick revision, in which you resupply the supplementary figures as Extended Data figures. If you can rename them, and reupload them in this way, we can then push forwards with the checks.

Thanks a lot for your help with this, and many thanks for submitting your work to the journal.

When revising your paper:

* resubmit electronically if possible using the link below to access your home page:

Link Redacted

*This url links to your confidential homepage and associated information about manuscripts you may have submitted or be reviewing for us. If you wish to forward this e-mail to co-authors, please delete this link to your homepage first.

Please ensure that all correspondence is marked with your Nature Microbiology reference number in the subject line.

Nature Microbiology is committed to improving transparency in authorship. As part of our efforts in this direction, we are now requesting that all authors identified as ‘corresponding author’ on published papers create and link their Open Researcher and Contributor Identifier (ORCID) with their account on the Manuscript Tracking System (MTS), prior to acceptance. This applies to primary research papers only. ORCID helps the scientific community achieve unambiguous attribution of all scholarly contributions. You can create and link your ORCID from the home page of the MTS by clicking on ‘Modify my Springer Nature account’. For more information please visit please visit www.springernature.com/orcid.

We hope to receive your revised paper within a week. If you cannot send it within this time, please let us know.

We look forward to hearing from you soon.

Yours sincerely,

****

Reviewer Expertise:

Referee #1: structural biology, secretion systems
Referee #2: T6SS
Referee #3: structural biology, secretion systems/effectors
Referee #4: bacteria-fungi interactions

Reviewers Comments:

Reviewer #1 (Remarks to the Author):

The authors have adequately addressed our previous comments.
Congratulations on an exciting story.
Charles Ericson/David Schaumann/Martin Pilhofer

Reviewer #2 (Remarks to the Author):

I thank the authors for all of their additional work, my comments have been addressed to my satisfaction.
When making the final edits, I encourage the authors to include a reference to new work (PMID: 41723119) that was published in the meantime and does not overlap with the work presented here but is of relevance to the topic in the big picture.
I came across two more typos, l. 152 repeat of “in”; l. 400 “Hcp’s” is thought to be plural here, isn’t it? How about a phrasing as “Hcp proteins”?

Reviewer #3 (Remarks to the Author):

The authors have addressed my comments and appear to have addressed those of the other reviewers. I heartily support publication of this manuscript in Nature Microbiology.

Reviewer #4 (Remarks to the Author):

I appreciate that the authors have carefully considered my previous comments and have provided thoughtful and reasonable responses. I agree that the additional experiments suggested to directly address antifungal activity and molecular targets would constitute a substantial body of work potentially beyond the scope of the current study. The authors’ position is scientifically defensible, and in my view the manuscript is suitable for publication in its current form.

****

Version 4:

Decision Letter:

Our ref: NMICROBIOL-25062311D

23rd March 2026

Dear Dr. Filloux,

Thank you for submitting your revised manuscript "Effector-guided nucleation of Hcp3 ring assembly reveals principles of cargo loading into the type VI secretion system" (NMICROBIOL-25062311D). We'll be happy in principle to publish it in Nature Microbiology, pending minor revisions to comply with our editorial and formatting guidelines.

We are now performing detailed checks on your paper and will send you a checklist detailing our editorial and formatting requirements in about a week. Please do not upload the final materials and make any revisions until you receive this additional information from us.

Thank you again for your interest in Nature Microbiology Please do not hesitate to contact me if you have any questions.

Sincerely,

Version 5:

Decision Letter:

17th April 2026

Dear Professor Filloux,

I am pleased to accept your Article "Molecular basis of type VI secretion system effector loading" for publication in Nature Microbiology. Thank you for having chosen to submit your work to us and many congratulations.

Over the next few weeks, your paper will be copyedited to ensure that it conforms to Nature Microbiology style.

Once your paper is typeset, you will receive an email with a link to choose the appropriate publishing options for your paper and our Author Services team will be in touch regarding any additional information that may be required. Once your paper has been scheduled for online publication, the Nature press office will be in touch to confirm the details.

You may wish to make your media relations office aware of your accepted publication, in case they consider it appropriate to organize some internal or external publicity. Once your paper has been scheduled you will receive an email confirming the publication details. This is normally 3-4 working days in advance of publication. If you need additional notice of the date and time of publication, please let the production team know when you receive the proof of your article to ensure there is sufficient time to coordinate. Further information on our embargo policies can be found here:
https://www.nature.com/authors/policies/embargo.html

After the grant of rights is completed, you will receive a link to your electronic proof via email with a request to make any corrections within 48 hours. If, when you receive your proof, you cannot meet this deadline, please inform us at rjsproduction@springernature.com immediately. You will not receive your proofs until the publishing agreement has been received through our system

Due to the importance of these deadlines, we ask you please us know now whether you will be difficult to contact over the next month. If this is the case, we ask you provide us with the contact information (email, phone and fax) of someone who will be able to check the proofs on your behalf, and who will be available to address any last-minute problems.

Acceptance of your manuscript is conditional on all authors' agreement with our publication policies (see https://www.nature.com/nmicrobiol/editorial-policies). In particular your manuscript must not be published elsewhere.

Authors may need to take specific actions to achieve compliance with funder and institutional open access mandates. If your research is supported by a funder that requires immediate open access (e.g. according to Plan S principles or the NIH public access policy) then you should select the gold OA route, and we will direct you to the compliant route where possible. Because authors warrant under our subscription licensing terms that they haven’t committed to licensing any version of their article under a licence inconsistent with the terms of our agreement – including the applicable embargo period – publication under the subscription model isn’t suitable for authors whose funders require no embargo.

If you have any questions about our publishing options, costs, Open Access requirements, or our legal forms, please contact ASJournals@springernature.com

An online order form for reprints of your paper is available at https://www.nature.com/reprints/author-reprints.html. All co-authors, authors' institutions and authors' funding

agencies can order reprints using the form appropriate to their geographical region.

We welcome the submission of potential cover material (including a short caption of around 40 words) related to your manuscript; suggestions should be sent to Nature Microbiology as electronic files (the image should be 300 dpi at 210 x 297 mm in either TIFF or JPEG format). Please note that such pictures should be selected more for their aesthetic appeal than for their scientific content, and that colour images work better than black and white or grayscale images. Please do not try to design a cover with the Nature Microbiology logo etc., and please do not submit composites of images related to your work. I am sure you will understand that we cannot make any promise as to whether any of your suggestions might be selected for the cover of the journal.

You can now use a single sign-on for all your accounts, view the status of all your manuscript submissions and reviews, access usage statistics for your published articles and download a record of your refereeing activity for the Nature journals.

To assist our authors in disseminating their research to the broader community, our SharedIt initiative provides you with a unique shareable link that will allow anyone (with or without a subscription) to read the published article. Recipients of the link with a subscription will also be able to download and print the PDF.

As soon as your article is published, you will receive an automated email with your shareable link.

With kind regards,

P.S. Click on the following link if you would like to recommend Nature Microbiology to your librarian http://www.nature.com/subscriptions/recommend.html#forms

** Visit the Springer Nature Editorial and Publishing website at www.springernature.com/editorial-and-publishing-jobs for more information about our career opportunities. If you have any questions please click here.**

Open Access This Peer Review File is licensed under a Creative Commons Attribution 4.0 International License, which permits use, sharing, adaptation, distribution and reproduction in any medium or format, as long as you give appropriate credit to the original author(s) and the source, provide a link to the Creative Commons license, and indicate if changes were made.
In cases where reviewers are anonymous, credit should be given to 'Anonymous Referee' and the source.
The images or other third party material in this Peer Review File are included in the article's Creative Commons license, unless indicated otherwise in a credit line to the material. If material is not included in the article's Creative Commons license and your intended use is not permitted by statutory regulation or exceeds the permitted use, you will need to obtain permission directly from the copyright holder.
To view a copy of this license, visit https://creativecommons.org/licenses/by/4.0/

Manuscript NMICROBIOL-25062311B-Z by Paracuellos *et al.*,

ANSWERS TO REVIEWERS

We thank all four reviewers and the editor for the careful review of our manuscript and for providing insightful comments that we have now addressed and included in a revised version of the paper. Here below we provide a point-by-point answer, shown in blue, to all reviewer's comments. You will noticed that we have added on author in the list, Sujatha, Subramoni, since she performed additional experiments included in our manuscript and notably the microscopy to monitor T6SS sheath assemblies. We believe that with this we will have addressed satisfactorily all issues raised and we hope that our work will now be deemed suitable for publication in *Nature Microbiology*.

Key experiments we have now included in response to the reviewers' comments are, i) confirmed that H3-T6SS sheath assembly is independent of the presence of Tce1 or Tce2 (Supp Fig. 7), ii) confirmed the existence of a 1:1 Hcp3–Tce1 complex (new Fig. 5b); iii) confirmed Tce2 toxicity and absence of Tce1 toxicity in various context where one or more of Tce1, Tce2 or Hcp3 are (co-) expressed in yeast (Supp Fig. 6); iv) confirmed residues from Tce1 and Tce2 that are conserved to interact with Hcp3; v) highlighted the asymmetric Hcp3–Tce1 interface by presenting a model coloured by B-factor. In this representation, the B-factor reflects the local flexibility or mobility of each region in the structure (Supp Fig. 4); vi) modified the figure 7 for the model to reflect accurately on our new findings.

Reviewer #1 (Remarks to the Author):

This is an exciting study reporting a novel mechanism by which folded proteinaceous effectors can be loaded into the tube lumen of the T6SS apparatus. The T6SS is a widespread macromolecular machine that mediates bacterial cell-cell interactions. Paracuellos & Bexter et al. show that the two proteins from the T6SS from Pseudomonas aeruginosa, Tce1 and Tce2, interact with the inner tube protein, Hcp3. The authors use Tce1 as a model and find that it is interacting with two rings of hexameric Hcp3. They also utilize cryoEM to find intermediates where the Hcp3 is assembling around a single Tce1 protein. Leading to a model wherein the Tce1/2 first interacts with a single Hcp3 protein, with others coming to surround the Tce1 and form the tube ring. They then show that while Tce1 does not have either antifungal or antibacterial activity, Tce2 was toxic to yeast when expressed from a vector. The manuscript is well written and structured. The findings represent pertinent insight into the inner tube effector loading mechanism of a T6SS, which had previously not been understood. Based on the data presented, some aspects of the model in Figure 7 remain somewhat speculative.

We thank the reviewer for providing a compelling and comprehensive description of our work, and for identifying the key novel and original concepts which are presented and which are moving forward T6SS research.

The authors propose an intriguing model for i) effector loading, ii) T6SS assembly, and iii) effector release after injection. The model results in an interesting discussion regarding the stoichiometries during assembly (and in the assembled state) and in particular regarding the roles of Tce1/2 as effectors, as factors in assembly and as modulators of T6SS activity. While the aspect of loading was convincingly demonstrated, the aspects of assembly and release are less well supported. In this reviewer's perspective, the disassembly of Hcp rings and release of effectors is outside the scope of the study. The aspect of the effectors' respective roles in assembly are however central to the story and could be supported by feasible in vivo approaches.

We agree with the reviewer that elaborating on the mechanism of release of the effector into the target cell is beyond the scope of the present study and we will respond further below to the suggestions proposed for further validating the effector's roles.

First, the authors should test individually whether Tce1 and Tce2, respectively, are secreted in a H3-T6SS-dependent manner. This has not been shown and could be done via western or mass spec.

This is a very valid point which unfortunately cannot be addressed experimentally. We had already responded to a similar comment when we published our work in *mBio* (Howard et al., 2021) in which we used a Hcp3 FLAG-tagged and pull down PA0256 and PA2372, renamed in the present study Tce1 and Tce2. The main reason for not being able to monitor secretion is that although the H3-T6SS is highly expressed, we have not found conditions in which it actively secretes in our *in vitro* condition. As such no Hcp3 could be seen in the supernatant and therefore no Tce1 or Tce2 either. One possible reason could be that the H3-T6SS assembly starts form the pole (Allsopp et al., PNAS, 2017) and therefore the extension/contraction needed for the firing event must go all the way longitudinally and might not be very effective, at least in the conditions we have worked so far. We are still actively searching H3-T6SS effective secretion conditions but have not yet succeeded (e.g. various media, various temperature, contact with eukaryotic cells etc…).

We have included a sentence in the results section to flag the lack of secretion effectiveness of the H3-T6SS (lines 271-273).

In order to further address the roles of Tce1/2, individual and double knockout mutants could be investigated by testing H3-T6SS assembly via a GFP-sheath fusion and imaging or via the preparation of T6SS sheath or via the monitoring of secretion of Hcp in the respective mutants compared to the wildtype. It seems possible that the T6SS does not assemble in the absence of either Tce1, Tce2 or both, which would be very exciting for the discussion of the model. The fact that Tce1 did not show toxic activity may even support the hypothesis that Tce1 is in fact an assembly factor of the T6SS. It may also be insightful to generate a secretome by mass spectrometry of the wildtype and an H3-T6SS knockout mutant to see whether other cargo may be secreted besides Tce1/2. If it was only Tce1 and/or Tce2 that are secreted via the tube lumen, then it would be very straightforward to dissect their individual roles as cargo, toxin, assembly factor, activity regulator etc.

This is a very interesting point, and it also refers to previous papers suggesting that the T6SS firing in some bacterial species has an onboarding checking mechanism so that the system does not fire if not loaded (Liang et al., PNAS, 2019). We have thus generated mutants in *tce1*, *tce2*, double *tce1/tce2*, *hcp3* and *vgrG3* in a *P. aeruginosa rsmA* strain expressing TssB3-sfGFP from the native *tssB3* locus (as described in Bernal et al. PNAS, 2021). Fluorescence microscopy imaging of H3-T6SS sheaths was subsequently performed using these strains. What we observed is that, as expected, if Hcp3 or VgrG3 are missing no T6SS sheath assembly could be seen. However, for the *tce1*, *tce2* and even the *tce1/tce2* double mutants, sheath assembly is very similar to what is observed in the parental strain. Here again it is seen that the sheath preferentially assembled at the pole.

These data have now been included in the supplementary figure 7 and described in the main text (lines 273-279) and the materials and methods (lines 541-555).

The fact that the sheath assembled in a *tce1/tce2* double mutant suggests that additional effectors for the H3-T6SS are still loaded. The suggestion by the reviewers to comprehensively identified those effectors using a comparative mass spec analysis of the supernatant of a H3-

T6SS mutant vs wild-type cannot be conducted for the reason described above based on the lack of secretion activity of this system.

In lines 261-263 and also in their Figure 7 the authors propose a 1:1 interaction between effector and Hcp, which then nucleates assembly. Did the authors show data that supports such a 1:1 complex? The smallest complex shown by SPA cryoEM was 1:4.

We thank the reviewer for highlighting this point. We have now included a chromatogram of molecular-weight standards demonstrating that the high-elution-volume peak (15.4 ml) corresponds to a species consistent with a 1:1 Hcp3–Tce1 complex molecular-weight (revised Fig. 5b).

In Figure 7, the authors show a disassociation of the toxin protein from the Hcp3 tube proteins, and state that it exposes the active site. However, in the assay where the Tce2 toxin is expressed in yeast, there is no condition where Tce2 and Hcp3 are expressed together, though it is shown for Tce1+Hcp3. Having a condition where Tce2+Hcp3 are expressed and testing toxicity would support the model proposed in Figure 7 where the toxin is released from the ring and exposes the active site.

Regarding the toxicity assays, the authors were unable to demonstrate a clear function or toxicity for Tce1. Additional viability assays could strengthen the study. First, including a control expressing only Hcp3 could serve as an informative baseline. Second, in vivo the T6SS is expected to deliver Hcp3 together with Tce1 and Tce2 into the target cell, a scenario that has not been tested here. An assay combining all three proteins might help elucidate the role of Tce1. It is possible that the three proteins act cooperatively, or that Tce1 contributes to regulation or enhances the toxicity of Tce2.

We thank the reviewer for identifying this gap and to address this we shuffled around some vectors to allow for compatibility and simultaneous or alternate expression of Tce1, Hcp3 and Tce2. As described before only Tce2 expression induces significant toxicity. Furthermore in a background where all three proteins, Hcp3, Tce1 and Tce2 are expressed, there is no change to toxicity when Tce2 is expressed in the presence of Tce1 and Hcp - therefore no enhanced toxicity can be detected when all three are present.

These data have now been included in supplementary figure 6 and described in the main text (lines 265-268) as well as in the methods.

In Figure 5, the authors examined Tce1/Hcp3 assembly at 0.1 – 4.0 mg/mL, observing mainly non-hexameric intermediates at the lower concentrations and fully assembled hexamers at higher concentrations. The original dataset, whose 2D classifications were re-analysed, was, however, prepared at 0.25 mg/mL – a concentration not directly tested in these experiments and only mentioned in the Methods. This makes it difficult to accurately relate the observed intermediates to the original dataset. It would also be helpful to quantify the relative abundance of these intermediates to determine whether some assembly stages are more prevalent than others.

We thank the reviewer for this thoughtful comment. The concentrations used in the biochemical co-purification of Tce1 with Hcp3 cannot be directly compared with those used for the cryo-EM dataset because the experimental setups differed in an important aspect. For cryo-EM, we could not employ higher concentrations of the Hcp3-Tce1 mixture (>0.25 mg/mL), as this would have resulted in particle densities too high for reliable imaging and data processing. Therefore, to ensure that the majority of Hcp3 rings were occupied by Tce1 at the 0.25 mg/mL concentration used for cryo-EM, the sample was treated with 0.1%

glutaraldehyde, which stabilized the pre-assembled complexes and prevented their disassembly during grid preparation.

We have included a brief comment on this in the methods section (lines 435-439).

Could the authors comment on whether or not the residues in Hcp that are interacting with Tce2 and Tce1 are the same/conserved?

We thank the reviewer for this helpful observation. Yes, several Hcp3 residues involved in effector binding are conserved between the Tce1 and Tce2 interactions. Using PDBePISA analysis of the AlphaFold3 Hcp3-Tce2 model (Supplementary Fig. 5), we identified S29, S31, E56, T60, N113, and S115 of Hcp3 as conserved interacting residues shared with the experimentally determined Hcp3-Tce1 complex. In addition to this common core interface, Hcp3 forms further hydrogen bonds or salt bridges with each effector through distinct residues, consistent with a model combining conserved and effector-specific interactions.

We have included a brief comment on this in the main text (lines 242-247).

The authors should reconsider the use of very light colors, particularly in Figure 1. When printed on white paper, the label "Tce2" in Figure 1a and the AF3 models in Figure 1c are not easily visible.

We thank the reviewer for this helpful observation. We have adjusted the colour scheme in Figure 1 to improve contrast and ensure that all labels and structural models, including "Tce2" in Fig. 1a and the AF3 models in Fig. 1c, are clearly visible in print.

Reviewer #2 (Remarks to the Author):

Paracuellos and Bexter and colleagues investigate how an effector protein is being incorporated into the inner tube of the type VI secretion system (T6SS) for delivery into other cells. Using cryo-EM, the authors present unprecedented structural details on an effector protein inside a ring of six identical Hcp proteins, which form the T6SS tube, and discover asymmetrical contact between each Hcp monomer and the effector. A combination of affinity and gel filtration chromatography reveals an interaction between an Hcp monomer and the effector prior to the formation of the hexameric ring. 2d classes suggest a step-wise process by which the hexamer of Hcp gradually forms in a concentration-dependent manner around the effector in the center of the future T6SS tube. This mechanistic work is complemented with new insight on the effector proteins themselves, which are revealed as proteases with anti-fungal activity.

In my opinion, these findings mark a major advance in our molecular understanding of protein secretion by the T6SS with broad relevance for microbe-microbe and microbe-host interactions of diverse bacterial species. Although structures of the Hcp hexamer with and without effectors have been available from early work, this study fills the gap in our mechanistic understanding of effector loading into the inner tube of the T6SS.

The experimental work, the writing, and the figures are of very high quality. I see wide implications for the fields of the T6SS, structural biology, and microbe-fungi interactions.

We are grateful to the reviewers for highlighting the quality and relevance of our work in the T6SS research field.

I list major and minor comments below that will further strengthen the manuscript in my view.

Major comments

1. Among others, the authors present two findings that I think would benefit from being linked to each other: i) the asymmetrical interaction between the Hcp monomers and the effector, ii) a stepwise process of Hcp ring formation around the effector. How does the one finding impact the other? In other words, is there a generalizable principle of the process by which the Hcp monomers form around the effector based on the interaction of the individual monomer with the effector? Unless I missed this, I did not see a link between these two observations being made, which would, in my view, provide an even more comprehensive understanding of the mechanism that the authors aim to reveal. A more detailed comment below.

We thank the reviewer for this insightful comment. An asymmetrical interaction between Hcp monomers and an effector is expected because the effector itself lacks six-fold symmetry. As a result, each Hcp protomer in the ring necessarily engages the effector to a different extent and through distinct residues.

The relationship between points (i) and (ii) emerges from our finding that Hcp ring formation proceeds through a stepwise, concentration-dependent assembly mechanism, both in the presence and absence of an effector. This was our first key observation. Building on it, we asked whether (a) Hcp hexamers assemble first and subsequently recruit the effector, or (b) the effector initially engages one (or a few) Hcp protomers, after which the concentration-driven assembly process completes the ring around the bound effector.

Our data support the latter model. We observe an initial 1:1 interaction between a single Hcp protomer and Tce1. In vivo, particularly in the cytoplasm during co-expression, the interface with the strongest set of interacting residues is the one most likely to nucleate binding. After this initial capture, increasing Hcp concentration favours the recruitment of additional protomers. These incoming Hcps interact both with neighbouring Hcps and with the effector, with the degree and nature of effector contact determined by which region of Tce1 each protomer faces.

Thus, the asymmetrical binding interface is not only expected - it plays a mechanistic role in guiding the stepwise assembly of the Hcp ring around the effector. This integration of the two observations provides a more comprehensive view of how effector loading occurs during Hcp ring formation.

We have made a clearer statement in the discussion to describe the above. (lines 298-302, 312-321).

L. 188f Would it be possible to determine from existing data, which Hcp monomer it is that the effector interacts with first? Or to get an estimate of the order in which the Hcp monomers are added to the effector in respect to their interaction with the effector at the interface (e.g. is Hcp3F with no interacting residues typically the last monomer added to the ring?)? An alternative approach might be to mutate the Tce1 key residues one by one and test if and how many Hcp monomers would bind to or wrap around such an effector mutant. In my opinion, this detail would enable the authors to propose a basic principle with even wider implications than the current data. Even if the data is limited in this regard and it would turn out unfeasible for technical reasons, I propose the authors to speculate on whether there is an order from the effector side by which the effector is being wrapped with Hcp monomers. Also, the implications of the authors' data on differences in the interaction between individual Hcp monomers and the effector seems missing from the overall working model in Fig. 7.

We appreciate the reviewer's thoughtful suggestion. Determining the precise order in which individual Hcp protomers bind to the effector would indeed be an interesting line of

investigation, however several factors limit the feasibility and interpretability of such an analysis. In the Hcp3-Tce1 structure, five of the six Hcp protomers make hydrogen-bond interactions with Tce1. Therefore, mutating individual Tce1 residues would not prevent interaction with the remaining protomers. Even if the strongest-contact residue were disrupted, multiple alternative interfaces would still support initial binding to at least one Hcp monomer. Once nucleated, ring formation would proceed via the same concentration-dependent mechanism, ultimately leading to encapsulation of the effector. For these reasons, mutational analysis of individual Tce1 contact residues would be unlikely to reveal a definitive or generalizable order of Hcp recruitment.

Nonetheless, we agree that the asymmetry in Hcp–effector interactions have conceptual implications for how the ring may assemble around the effector. To illustrate this point, we have now included a Hcp3–Tce1 model coloured by B-factor as Supplementary Figure 4. In this representation, the B-factor (or atomic displacement parameter) reflects the local flexibility or mobility of each region in the structure - higher values indicate greater dynamic movement or reduced structural order. Visualizing the complex in this way highlights variation in rigidity among the Hcp protomers and their interfaces with Tce1, further emphasizing the asymmetry that may influence the sequence of protomer engagement during ring assembly.

We have made a clearer statement in the discussion to describe the above. (line 304-309).

2. The authors claim insight on a "foundational mechanism by which distinct cargos are wrapped…" (l. 39). In the presented work, it seems to me that conclusions on the mechanism are mostly based on work on Tce1 alone (and conclusions on effector function are mostly based on Tce2) and it remains unclear what exactly applies across Hcp cargo effectors. I understand that Fig. 6a tries to link the findings on Tce1 and Tce2. Given the claim on a foundational mechanism, I am wondering how much of the findings on Tce1 or which exact parts of the identified mechanism are broadly applicable. Would it be more sensible to either conclude on the mechanism of one effector here and speculate specifically on what might be broadly applicable? Or, would there be the possibility to investigate the AlphaFold model in Fig. 6a in more detail, e.g. to test if experimental work on the asymmetrical interaction of Tce1 with Hcp monomers is predicted across effectors and the interacting residues are the same for Tce1 and Tce2? (see more detailed comment below).

We thank the reviewer's comment and the opportunity to clarify the generality of the mechanism we propose. A central principle that emerges from our work is that T6SS cargo effectors engage their cognate Hcp rings asymmetrically. This follows inherently from the fact that effectors do not possess six-fold symmetry; consequently, each Hcp protomer encounters a different structural surface of the effector and therefore forms a distinct set of interactions. As a result, some protomers inevitably contribute more extensively to effector binding than others. This asymmetry is thus a conserved and unavoidable feature of all Hcp-effector pairs.

This principle is supported by previous structural studies of *P. aeruginosa* Hcp1 bound to multiple effectors (Tse1-4), where the interacting Hcp1 residues include both a shared core set and residues uniquely used by each effector (https://doi.org/10.1016/j.molcel.2013.07.025, https://doi.org/10.1128/mbio.00262-21**)**. Our findings extend this concept: in both the experimentally determined Hcp3-Tce1 structure and the AlphaFold3-predicted Hcp3-Tce2 complex, we observe the same pattern - a subset of Hcp3 residues is consistently used for effector engagement, while each effector also relies on a unique set of contacts (Supplementary Figure 5).

Thus, although our mechanistic conclusions are experimentally derived from Tce1, the underlying principles - namely asymmetric engagement and the combination of conserved and

effector-specific interfaces may be broadly applicable across T6SS cargo effectors. We have clarified this point in the revised manuscript and now more explicitly distinguish between the elements of the mechanism that are generalizable and those that are specific to Tce1 (lines 236-248).

l. 218, "with asymmetrical contact" Despite this being a prediction, could the authors try to look in detail what the predicted interactions are of the effector to each Hcp monomer at the interfaces? Together with the data in Fig. 4b, this could help understand whether there is a generalizable principle of this asymmetrical interaction.

We thank the reviewer for this helpful suggestion. Although the Hcp3-Tce2 complex is based on an AlphaFold prediction, we agree that a detailed comparison of the predicted interaction interfaces can provide additional insight into whether the asymmetrical binding pattern is conserved. We have therefore added a table summarizing the predicted Tce2–Hcp3 contacts and compared their sequence and structural locations with those observed experimentally for Tce1 (Supplementary figure 5). This analysis highlights both a shared set of Hcp3 residues used across the two effectors and effector-specific contact sites, reinforcing the general principle that asymmetric engagement arises from a combination of conserved and unique interfaces.

3. I personally struggle a bit with the phrasing of a "novel mechanism" (title, abstract, last paragraph of introduction). I clearly see novelty in the manuscript and this is not what I am questioning. In my view, the data shows unprecedented mechanistic detail while the overall existing knowledge of cargo effectors being associated with an Hcp tube for secretion by the T6SS remains untouched. I see the tendency in the text to overstate findings. For example, the abstract refers to a "conceptually novel mode of effector recognition" (l. 35f). I agree with the mechanistic detail that this study provides, I question if this phrasing captures the findings adequately because the overall idea of an effector interacting with Hcp is in my view not conceptually novel. Another example is the claim of "two novel effector proteins… bound to Hcp3" (l. 84). I agree with the new data on these effectors being putative proteases and the data on antifungal activity of one of them. However, as accurately described by the authors in other parts of the manuscript, these putative effectors have been identified before and the phrasing that is used here might be misleading in regard to them being entirely unknown and newly identified in this study. I suggest to refine the phrasing at these and other similar instances.

We thank the reviewer for this valuable feedback. We have revised the abstract, and main text to avoid overstating novelty and to ensure that our phrasing accurately reflects the scope of our findings. The title now reads "Effector-guided nucleation of Hcp3 ring assembly reveals principles of cargo loading in the T6SS".

Minor comments

l. 31 "loaded into ca 120 Hcp ring assemblies", I was a bit surprised by this statement. Doesn't the length of the inner tube vary quite a bit depending on along which axis the T6SSs is built (longitudinal vs vertical axis)? I could also imagine variation in the length of the inner tube across species, e.g. based on differences in cell size and shape.

We thank the reviewer for this helpful observation. The reviewer is correct that the number of Hcp rings may vary substantially, as T6SSs can assemble either along the cell body or at polar sites, and this variation also occurs across species with different cell sizes and shapes. Yet, it is important to suggest the concept that the inner tube is likely made of over 100 Hcp rings as we have now indicated in our revised manuscript.

Introduction: Maybe I missed this, do the authors actually write out somewhere that there are multiple studies that present protein structures of Hcp (without an effector) across organisms (e.g. ref 10 of this study, PMID: 26079269, PMID: 34906677 and others). Referring to this existing work might actually help highlighting the novelty of this study in going beyond Hcp and focusing on the interaction between Hcp and the effector.

We have now indicated in the main text that, despite many Hcp structures in different bacterial species having been reported, and we have inserted all corresponding references, no studies ever reported the structure of an Hcp-Effector complex (lines 291-292).

l. 81 "equipped with 3 T6SSs" Considering work published earlier this year that is referenced at a later point in the manuscript (ref 50), isn't it now up to four T6SSs in P. aeruginosa?

There is indeed a fourth systems that has been described in *P. aeruginosa* (Habich et al., Nat Commun, 2025) so we have changed the text accordingly (lines 82-83).

l. 92 "entirely novel" L. 94 "novel mechanisms", see major comment 3.

We have modified these statements accordingly and the sentence now reads: "Overall, our work defines a distinct effector loading mechanism and establishes the molecular principles governing how T6SS cargo complexes are recruited by and loaded into Hcp" (lines 95-97).

l. 107 "multiple sequence alignment" I might have missed this, which tool was used to do the alignment? Might be good to mention this somewhere. Also on what is shown in Fig. 1a, is this part of the alignment of whole length amino acid sequences? If so, the authors might want to consider adding a supplementary figure with the entire alignment that this part is taken from.

We thank the reviewer for this helpful suggestion. The multiple sequence alignment was performed using Clustal Omega, and we now specify this in the Methods (lines 486-489). Figure 1a shows the relevant region extracted from the full-length sequence alignment. We have now added the complete alignment as a Supplementary Figure 5 for clarity.

L. 114f, "consistent with the in vivo pull-down" This refers to previous work of the authors, correct? If so, the authors might want to consider adding the respective reference here or indicate this more specifically by some other means.

We thank the reviewer for this comment. This statement does indeed refer to our previously published in vivo pull-down data, and we have now added the appropriate reference (Howard *et al.*, *mBio*, 2021) to the revised manuscript (line 119).

l. 150ff "Because our model of Tce1 starts from Lys110" Could the authors expand on why the model of the N-terminus could not be resolved? As I understand, full-length Tce1 was used in the co-purification experiments.

We thank the reviewer for this comment. Although full-length Tce1 was used for co-purification, only the portion that is accommodated within the Hcp3 hexamer is resolved in the cryo-EM reconstruction. Because full-length Tce1 cannot be entirely loaded into a single Hcp ring, its N-terminal domain is expected to protrude outside the hexamer. This exposed region is therefore highly flexible, resulting in low signal-to-noise during image averaging and preventing reliable density reconstruction for this domain. We have added a sentence in the manuscript to explain this (lines 154-157).

Along the same lines, a comment on Fig. 3a, b. Am I correct in that this model only includes the C-terminus of Tce1? I suggest to indicate this in the figure legend and potentially in these panels of the figure already, where it currently says "Tce1". It is already indicated more specifically in panel e.

We thank the reviewer for this helpful suggestion. The reviewer is correct that the model shown in Fig. 3a,b includes only the C-terminal portion of Tce1. We have now clarified this by labelling it as “Tce1$^{C\text{-terminus}}$“ in the figure panels and updating the figure legend accordingly.

l. 162ff Just a comment on the interpretation. I agree with the described expectation that the interface of each Hcp monomer with the effector would differ. Isn’t it remarkable then, that there are two residues of three Hcp monomers that are consistently involved in the interaction with very different sites of the effector? On a higher level, isn’t there actually an indication for a pattern of key residues on the Hcp and effector side that seem to be consistently involved in interactions at multiple interfaces across the multi-protein complex? In my view, this would be a result that I would not have expected based on the expectation above. The impression given by “This is indeed what our structure reveals” (l. 164) did not indicate such an unexpected observation to me. I wonder if the conclusion of this section (l. 180 f “Together,…” could actually be strengthened with a more nuanced description of how the results do and do not match the expectation.

We thank the reviewer for this insightful interpretative comment. We agree that, while asymmetry in Hcp–effector interfaces is expected, it is indeed striking that a small set of Hcp residues is repeatedly reused by multiple protomers to engage distinct regions of the effector. This observation suggests that, beyond global asymmetry, there exists a conserved pattern of “hotspot” residues on the Hcp side that can participate in multiple, topologically distinct interfaces. We have revised the corresponding section of the Results (including lines 172–185) to better reflect this nuance. The text now explicitly distinguishes between our initial expectation of broadly variable interfaces and the unexpected finding that certain Hcp residues are consistently repurposed across different protomer–effector contacts. We also strengthened the concluding statement of this section to emphasize that Hcp–effector recognition reflects a combination of conserved interaction hotspots and effector-specific contacts, rather than purely stochastic asymmetry (lines 193-198).

l. 195 “suggesting that ring formation is concentration dependent” Considering the experimental set-up of this being observed with purified proteins, how likely do the authors think this observation translates to what happens inside a live bacterium? For example, are the concentrations of proteins used here to observe such a concentration-dependent effect expected in a live bacterium? Could the authors maybe comment in the discussion on potential discrepancies between their experimental set up and the scenario in a live bacterium?

We thank the reviewer for this important point. The absolute intracellular concentrations of Hcp and its cognate effectors are currently not well defined and are likely to be influenced by multiple additional factors in the cytoplasmic environment, including local protein crowding, spatial organization, and T6SS assembly dynamics. Our *in vitro* experiments were therefore designed to capture the intrinsic biophysical propensity of Hcp to undergo concentration-dependent assembly. While the precise concentrations used in vitro may not directly mirror those in vivo, the observed behaviour reveals a fundamental property of the system that is likely modulated in the cell by regulatory and spatial constraints. We now comment on these potential differences between the *in vitro* and *in vivo* scenarios in the Discussion (lines 359-364).

l. 221f, “Tce2 sits in the lower half of the Hcp3 ring, where it partially protrudes out from the underside of the ring”. Wouldn’t this mean that this effector is also wrapped into two Hcp rings, very much like the bigger Tce1? In the depiction in Fig. 7, it seems that there is a distinction made between an effector in one Hcp ring and an effector within two Hcp rings. Could it be a generalizable principle that Hcp-associated effectors typically require two Hcp rings? Meaning

for maximal loading of an Hcp tube, it could be estimated to be maximally one effector per 12 Hcp proteins?

We thank the reviewer for this thoughtful interpretation. Based on the AlphaFold prediction, Tce2 indeed occupies the lower half of the Hcp3 ring and likely protrudes toward the lumen of the adjacent ring below.

Thus, while it is plausible that Tce2 also span two rings, we cannot conclusively say that it is true for all Hcp cargos, and the precise stoichiometry is likely effector dependent. To be accurate, we have revised Fig. 7 to explicitly describe that in this model, it is Tce1 that spans two Hcp rings.

l. 232f "Western blot analysis revealed detectable expression of Tce2, but not Tce1". I am afraid I cannot entirely follow the authors in the text based on what I see in the figure. In Fig. 6f, isn't there a very faint band of Tse1 in the left panel that is not present in the empty vector control? I suggest to adjust the sentence above, which gave me the impression that Tce1 expression cannot be detected at all. There seems to be a bit more of Tce1 after co-expression with Hcp but just a bit more. To me, this could be described better in the text to reflect the observation more accurately.

Thank you for this observation. There is indeed small detectable amounts of Tce1 in the absence of Hcp3. We have revised the text to reflect this which now reads "Western blot analysis revealed detectable expression of Tce2, but only small amounts of Tce1, suggesting Tce1 may be unstable in this context" (lines 258-259).

l. 236/Fig. 6F Considering the presence of a very faint band of Tce1 full-length, it seems as if there is no band at all for Tce1CTD, potentially suggesting a role of the N-terminus in protein stability. No need to comment on this, just a thought.

We agree that it is possible that the lack of the Tce1 N-terminal domain may contribute to further destabilization.

L. 237 "and expressed in yeast in presence/absence of Hcp3" Might be worth checking this sentence again for the grammar.

Thank you, this has now been corrected in the text which now reads "We thus produced a N-terminally truncated version of Tce1 and expressed it in yeast in the presence/absence of Hcp3" (lines 263-264).

l. 238f "Overall, we identified for the first time a T6SS dependent antifungal property that may be associated with the H3-T6SS function through Tce2" I am a bit hesitant with this sentence. Technically speaking, I think it is correct. I am wondering if it could be misunderstood. What exactly does "for the first time" refer to here? T6SS-dependent antifungal properties in general have previously been shown. I understand that this is phrased with care because of the missing H3-T6SS activity in P. aeruginosa under laboratory conditions, it can therefore not be easily tested if the H3-T6SS of P. aeruginosa mediated antifungal activity. I would find a sentence more appropriate along the lines of: "Overall, we found antifungal properties of the Hcp3-associated effector Tce2, which is the first report of anti-fungal activity of an effector that is putatively secreted by the H3-T6SS and which suggests so far unknown anti-eukaryotic activity of the H3-T6SS."

We have replaced the sentence by the one suggested by the reviewer (lines 268-271).

L. 250ff "However, three specialized Hcp…" Wasn't the genomic screening that is referred to in the second half of the sentence also done by reference 42? In this case, wouldn't it be better

to refer to the reference at the end of this sentence. Also, one could argue about a study from 2017 being "recent", I leave this up to the authors.

We have now revised the discussion to address this suggestion (line 282-285, 340-344).

l. 256ff "Here our study reveals the detailed molecular mechanism on how cargo effectors are loaded into Hcp." Is it really sufficient data to state that the study "shows" a mechanism that applies across multiple diverse "cargo effectors"? Or does the sentence refer to "Tce1 cargo effectors"? I suggest to specify.

We have revised the sentence to specify that it relates to the Tce1 cargo (lines 292-293).

l. 264ff "In this process, S29, E56, and T60 serve as key hydrogen bond-forming residues, interacting with Tce1 across multiple Hcp3 protomers." To me, this came a bit out of the blue. Could this already be referred to in the corresponding results section and potentially be indicated in Fig. 4b, which residue engages in what kind of an interaction. Further, what was the rationale for listing these particular residues by name? Are these examples or is this an exhaustive list? If I understand the data correctly, isn't S31 also forming hydrogen bonds in multiple Hcps? Shouldn't this residue be listed by name here, too?

I see the value of referring to individual resides. On a higher level, the complexity could maybe be reduced. Isn't it basically about three sites of Hcp, one site around residue AA29-31, a second site around resides AA56-62, and a third site around roughly AA113-123 that matters for the interaction with Tce1 across multiple Hcp monomers?

We thank the reviewer for this helpful suggestion and agree that, at a higher level, the Hcp3-Tce1 interface can indeed be conceptualized as involving three main regions of Hcp3, centred approximately around residues 29-31, 56-62, and 113-123, which collectively mediate interactions across multiple Hcp protomers. We have revised the text to clarify that residue S31 also forms hydrogen bonds in several Hcp protomers (line 304).

At the same time, we believe it remains important to specify individual residues, as the exact side-chain identities differ between protomers and effectors and underpin the observed combination of conserved and effector-specific contacts. Explicitly identifying these residues allows a more precise comparison between Tce1 and Tce2 interactions and provides a framework for future mutational or functional analyses.

l. 267f "compared to the previously proposed mechanism" Could the authors please provide a reference?

We have revised the sentence to acknowledge prior work proposing that effectors are loaded into the lumen of Hcp rings (Silverman *et al*., *Mol Cell*, 2013), while clarifying that this model did not address Hcp–effector stoichiometry or the mechanism of loading. The revised text now emphasizes that our study provides mechanistic insight into a stepwise wrapping process (lines 324-325).

l. 279 "Instead, the extended Hcp may bind to 5 other 'regular' Hcps that complete the hexameric ring around one Hcp-ET." Maybe I missed it, wouldn't this sentence be an opportunity to refer to the preprint https://doi.org/10.1101/2025.01.09.632221 from earlier this year, that presents complementary data in support of this hypothesis?

We thank the reviewer for this opportunity to clarify. By "extended Hcp–toxin," we specifically refer to a chimeric architecture in which the toxin is physically fused to an Hcp subunit, forming a single extended polypeptide. The Hcp1–Hcp2–Bte1 complex described in the preprint

supports heterohexameric Hcp ring formation but does not describe such a chimeric Hcp–toxin fusion.

l. 301 “activity” is probably not supposed to be there.

We thank the reviewer for highlighting this. The word “activity” has now been removed.

Supplementary Table 2, Does the nomenclature of Hcp3-1 to -6 here refer to Hcp3-A to -F of the main figures? Would be easier for me to have one consistent nomenclature throughout.

We thank the reviewer for pointing this out. Yes, the nomenclature Hcp3-1 to -6 in Supplementary Table 2 corresponds to Hcp3-A to -F in the main figures. We apologize for this oversight and have now standardized the nomenclature throughout the manuscript for consistency.

Fig. 7 Is it possible that this figure is not being referred to in the text?

I overall agree with a graphical depiction that summarizes the findings on the mechanism of Hcp cargo effector loading. I am struggling a bit with how this is done here. First, doesn't the data of this study apply more to the assembly scenario and less to the disassembly scenario? Isn't the disassembly scenario much more speculative and as indicated here, does this take all the forces into account that might act on the system upon delivery into the target cell when breaching membranes? My suggestion would be to either exclusively focus on the steps before firing ore indicate more clearly in figure and text whether the authors think that their data applies to both parts, pre and post firing, with equal strength. Second, to me, the depiction seems mainly based on the finding of the interaction between an Hcp protein and effector protein at a 1:1 ratio and on concentration-dependent oligomerization. Could the finding on asymmetric interactions within the tube also be included graphically and could the individual findings be linked to each other (see major comment 1)? Considering the data on Bacteroides Hcp effectors of the preprint that suggests differences between species and systems in how many Hcp proteins they might have and how this number of Hcp proteins affects effector loading, I am wondering whether it is worth to either generalize this depiction to a point that it applies to both scenarios, the one described here for the H3-T6SS of P. aeruginosa and the one for Bacteroides, or to specify more clearly that the working model presented here applies to a scenario of only one Hcp protein being associated with this system, potentially writing out H3-T6SS and P. aeruginosa in the depiction or figure legend. I would argue that such a specification does not take away novelty and impact from this work.

We thank the reviewer for this detailed and helpful critique and apologies for the oversight in referring to Figure 7 in the text. The figure is now referred to in the Discussion (line 304, 325). We agree that our current working model is primarily supported by data on assembly prior to firing, whereas the post-firing disassembly scenario is more speculative. We have therefore revised the figure and legend to clearly distinguish between the experimentally supported pre-firing assembly steps and the more hypothetical post-delivery disassembly events, which are now explicitly labeled as hypothetical.

We have also revised the graphical model to more explicitly incorporate the asymmetric nature of Hcp-effector interactions, in addition to the 1:1 nucleation and concentration-dependent oligomerization. The updated figure now illustrates that initial capture occurs through a dominant asymmetric interface, followed by recruitment of additional Hcp protomers that engage the effector to different extents as the ring closes.

Finally, we agree that differences across systems should be acknowledged. Because our experimental data is derived specifically from the H3-T6SS of *P. aeruginosa*, we have now

explicitly indicated this in the figure and legend. We further note in the revised Discussion that systems such as those described in *Bacteroides* - which encode multiple Hcp homologs - may follow related but distinct cargo-loading strategies (lines 396-402).

Reviewer #3 (Remarks to the Author):

The manuscript by Paracuellos et al., "A Novel Molecular Mechanism for Effector Protein Wrapping and Delivery by the Bacterial Type VI Secretion System", describes a mechanism for export of cargo by the HCP component of this system. Hcp3 forms hexameric rings that stack to form a tube through which some T6S effectors are transported. Building on their previous work they show that two proteins, Tce1 and Tce2, interact with Hcp3 hexamers but also Hcp3 monomers and hexamer intermediates. They solve a high quality cryoEM reconstruction of an Hcp3:Tce1 complex, revealing that Tce1 occupies the lumen of the Hcp3 ring and interacts specifically via salt bridges and hydrogen bonds. The N-terminal domain of Tce1 protrudes from the ring, suggesting it may fill the hole of the next ring in the Hcp tube. Homology modeling suggests a similar interaction with Tce2. The authors propose that Hcp3 monomers bind to the Tce cargo and oligomerize to form hexamers that assemble into the Hcp tube, and that these Hcp rings dissociate once inside the host cell, releasing the cargo. This is an elegant, novel and highly plausible mechanism. Tce1 and 2 are homologs of cysteine proteases, and Tce2 is toxic when expressed in high levels in yeast, suggesting an antifungal activity for this effector. The results are compelling and represent a significant advance in understanding T6S biology. The manuscript is well-illustrated and well-written for the most part but would benefit from some clarifications and edits. These, along with some questions, are listed below as the issues appear in the paper rather than based on priority.

We thank the reviewer for this clear and supportive overview of our work.

Abstract: The authors mention immune cells in the first sentence so should clarify in the introduction whether bacterial T6S systems target such eukaryotic cells. Change "ca 120 Hcp ring assemblies" to "approximately 120 Hcp ring assemblies".

We thank the reviewer for this helpful suggestion. We have clarified in the introduction that bacterial T6SSs can target various eukaryotic cells (lines 46-48), and we have amended the wording in the Abstract accordingly, including changing "ca 120 Hcp ring assemblies" to "more than 100 Hcp ring assemblies" (line 30).

Line 67: "effectors to either one part" - change to "one component of"?

We have made the change (line 67).

Line 87: add "structure"

This has now been added (line 88).

Line 95-97: Sentence is awkward. Reword for clarity.

We thank the reviewer for pointing this out. We have reworded the sentence which now reads: "These insights may enable the rational engineering of T6SS-wielding bacteria for microbiome biocontrol in human and natural ecosystems" (lines 97-99).

Line 100-118: Explain here and in Fig. 1 legend what the source of the proteins is (i.e. from P. aeruginosa? Recombinant protein expressed in E. coli?) and which protein is His-tagged. Same for Line 122.

We thank the reviewer for flagging this and have now clarified in the main text and Figure 1 legend. On line 103, and lines 123-124, we indicate that Hcp3 and Tce1 originate from *P.*

aeruginosa PAO1 and were recombinantly expressed in *E. coli* BL21(DE3). We also specify that Hcp3 is His-tagged and Tce1 carries an HA tag (Hcp3–His and HA–Tce1) (lines 124-125).

Line 128: What does "following successful inspection" mean?

We thank the reviewer for pointing this out. We have revised the sentence to: "Upon purification, the assembly state and homogeneity of the complex were assessed by negative-stain EM. After confirming the presence of intact, monodisperse complexes, the sample was vitrified on thin carbon layer grids and imaged by cryo-EM" (lines131-132).

Line 141-143: Compare the cryoEM structure of the Hcp3 ring to the previously solved crystal structure of P. aeruginosa Hcp3 (PDB ID). Does the presence of Tce1 impact the Hcp3 ring structure? Are the Hcp3 protomers identical whether or not they contact Tce1?

We thank the reviewer for this point. Although a structure is available in the PDB that is sometimes annotated as Hcp3 from *P. aeruginosa*, it was in fact solved from the *hcpC* gene, which encodes Hcp2. In *P. aeruginosa*, three homologous genes encode Hcp2 (including *hcpC*), and thus this structure does not correspond to Hcp3. Consequently, a direct structural comparison between our cryo-EM Hcp3 ring and a crystallographic Hcp3 structure is not possible.

Line 146-147: Delete "Our model also showed that". Specify residue numbers for the C-terminal domain that was resolved.

We thank the reviewer for pointing this out. As suggested, we have removed "Our model also showed that" from the phrase (line 150). We have also specified the residue numbers corresponding to the resolved C-terminal domain in the revised text (line 152).

Line 163-182: Consistently refer to Hcp3 subunits within the ring as protomers rather than monomers. G121 and K123 are listed as Hcp3 residues that interact with Tce1, yet these are not shown in Fig. 4. In the Discussion (Line 263-266) residues S29, E56 and T60 as listed as being key. Describe the key interactions (eg. what does G121 contribute?). For Fig. 4, protomer labels are too small and the pale text is hard to read - add an outline/shadow. Could simplify Fig. 4a by labeling the panels the same as the protomers (eg. A, B, C etc).

We thank the reviewer for this point. G121 and K123 are in fact the most commonly interfacing residues with Tce1 when sat in the Hcp3 lumen and as such were optimally positioned for the generation of point mutations to validate our cryoEM structure (Fig 4b). S29, S31, E56 and T60 are indeed the residues involved in key hydrogen bond forming interactions with Tce1. The text in the section entitled "Tce1 interacts with key Hcp3 residues at the inner face of the ring" has been changed to reflect this. Fig. 4 and Fig. 4 legend has also been modified as per the reviewer's suggestions to outline the changes.

Line 216 (and Fig. 6 legend and elsewhere): Since proteolytic activity was not demonstrated, Tce1 and Tce2 should be called "putative" cysteine proteases. Did the authors attempt to identify Tce1/Tce2 substrates or demonstrate proteolytic activity?

We agree with the reviewer's point. As proteolytic activity was not directly demonstrated, we now refer to Tce1 and Tce2 as *putative* (or probable) cysteine proteases throughout the manuscript, including in the main text and Figure 6 legend. Identification of substrates and biochemical characterization of protease activity were beyond the scope of the present study and were therefore not pursued.

Line 246: Clarify the ambiguous statement that "a cargo domain that is an extension of a T6SS component". Why does the first paragraph of the Discussion focus on specialized effectors when Tce1 and Tce1 are cargo effector?

We agree with the reviewer's point and have revised the first paragraph of the discussion.

Line 247-249 - break into two sentences

We thank the reviewer for pointing this out. To improve clarity, and as indicated above, we have rewritten this paragraph.

Line 261-263: What evidence is there to support the statement that Hcp1 interaction with Tce1 nucleates hexamer formation? It also forms a hexamer in the absence of Hcp1.

The reviewer is raising an important point. We agree that Hcp can form hexamers in the absence of an effector, and we do not intend to imply that effector binding is strictly required for hexamerization. Rather, our data support the idea that Tce1 binding can *nucleate or bias* hexamer formation by stabilizing an initial Hcp–Hcp assembly intermediate. We have revised the text to clarify that effector binding promotes and stabilizes hexamer formation under the tested conditions, rather than being an absolute requirement for hexamer assembly (lines 302-304).

Line 276: "… than initially thought" - by whom?

We thank the reviewer for pointing this out. We have reworded the sentence which now reads "However, our work revealed that effectors can be enclosed by more than one Hcp ring, creating additional space to accommodate larger effectors than would be possible within the lumen of a single Hcp ring" (lines 332-334).

Line 281: "It is also striking that two effectors, Tce1 and Tce2, are packed within Hcp3 rings." Sounds like they are present at the same time - reword. Do the authors think that the Tce1 N-terminus interaction with the second Hcp3 ring is non-specific? Could an even longer effector be accommodated?

We thank the reviewer for pointing this out. We have reworded the sentence which now reads "It is also striking that each of the two effectors, Tce1 and Tce2, can be independently packaged within Hcp3 rings" (line 345-346).

We agree that the interaction between the Tce1 N-terminus and the second Hcp3 ring is likely to be largely non-specific and driven by spatial proximity rather than a defined binding interface. Our data suggest that this secondary encapsulation reflects the inherent capacity of stacked Hcp rings to accommodate extended cargo. In principle, even longer effectors could therefore be accommodated by additional Hcp rings, although there is likely to be an upper limit imposed by steric and assembly constraints in vivo. We have added a comment to reflect on this (lines 332-337).

Line 282 and elsewhere in the Discussion: The references to affinity should be qualified as affinity was not quantified.

We agree with the reviewer and have revised the text to avoid unqualified references to "affinity," clarifying that binding strength was not quantitatively measured.

Line 216-222: Are the Tce1 residues that interact with Hcp3 conserved in Tce2?

None of the residues from Tce1 that interact with Hcp3 are conserved in Tce2. This was evaluated from the sequence alignment of the two proteins and the interacting residues

determined through PDBePISA. There are conserved residues, however, from Hcp3 which interact with both effectors.

This is now clearly stated in the text. (line 242-247)

Line 324: “and will need” - what will need? Change to “It will be necessary to …”.

We have revised the sentence to replace “and will need” with “It will be necessary to …” (line 395).

Line 339 - “sequences … were cloned” - change to “genes” or “gene fragments” were cloned

We have replaced “sequences… were cloned” with “genes…. were cloned” (lines 413-414).

Fig. 5b: clarify that the concentrations listed are for Hcp3. What is the concentration of Tce1 in both 5a and 5b? Report in moles/molarity to show stoichiometry (or report molar ratios). Why does the hexamer peak disappear with lower Hcp3 concentrations but the monomer peak only appears at 0.1 mg/ml?

We thank the reviewer for these helpful comments. The concentrations listed in Fig. 5a,b refer to the total protein concentration. In Fig. 5a, only Hcp3 was analysed and the Tce1 concentration was therefore 0 µM. The Hcp3:Tce1 molar ratios are shown schematically above the 6:1 and 1:1 peaks. The 1:1 molar ratio was further verified by overlaying a chromatogram of molecular weight standards for the Superdex S200 column, confirming that the 15.4 mL peak corresponds to the mass of a 1:1 Hcp3–Tce1 complex (new Fig. 5b,). The monomer peak is observed only at 0.1 mg/mL, where Hcp3 predominantly exists as monomers due to its concentration-dependent oligomerisation. At 0.5 mg/mL and above, the equilibrium shifts toward the hexameric state. The molarity of the $Hcp3_{WT}$ hexamer in complex with Tce1 at 4.0 mg/ml and the $Hcp3_{WT}$ monomer in complex with Tce1 at 0.1 mg/ml were calculated to be 27.0 µM and 1.7 µM respectively. The molarity of Hcp3 and Tce1 at intermediate concentrations lies in between these two values. (new Fig. 5 a,b; line 205-207, 216-217).

Fig. 7 is not cited in the discussion. Fig. 7 should have two paths for Hcp hexamer formation, one with and one without cargo.

We thank the reviewer for this suggestion. We have now cited the revised Figure 7 in the Discussion (line 304, 325). In addition, we updated the figure to explicitly depict two assembly pathways for Hcp hexamer formation.

Throughout the manuscript, a space is needed between numbers and units (eg. 3.4 Å, 4 ml)

Thank you for noting this. We have corrected spacing throughout the manuscript to ensure a space is consistently included between numbers and units.

Reviewer #4 (Remarks to the Author):

I enjoyed reading this manuscript and commend the authors on important, challenging work.

We thank the reviewer for this very encouraging comment.

My review focuses on the antifungal effector aspect of the manuscript. The structural and mechanistic sections that precede this content represent the manuscript’s core strength and are supported by rigorous and well-executed experiments. The discovery that Tce2 is an antifungal effector is both interesting and novel, and the structural analysis of Tce1 loading via Hcp3 is particularly compelling. The cryo-EM reconstruction revealing a “cargo wrapping”

model, wherein Hcp monomers assemble around the effector, is a conceptually innovative contribution to the field. This mechanism is supported by complementary assays and sets the work apart from prior T6SS studies, which typically describe effectors as slotting into preformed tubes or attaching to the spike complex.

We also thank the reviewer for this comprehensive and adequate overview of our work.

However, while the evidence supporting Tce2's antifungal activity is strong, its delivery by the H3-T6SS remains unproven in a physiological context. The study relies on heterologous expression in Saccharomyces cerevisiae to assess toxicity, which bypasses the T6SS machinery. Although the authors acknowledge this limitation and use appropriately cautious language in their conclusions, the broader claim that H3-T6SS mediates antifungal targeting is currently inferential. Demonstrating T6SS-dependent delivery in a native context such as showing that a P. aeruginosa H3-T6SS mutant fails to kill yeast or fungi that wild-type strains can, would substantively strengthen this conclusion.

Two specific suggestions to address this gap include:

1.Performing direct co-culture assays using P. aeruginosa and a eukaryotic target (e.g S. cerevisiae or Candida albicans, or Candida glabrata to keep the simplicity of a yeast experiment to test for Tce2- and H3-T6SS-dependent killing would be essential. Failure of a P. aeruginosa Tce2 or H3 T6SS mutant to outcompete fungi in co-culture would support the conclusion.

This is a good point which we had previously assessed. We indeed performed an experiment in which we incubate yeast with Pseudomonas on a plate overnight at 30 degrees. We then scrape up the growth, resuspend the cells in LB containing Gentamicin (killing the *Pseudomonas* and preserving the yeast) and then plate on YPD with Gentamycin 30 μg/ml to determine CFUs. We could not see any difference between the parental strain (PA14 Δ*rsmA*)and its H3 null counterpart (PA14 Δ*rsmA* Δ*tssB*3). There is a killing of yeast (compared to the control), but in these conditions it is not H3-T6SS dependent. The failure to identify H3-T6SS-dependent killing also reflect on the fact that no H3-T6SS dependent secretion could be observed as discussed in response to the other reviewer's comments.

We have included these data here below for the reviewer consideration.

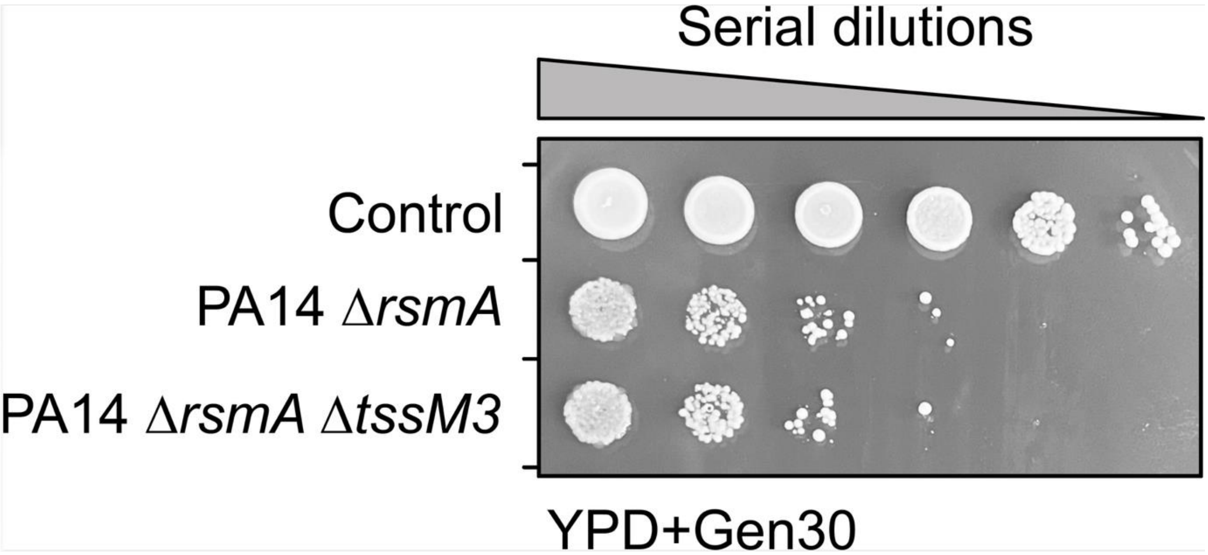

2.Investigating Tce2's mechanism of action more deeply, for instance via proteomic or genetic approaches in yeast to identify its cellular target, given its predicted cysteine protease activity would be a nice addition or future experiment.

Finally, the hypothesis that Tce1 requires auxiliary factors for activity is plausible and aligns with known dependencies of other T6SS effectors. Further work to identify potential cofactors or regulatory domains would help clarify its role. Overall, the antifungal component of the study is promising, though still preliminary in my opinion. The mechanistic work is a significant step forward, and with the additional experiment proposed above, the manuscript could establish a robust case for the inter-kingdom function of the H3-T6SS.

We thank the reviewer for these thoughtful suggestions and agree that identifying the cellular targets of Tce2 and potential auxiliary factors required for Tce1 activity would be highly informative and valuable directions for future work. However, such proteomic and genetic approaches would require a substantial, dedicated investigation that goes well beyond the scope of the present study, which is focused on uncovering the structural and mechanistic basis of Hcp–effector encapsulation and loading. Accordingly, while our antifungal data provide an initial functional indication of inter-kingdom activity, we view this component as a foundation for future in-depth mechanistic studies rather than a comprehensive characterization of effector function. We added a sentence in the discussion to make this point clearer (lines 389-391).

Minor points:

The crossbow analogy used to describe the T6SS mechanism is engaging, but the narrative oscillates between lay terminology and technical jargon. A more consistent register would improve clarity and accessibility for readers.

Protein descriptors at their first mention would improve readability and framing.

We have reviewed our text all through and adapted the descriptors for consistency.
